# *Schistosoma mansoni* Tegument Protein Sm29 Is Able to Induce a Th1-Type of Immune Response and Protection against Parasite Infection

**DOI:** 10.1371/journal.pntd.0000308

**Published:** 2008-10-01

**Authors:** Fernanda C. Cardoso, Gilson C. Macedo, Elisandra Gava, Gregory T. Kitten, Vitor L. Mati, Alan L. de Melo, Marcelo V. Caliari, Giulliana T. Almeida, Thiago M. Venancio, Sergio Verjovski-Almeida, Sergio C. Oliveira

**Affiliations:** 1 Departamento de Bioquímica e Imunologia do Instituto de Ciências Biológicas da Universidade Federal de Minas Gerais, Belo Horizonte, Minas Gerais, Brazil; 2 Departamento de Morfologia do Instituto de Ciências Biológicas da Universidade Federal de Minas Gerais, Belo Horizonte, Minas Gerais, Brazil; 3 Departamento de Parasitologia do Instituto de Ciências Biológicas da Universidade Federal de Minas Gerais, Belo Horizonte, Minas Gerais, Brazil; 4 Departamento de Patologia do Instituto de Ciências Biológicas da Universidade Federal de Minas Gerais, Belo Horizonte, Minas Gerais, Brazil; 5 Departamento de Bioquímica, Instituto de Química, Universidade de São Paulo, São Paulo, Brazil; George Washington University, United States of America

## Abstract

**Background:**

Schistosomiasis continues to be a significant public health problem. This disease affects 200 million people worldwide and almost 800 million people are at risk of acquiring the infection. Although vaccine development against this disease has experienced more failures than successes, encouraging results have recently been obtained using membrane-spanning protein antigens from the tegument of *Schistosoma mansoni.* Our group recently identified Sm29, another antigen that is present at the adult worm tegument surface. In this study, we investigated murine cellular immune responses to recombinant (r) Sm29 and tested this protein as a vaccine candidate.

**Methods and Findings:**

We first show that Sm29 is located on the surface of adult worms and lung-stage schistosomula through confocal microscopy. Next, immunization of mice with rSm29 engendered 51%, 60% and 50% reduction in adult worm burdens, in intestinal eggs and in liver granuloma counts, respectively (p<0.05). Protective immunity in mice was associated with high titers of specific anti-Sm29 IgG1 and IgG2a and elevated production of IFN-γ, TNF-α and IL-12, a typical Th1 response. Gene expression analysis of worms recovered from rSm29 vaccinated mice relative to worms from control mice revealed a significant (q<0.01) down-regulation of 495 genes and up-regulation of only 22 genes. Among down-regulated genes, many of them encode surface antigens and proteins associated with immune signals, suggesting that under immune attack schistosomes reduce the expression of critical surface proteins.

**Conclusion:**

This study demonstrates that Sm29 surface protein is a new vaccine candidate against schistosomiasis and suggests that Sm29 vaccination associated with other protective critical surface antigens is the next logical strategy for improving protection.

## Author Summary

Schistosomiasis is the most important human helminth infection in terms of morbidity and mortality. Although the efforts to develop a vaccine against this disease have experienced failures, a new generation of surface antigens revealed by proteomic studies changed this scenario. Our group has characterized the protein Sm29 described previously as one of the most exposed and expressed antigens in the outer tegument of *Schistosoma mansoni*. Studies in patients living in endemic areas for schistosomiasis revealed high levels of IgG1 and IgG3 anti-Sm29 in resistant individuals. In this study, confocal microscope analysis showed Sm29 present in the surface of lung-stage schistosoluma and adult worms. Recombinant Sm29, when used as vaccine candidate, induced high levels of protection in mice. This protection was associated with a typical Th1 immune response and reduction of worm burden, liver granulomas and in intestinal eggs. Further, microarray analysis of worms recovered from vaccinated mice showed significant down-regulation of several genes encoding previously characterized vaccine candidates and/or molecules exposed on the surface, suggesting an immune evasion strategy of schistosomes under immune attack. These results demonstrated that Sm29 as one of the important antigens with potential to compose a vaccine against schistosomiasis.

## Introduction

Schistosomiasis mainly occurs in developing countries and is the most important human helminth infection in terms of global mortality. This parasitic disease affects more than 200 million people worldwide causing more than 250,000 deaths per year [1]. Furthermore, schistosomiasis causes up to 4.5 million DALY (disability adjusted life year) losses annually [2]. Recently, King et al [3] have associated schistosomiasis with anemia, pain, diarrhea, exercise intolerance, and under-nutrition that results from chronic infection. Current schistosomiasis control strategies are mainly based on chemotherapy but, in spite of decades of mass treatment, the number of infected people remains constant [4]. Extensive endemic areas and constant reinfection of individuals together with poor sanitary conditions in developing countries make drug treatment alone inefficient [5]. Many consider that the best long-term strategy to control schistosomiasis is through immunization with an anti-schistosomiasis vaccine combined with drug treatment [6]. A vaccine that induces even a partial reduction in worm burdens could considerably reduce pathology and limit parasite transmission [7].

Sm29 protein has been characterized by our group [8], proving to be a membrane-bound antigen on adult worms that is strongly recognized by IgG1 and IgG3 antibodies of naturally resistant individuals and patients resistant to re-infection living in endemic areas for schistosomiasis in Brazil. Recent studies, using microarrays and RT-PCR, showed that Sm29 is among the 16% most highly expressed genes in *S. mansoni*, and probably serves an important function in the surface biology of this parasite [9],[10]. Proteomic analysis of the *S. mansoni* tegument composition identified Sm29 as one of the integral proteins to be consistently found in the outer surface [11]. Therefore, the next step would be to investigate humoral and cellular immune responses induced by Sm29 in vaccinated mice and protection studies.

Herein, we determined that Sm29 is present on the tegument of lung-stage and male and female adult worms of *S. mansoni* by confocal microscopy. Besides, rSm29 induced a Th1-type of immune response in mice and reduction in worm burden and liver pathology.

## Methods

### Mice and parasites

C57BL/6 and TLR4 KO female mice, 6–8 weeks old, were obtained from the Federal University of Minas Gerais (UFMG) animal facility. All procedures involving animals were approved by the local Ethics Committee on Animal Care (CETEA-UFMG). Cercariae of *S. mansoni* (LE strain) were maintained routinely in *Biomphalaria glabrata* snails at Rene Rachou Research Center (Fiocruz, Brazil) and prepared by exposing infected snails to light for 2 hrs to induce shedding of parasites. Cercarial numbers and viability were determined using a light microscope prior to infection. The protocols involving animals used in this study were approved by the Federal University of Minas Gerais Ethics Committee in Animal Experimentation (CETEA No. 023/2005).

### Antigen preparation

The recombinant Sm29 was produced and purified as described previously [8]. Briefly, the Sm29 cDNA fused with a C-terminal 6x histidine was produced in *E.coli* using the pET21a expression vector (Novagen, NJ, USA). The recombinant Sm29 was purified in an affinity column and dialyzed against PBS pH 7.0.

### Immunolocalization of Sm29 in male, female and lung-stage schistosomula of S. *mansoni*

Adult worms used in confocal microscopy studies were recovered from perfused mice and lung-stage schistosomula were prepared according to the method described by Harrop & Wilson [12]. Parasites fixed in Omnifix II (Ancon Genetics, St Petersburg, FL, USA) were used in whole mount or in section assays. For sections assays, 7 μm slices were deparaffinized with xylol series. Parasites were blocked with 1% BSA in PBST (Tween 20 0.05%) for 1 hr and incubated with anti-rSm29 serum diluted 1∶20 in blocking buffer. Serum from non-immunized mice was used as a negative control. Samples were washed three times with PBST and incubated with anti-mouse IgG antibody conjugated to Alexa Fluor 594 (Molecular Probes, CA, USA) diluted 1∶100 in blocking buffer containing Phalloidin Alexa Fluor 488 (Molecular Probes) to stain actin microfilaments. The samples were washed four times and mounted in antifade reagent (Prolong gold–Molecular Probes). For whole mount assays, parasites were blocked with 1% BSA, 0.1% Triton X-100, 0.1% azide in PBS for two hours at 4°C under agitation. Blocked parasites were incubated with anti-Sm29 serum diluted 1∶80 in the blocking buffer during 16hrs at 4°C under agitation. Samples were washed six times with PBST and incubated with anti-mouse and phalloidin, both diluted 1∶300, during 4 hours at 4°C under agitation. The samples were washed five times and mounted in 90% glycerol and 10% TRIS, pH 9.0. The parasites were visualized in a Zeiss 510 Meta confocal microscope using an immersion objective. All the parameters and microscope settings used were maintained throughout the process.

### Tegument purification

Cercariae obtained from *Biomphalaria glabrata* snails exposed to light for two hours were transformed mechanically in skin-stage schistosomula according to Ramalho-Pinto et al [13]. First, cercariae were incubated on ice for 30 minutes and centrifuged for 3 minutes, 1000 rpm, 4°C. The cercariae were resuspended in 1ml of cold ELAC (Earle's salts plus lactalbumin hydrolysate) contain 0.5% lacto albumin, 1% penicillin/streptomycin and 0.17% glucose. The tails were broken by vortex in high speed during 2 minutes. After this, the tails were removed through six washes with ELAC. The schistosomula were incubated for 1 hour and 30 minutes at 37°C in ELAC and washed with apyrogenic physiologic saline. For the tegument removal, the schistosomula were submitted to vortex using high speed, two times of eight minutes each, in a CaCl_2_ 0.3 M solution. The sample was centrifuged at 1000 rpm for 1 minute. The supernatant was collected and centrifuged at 26500 rpm for 1 hour at 4°C. The pellet was dialyzed against physiologic saline 1.7%.

### Western blot analysis

Two micrograms of purified skin-stage schistosomula tegument were submitted to SDS- PAGE 12%, transferred to a nitrocellulose membrane (Millipore) and blocked 16 hours at room temperature with TBST (0.5M NaCl, 0.02M Tris pH 7.5, 0.05% tween, 5% skim milk). The membranes were washed tree times with TBST and probed with anti-Sm29 or naive mice serum diluted 1∶500 in TBST for 4 hours at room temperature. The membranes were washed six times and probed one hour with anti-mouse IgG conjugated to alkaline phosphatase (Invitrogen) diluted 1∶10000 in TBST. After six washes, the membranes were developed using ECF subtract (GE Healthcare) and visualized in a Storm apparatus (GE Healthcare).

### Immunization of mice

Six to eight week old female C57BL/6 mice were divided into two groups of ten mice each. Mice were subcutaneously injected in the nape of the neck with 25 μg rSm29 on days 0, 15 and 30. Sm29 protein concentration for vaccination was determined as previously described [14]. The recombinant protein was formulated with Freund's adjuvant (complete Freund's adjuvant/CFA for the first immunization and incomplete Freund's adjuvant/IFA for the boosters). In the control group, PBS with Freund's adjuvant was administered using the same immunization protocol.

### Challenge infection and worm burden recovery

Fifteen days after the last boost, mice were challenged through percutaneous exposure of abdominal skin for 1 h in water containing 100 cercariae (LE strain). Forty-five days after challenge, adult worms were perfused from the portal veins. Two independent experiments were performed to determine protection levels. The protection was calculated by comparing the number of worm recovered from each vaccinated group with its respective control group, using the formula:
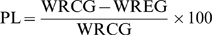
where PL = protection level, WRCG = worms recovered from control group, and WREG = worms recovered from experimental group.

### Oogram and histophatological analysis

Following perfusion for the recovery of the schistosomes, fragments of the intestine (terminal ileum) from each animal were separated and transferred to the Petri dishes containing saline as previously demonstrated [15]. The intestines were opened lengthwise and the excess mucus was removed. One-centimeter fragments were weighed and placed between a glass slide and a plastic cover. The preparation was inverted and pressed on a rubber surface padded with filter paper. Then, the slices were examined with a microscope (100 x) and all the eggs were counted. Quantitative oograms were obtained, using the formula: number of eggs/grams of tissue = number of eggs in intestinal fragment/weight of fragment in mg. A qualitative oogram evaluation was performed, in which the developmental stages of the eggs were classified as described previously.

The liver fragments were collected from the same animals analyzed in oogram studies and fixed in 10% paraformaldehyde. The fragments were processed for paraffin embedding and histopathological sections performed using microtome at 6–7 μm and stained in a slide with hematoxilin-eosin (HE). The number of granulomas was obtained from the liver sections using 10x objective in a microscope. The area from each liver section was calculated using capture in scanner followed by analysis in the KS300 software connected to a Carl Zeiss image analyzer.

### Measurement of specific anti-Sm29 antibodies

Following immunization, sera of ten mice from each experimental group were collected at two-week intervals. Measurement of specific anti-Sm29 antibodies was performed using indirect ELISA. Maxisorp 96-well microtiter plates (Nunc, Denmark) were coated with 5 μg/ml rSm29 in carbonate-bicarbonate buffer, pH 9.6 for 16 h at 4°C, then blocked for 2 at room temperature with 200 μl/well PBST (phosphate buffer saline, pH 7.2 with 0.05% Tween-20) plus 10% FBS (fetal bovine sera). One hundred microliters of each serum diluted 1∶100 in PBST was added per well and incubated for 1 h at room temperature. Plate-bound antibody was detected by peroxidase-conjugated anti-mouse IgG, IgG1 and IgG2a (Southern Biotechnology, CA, USA) diluted in PBST 1∶10000, 1∶5000 and 1∶2000, respectively. Color reaction was developed by addition of 100 μl per well of 200 pmol OPD (*o*-phenylenediamine, Sigma) in citrate buffer, pH 5.0 plus 0.04% H_2_O_2_ for 10 min and stopped with 50 μl of 5% sulfuric acid per well. The plates were read at 495 nm in an ELISA plate reader (BioRad, Hercules, CA).

### Cytokine analysis

Cytokine experiments were performed using splenocyte cultures from individual mice immunized with rSm29 plus CFA/IFA (*n* = 5 for each group). Splenocytes were isolated from macerated spleens of individual mice 10 days after the third immunization and washed twice with sterile PBS. After washing, the cells were adjusted to 1×10^6^ cells per well for IL-4, IL-10, IFN-γ and TNF-α assays in RPMI 1640 medium (Gibco, CA, USA) supplemented with 10% FBS, 100 U/ml penicillin G sodium, 100 μg/ml streptomycin sulfate, 250 ng/ml amphotericin B. Splenocytes were maintained in culture with medium alone or stimulated with rSm29 (25 μg/ml) or concanavalin A (ConA) (5 μg/ml) as previously described [14],[16]. The 96-well plates (Nunc) were maintained in an incubator at 37°C with 5% CO_2_. For cytokine assays polymyxin B (30 μg/ml) was added to the cultures and this treatment completely abrogate the cytokine response to LPS as previously described [17]. Culture supernatants were collected after 24 h of ConA stimulation, 48 h of rSm29 stimulation for IL-4 and TNF-α analysis and 72 h of rSm29 stimulation for IL-10 and IFN-γ. The assays for measurement of IL-4, IL-10, IFN-γ and TNF-α were performed using the Duoset ELISA kit (R&D Diagnostic) according to the manufacturer's directions.

Innate immune responses were assessed using peritoneal macrophages culture obtained from C57BL/6 and TLR4 KO mice that had received an intra-peritoneal injection of thioglycolate four days earlier. The macrophages were recovered through peritoneal washes with PBS and adjusted to 1×10^6^ cells per well for IL-12p40 assays in RPMI 1640 medium (Gibco) supplemented with 5% FBS, 100U/ml penicillin G sodium and 100 μg/ml streptomycin sulfate. Macrophages were maintained in culture with medium alone or stimulated with rSm29 (25 μg/ml) or LPS purified from *E. coli* (1 μg/ml). Culture supernatants were collected after 24 hrs of stimulation. The assays for measurement of IL-12p40 were performed using the Duoset ELISA kit (R&D Diagnostic) according to the manufacturer's directions.

### Microarray analysis of gene expression in *S. mansoni* worms

Total RNA was obtained using Trizol reagent (Invitrogen) according to the manufacturer's instructions. RNA was quantified using the Nanodrop ND-1000 UV/Vis spectrophotometer and RNA integrity was checked by electrophoresis with an Agilent 2100 Bioanalyzer. One microgram of total RNA from each sample was amplified using the T7-RNA polymerase-based linear amplification protocol. Three micrograms of aminoallyl-modified amplified RNA was labeled with Cy3 or Cy5 by indirect labeling (GE Healthcare).

The Cy3- and Cy5-labeled RNA samples were combined and hybridized overnight to cDNA microarray slides using ASP hybridization chambers (GE Healthcare) at 42°C and the protocols were followed as recommended by manufacturer [18]. Slides were scanned at 5 μm resolution, 100% laser power (GenePix 4000B, Molecular Devices, USA) and PMT levels were adjusted in order to obtain similar average intensities of red and green signals. Each array contained 4,000 different elements, spotted in duplicate on the left and the right sides of a glass slide (GEO accession: GPL3929). The cDNA clones spotted on the arrays were selected to represent 4,000 unique transcripts that were identified in the large-scale *S. mansoni* transcriptome project [19].

RNA from the pool of adult worms recovered from mice that had been immunized with the rSm29 vaccine (test sample) was hybridized against RNA from control adult worms recovered from mice treated only with adjuvant (control sample). Each assay consisted of two separate hybridizations, the first with Cy5-labeled test sample and Cy3-labeled control, and the second with dye-swap in order to account for dye bias; for each probe, the intensity of the test sample was computed as the average intensity of the Cy5 and Cy3 measurements; the same was computed for the control sample. Each assay provided two average values for the test and two for the control, arising from the duplicated copies of each gene that are spotted on the left and right halves of the glass slides [18]. A total of four hybridizations were performed, corresponding to two sets of technical replicate assays. Thus, a total of four different average intensity values for the test and four for the control sample were obtained for each probe on the array.

Data was extracted from images using the Array Vision 8.0 program. We used a locally weighted linear regression (LOWESS) algorithm to correct for systematic bias due to small differences in the labeling and/or detection efficiencies between the fluorescent dyes [20]. Normalized log_2_ ratios of the intensities of test/control were used for the statistical analysis with the Significance Analysis of Microarrays tool (SAM) using a 0.14% false discovery rate (FDR), which corresponds to a q-value (analogous to the t-test p-value) of q<0.01, to find significant differentially expressed genes [21]. Genes determined in the previous step were filtered using a median fold-change ≥1.5 as cutoff, where fold-change = 2ˆ|log2 (test/control)|. The fold-change filter was applied after identification of significant differentially expressed genes with the use of SAM, therefore avoiding the bias caused by the use of arbitrary thresholds to trim datasets before significance testing [22].

### Putative identity of regulated genes

Regulated genes were manually annotated by similarity using BLASTx and the GenBank nr database with a cut-off e-value = 10^−10^. Sequences were placed into four categories according to the similarity matches, as follows: tegument proteins, membrane proteins, receptor interacting proteins, lipid metabolism and other functions.

### Real-time RT-PCR

Total RNA was extracted with Trizol reagent (Invitrogen), according to the manufacturer's instructions. RNA samples were treated with DNAse, purified and concentrated using RNeasy Micro Kit (QIAGEN), following the manufacturer instructions and 1.5 μg of each sample was reverse transcribed using the SuperScript® III First-Strand Synthesis SuperMix (Invitrogen). Specific primer pairs (Table S2) were designed by Primer Express Program using default parameters (Applied Biosystem) and arbitrarily named primers 1 and 2. Real-time RT-PCRs were run in triplicates in a volume of 20 μl containing 10 μl of Sybr Green PCR Master Mix (Applied Biosystem), 160 nmol of each primer (primers 1 and 2), 0.30 μl cDNA from reverse transcription. Real-time RT-PCR was performed with the 7300 Real-Time PCR System (Applied Biosystem) using the following cycling parameters: 60°C for 10 min, 95°C for 10 min, 40 cycles of 95°C for 15 sec and 60°C for 1 min, and a dissociation stage of 95°C for 15 sec, 60°C for 1 min, 95°C for 15 sec, 60°C for 15 sec. Real time data was normalized in relation to the level of expression of actin. *p*-values were determined for the triplicates with Student's *t*-test, using one tail distribution and heteroscedastic variance.

### Microarray data public deposition information

All microarray data were deposited in the GEO public database under Accession No. GSE10777.

### Statistical analysis

Statistical analysis was performed with Student's *t-*test for comparison between two experimental groups using the software package GraphPad Prism.

## Results

### Sm29 is a tegument protein of *S. mansoni*

**Figure 1:**
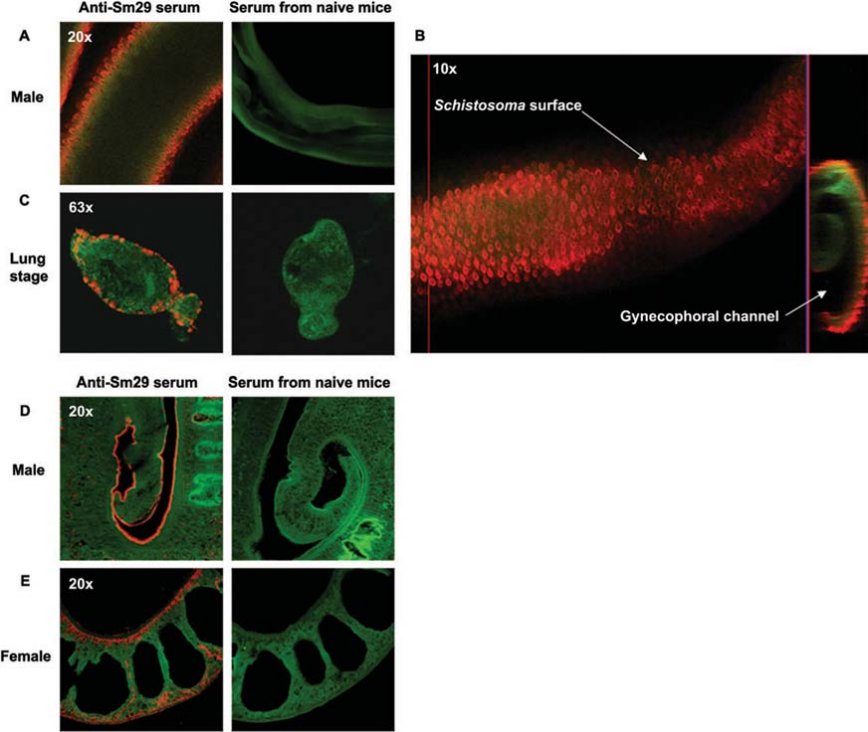
Immunolocalization of Sm29 antigen on male and female adult worm and lung-stage schistosomula of *S. mansoni*. Polyclonal anti-Sm29 antibodies, serum from mice that received Freund́s adjuvant as negative control, and Cy5-conjugated anti-mice IgG were used. Actin was visualized by falloidin-Alexa fluor 488. The parasites were fixed in Omnifix II and used to whole-mount or section immunolocalization. (A and B) Whole-mount immunolocalization of Sm29 antigen on the surface (outer tegument) of male adult worm and (C) lung-stage schistosomula of *S. mansoni.* (D) Immunolocalization of Sm29 on the surface (outer tegument) of male adult worm, and (E) on the surface (outer tegument) and in some internal tissues on the female adult worm using deparaffinized sections of the parasites. Localization of Sm29 is identified by the orange color and actin filaments by the green color.

**Figure 2:**
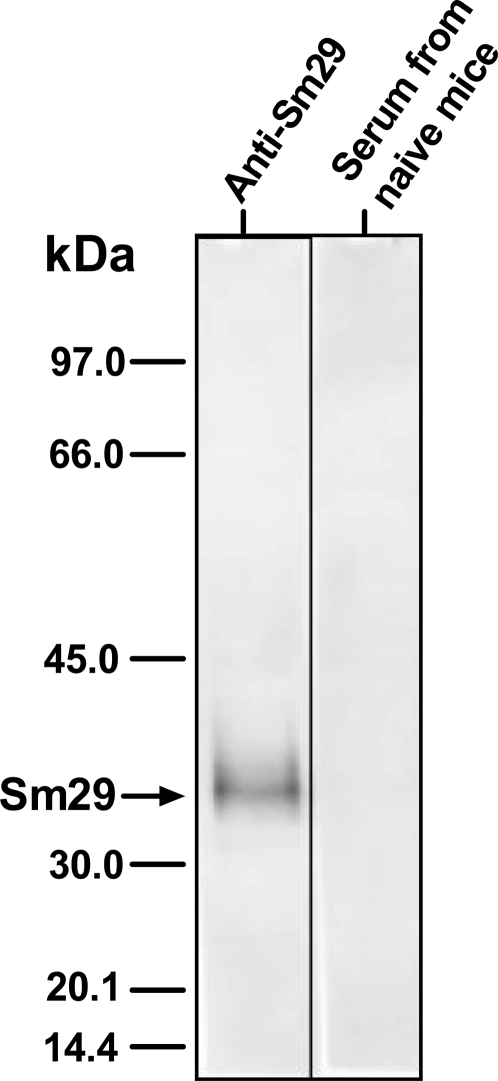
Identification of Sm29 on skin-stage schistosomula tegument by Western blot. Two micrograms of purified skin-stage schistosomula tegument was applied onto 12% SDS-PAGE and transferred to a nitrocellulose membrane by western blot. After that, the membrane was probed with serum from rSm29 vaccinated mice or naïve animals diluted 1∶500 in TBST. Arrow indicates the native Sm29. The molecular mass markers, from top to bottom, are: 97, 66, 45, 30, 20.1, and 14.4 kDa.

In the present study, we demonstrated the localization of Sm29 in the male and female adult worms and also in the lung-stage schistosomula of *S. mansoni* using specific antibodies to rSm29 produced in *E. coli*. The native Sm29 was located exclusively on the surface of lung-stage schistosomula and male adult worms of *S. mansoni* (Fig. 1A, B, C, D, and Video S1). The female adult worm showed Sm29 located on the surface and also in internal tissues (Fig. 1E). Confocal microscopy analysis also revealed that Sm29 is not present in the cercariae stage of *S. mansoni* (Figure S1), as expected from the absence of mRNA coding for this antigen in cercariae cDNA library [8]. Additionally, we detected Sm29 in the purified tegument fraction of the skin-stage schistosomula by western blot analysis using specific antibodies to rSm29 (Fig. 2).

### Antibody profile following mice immunization

**Figure 3:**
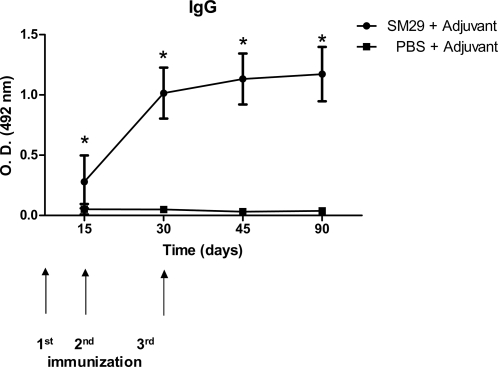
Kinetics of specific anti-Sm29 IgG induced in mice immunized with rSm29. Sera of ten immunized mice per group were collected at days 15, 30, 45, and 90 after the first immunization and assayed by ELISA. Arrows indicate the timing of vaccination. Results are presented as the mean absorbance measured at 492nm for each group. Results represent the mean of two independent experiments. Statistically significant differences of vaccinated mice compared to PBS+adjuvant control group is denoted by one asterisk for (p<0.05).

To evaluate the level of specific IgG, IgG1 and IgG2a antibodies to Sm29 sera from ten vaccinated animals of each group were tested by ELISA. Significant titers of specific anti-Sm29 IgG antibodies were detected at all time points studied after the first immunization (Figure 3). In order to determine the IgG isotype profile induced by vaccination, specific anti-Sm29 IgG1 and IgG2a antibodies levels were also evaluated. The levels of specific IgG1 and IgG2a increased at 15, 30 and 45 days after the first immunization (Table 1). Furthermore, IgG1/IgG2a ratio was reduced at days 30 and 45 that parallels with elevated anti-Sm29 IgG2a production. The IgG1/IgG2a ratio observed in mice immunized with rSm29 can lead us to speculate that a Th1 type of immune response was induced following vaccination.

**Table 1:** IgG1 and IgG2a immune profile induced by vaccination with recombinant Sm29.

Days^a^	Groups				
	IgG1		IgG2a		IgG1/IgG2a ratio
	Sm29	PBS	Sm29	PBS	Sm29
15	0.821±0.559*	0.034±0.022	0.220±0.102*	0.011±0.081	3.72
30	1.290±0.326*	0.006±0.068	0.637±0.228*	0.049±0.070	2.02
45	1.623±0.159*	0.006±0.007	0.812±0.267*	0.031±0.017	1.99

a Days after the first immunization

*: Statistically significant compared to group of animals immunized with PBS with p<0.05

### Vaccination induces a Th1-type of immune response in mice

**Figure 4:**
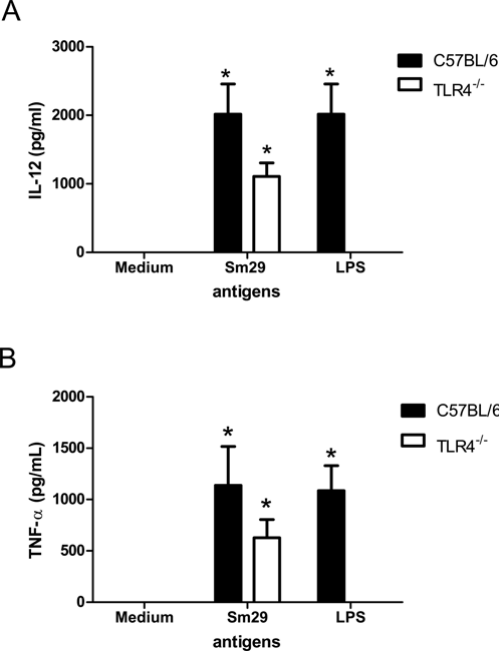
Sm29 induces IL-12 and TNF-α production by macrophages from TLR4 knockout mice. Levels of IL-12 (p40) (A) or TNF-α (B) were measured in the supernatants of inflammatory macrophages from TLR4 KO or wild-type mice stimulated for 24 hrs with rSm29 (25 μg/ml), *E. coli* LPS (1 μg/ml) or medium alone. Significant differences from stimulated TLR4 KO or C57BL/6 macrophages in relation to non-stimulated cells are denoted by an asterisk (*) for p<0.05.

Mice vaccinated with rSm29 showed elevated production of IFN-γ, TNF-α and IL-10 and absence of IL-4 (Table 2). Cultures used for measuring the cytokines were treated with polymyxin B to avoid non-specific stimulation due to eventual LPS contamination in the purified recombinant protein [17]. Additionally, rSm29 has stimulated peritoneal macrophages from C57BL/6 (2016±423 pg/ml) or TLR4 KO (1108±197 pg/ml) mice to produce IL-12 (Fig. 4). It is worth to emphasize that rSm29 has activated macrophages to secrete IL-12 which drives Th1 cell development. Protective immunity in mice against *S. mansoni* infection, before the onset of egg production, is thought to be the result of a Th1 pattern of immune response [23].

**Table 2:** Parasitologic data, cytokine profile, intestinal egg counts and liver granuloma analysis of C57BL/6 mice vaccinated with rSm29 and challenged with 100 *Schistosoma mansoni* cercariae.

Groups a	Cytokine profile in splenocytes (pg/ml)		Male worms Mean±SD	Female worms Mean±SD	Total worms Mean±SD (% protection)	Intestinal eggs Mean±SD (% reduction)	Number of granulomas/liver tissue μm^2^ 10^−7^ Mean±SD (% reduction)
**PBS+CFA/IFA**	IL-4	7.8±3.0	28.2±8.1	29.4±6.4	57.7±12.2	16110.3±5755.2	6.74±1.3
	IFN-γ	31.2±5.1					
	TNF-α	15.6±4.2					
	IL-10	31.3±3.1					
**rSm29+CFA/IFA**	IL-4	7.8±4.6	14.6±1.7	15.1±4.7	29.7±5.3* (51%)	6440.4±3644.2* (60%)	3.3±0.6* (50%)
	IFN-γ	2538.3±44.6 *					
	TNF-α	426.3±231.5*					
	IL-10	425.2±92.2*					

a This Table shows one experiment representative of two independent trials with similar results

*: Statistically significant compared to the control group (*p*<0.05)

### rSm29 engenders protective immunity

To test the vaccine potential of rSm29 against schistosomiasis, we investigated the protection induced by this recombinant antigen in the murine model of *S. mansoni* infection. C57BL/6 mice were immunized three times with adjuvant-formulated rSm29, and then challenged with 100 *S. mansoni* cercariae. Control groups received adjuvanted phosphate-buffered saline. Mice vaccinated with rSm29 showed 51% reduction in adult worm burden, 60% reduction in intestinal eggs and 50% reduction in liver granuloma counts compared to control mice (Table 2). The most critical points to be considered about an efficient anti-schistosomiasis vaccine are the reduction in pathology and transmission [7], aspects that are present in the protection profile induced by rSm29 vaccine.

### Gene expression profile in worms from vaccinated mice

**Figure 5:**
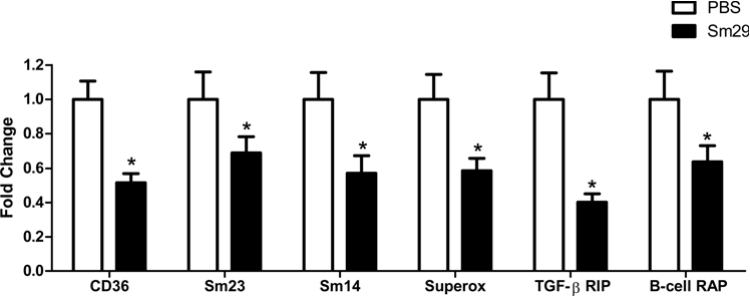
Transcript level changes in selected genes of *S. mansoni* worms recovered from mice vaccinated with rSm29. The figure shows real-time RT-PCR validation experiment for six genes down-regulated in *S. mansoni* worms recovered from rSm29+adjuvant immunized mice (black bars) compared to worms from PBS+adjuvant injected control mice (white bars). The selected genes were: CD36-CD36-like class B scavenger receptor [*S. mansoni*]; Sm23-Sm23 kDa integral membrane protein [*S. mansoni*]; Sm14-Fatty acid-binding protein Sm14 [*S. mansoni*]; Superox-Superoxide dismutase [*S. mansoni*]; TGF-β RIP-TGF-β receptor interacting protein 1 [*C. sinensis*]; B-cell RAP-B-cell receptor-associated protein-like protein [*S. mansoni*]. Statistically significant differences of vaccinated mice compared to PBS+adjuvant control group is denoted by one asterisk (p<0.03).

In order to investigate the impact of rSm29 vaccination on *S. mansoni*, worms that were recovered from vaccinated mice had their gene expression profile analyzed using microarrays. This analysis revealed significant (q<0.01) regulation of 517 genes (Table S1) in worms recovered from mice vaccinated with adjuvanted rSm29 when compared to control mice injected with adjuvanted phosphate-buffered saline; of these, 495 genes (96%) were down-regulated and only 22 genes (4%) were up-regulated. Among down-regulated genes, several of them caught our attention and they fell into four major categories: tegument proteins (Sm23 and CD36-like class B scavenger receptor), membrane proteins (tetraspanin TE736), lipid metabolism (Sm14), receptor interacting proteins (B-cell receptor-associated protein and TGF-beta receptor interacting protein) and others (superoxide dismutase and Sm65) (Table 3). To validate the microarray data, we selected six genes (Sm23, Sm14, superoxide dismutase, CD36-like class B scavenger receptor, B-cell receptor-associated protein and TGF-β receptor interacting protein) and performed real-time RT-PCR. As demonstrated in Figure 5, real-time RT-PCR of all six tested genes confirmed the reduced expression previously determined by microarray analysis. Based on the data shown in Table 3 and Figure 5, we hypothesize that in rSm29 protected mice a reduced expression of these critical surface antigens enables the parasites to adapt to the host in the face of an ongoing antiparasite immune response developed in vaccinated animals.

**Table 3:** Putative identity, categories of biological function and fold change of a subset of down-regulated genes identified in worms recovered from animals vaccinated with rSm29 associated to adjuvant.

Contig	Putative identity	Fold Change^a^ Down-regulation with rSm29 plus adjuvant
Tegument proteins		
C600716.1	CD36-like class B scavenger receptor [S. mansoni]	1.7
C605987.1	Integral membrane protein/sugar transporter family [B. vulgaris]	2.1
C601295.1	Sm23 kDa integral membrane protein [S. mansoni]	2.0
C607243.1	Alkaline phosphatase [M. musculus]	3.1
C610121.1	Annexin [S. mansoni]	2.0
Membrane proteins		
C604563.1	Tetraspanin TE736 [S. japonicum]	2.1
C609525.1	Transmembrane protein 49 [X. tropicalis]	2.6
C605890.1	ATPase, aminophospholipid transporter [B. Taurus]	1.9
C606907.1	Putative insulin receptor [E. multilocularis]	1.8
C609637.1	vacuolar proton pump [Danio rerio]	1.8
C609954.1	Autocrine motility factor receptor [T. castaneum]	1.5
C608609.1	Voltage-dependent anion channel 1 [X. laevis]	1.6
C601007.1	UDP dual transporter [C. elegans]	2.0
C609782.1	DAD-1-like protein/integral membrane [S. japonicum]	1.9
Receptors interacting proteins		
C610202.1	Serine/threonine kinase receptor associated protein [P. troglodytes]	1.6
C607204.1	Thyroid receptor interacting protein 13 [H. sapiens]	1.7
C603157.1	syndecan binding protein (syntenin) [D. rerio]	1.5
C606116.1	G protein beta subunit [P. fucata]	2.1
C600861.1	B-cell receptor-associated protein-like protein [S. mansoni]	1.5
C608810.1	rhoGAP protein [M. domestica]	2.7
C714516.1	TGF-beta receptor interacting protein 1 [C. sinensis]	1.8
Lipid metabolism		
C601385.1	SmINSIG [S. mansoni]	2.3
C601665.1	Fatty acid-binding protein Sm14 [S. mansoni]	1.5
C608771.1	Very low density lipoprotein binding protein [S. japonicum]	1.8
C711918.1	Fatty acid coenzyme A ligase 5 [H. sapiens]	1.7
Other		
C602958.1	Apoferritin-2 [S. japonicum]	1.7
C610848.1	Ferritin heavy chain 2 [S. mansoni]	2.1
C601146.1	IB1 protein [S. japonicum]	2.9
C608351.1	Tropomyosin [S. mansoni]	1.7
C600304.1	src tyrosine kinase [S. mansoni]	1.7
C603876.1	PrA2 protein [S. mansoni]	1.7
C607402.1	Superoxide dismutase [S. mansoni]	1.5
C604746.1	Sm65 antigen [S. mansoni]	1.5

a statistically significant, q<0.01

## Discussion

Schistosomiasis is one of the most important neglected tropical diseases (NTDs), and an effective control is unlikely in the absence of improved sanitation and a vaccine. Recently, the tegument proteome of *S. mansoni* was characterized, and one study in particular revealed the major proteins that are exposed on live adult parasites such as SmTSP-2 and Sm29 [11]. In this study, we investigated murine humoral and cellular immune responses to rSm29 and tested this molecule as a vaccine candidate.

The strategic localization of Sm29 in the mammalian skin-stage and lung-stage schistosomula and its absence in cercariae suggests that this antigen has an important role in the adaptation of the parasite to the new environment when *S. mansoni* enters the mammalian host. Previous studies using mice vaccinated with irradiated cercariae showed that the lung is the key site of elimination of this parasite [24]. Sm29 localization on the surface of schistosomula is an important aspect of this antigen regarding its protective properties.

In the mouse model, rSm29 induced high levels of anti-Sm29 IgG after the second immunization and showed reduced IgG1/IgG2a ratio at 45 days after the first immunization (Table 1). This finding suggests a tendency of a Th1 type of immune response induced by rSm29 vaccination. Further, we confirmed by cytokine analysis that rSm29 immunization elicited a Th1-type of immune response characterized by high levels of IFN-γ and no IL-4 and 51% of worm burden reduction. The involvement of IFN-γ in protective immunity to schistosomiasis is well documented in the murine model [25]. In the irradiated cercariae vaccination model, which induces high levels of protection, treatment with monoclonal anti-IFN-γ antibody totally abrogated the protective immunity achieved [26]. Similar results were obtained using IFN-γ knockout mice exposed to the radiation-attenuated vaccine confirming the essential role of IFN-γ in protective immunity against murine schistosomiasis [27]. Further, we tested the ability of rSm29 to induce IL-12 in peritoneal macrophages of TLR4 KO mice. TLR4 is the major receptor for macrophage activation by bacterial LPS [28]. Therefore, using TLR4 KO we obviated any possible effect of LPS contamination in IL-12 synthesis. In TLR4 KO mice, rSm29 has induced the production of large amounts of IL-12 that is the key cytokine involved in Th1 cell development.

Pathology which results from granuloma formation around the eggs in murine schistosomiasis is characterized by Th2-type of immune response and the granuloma size can be reduced by neutralization of IL-4 [29]. Thus, morbidity and mortality in murine schistosomiasis were hypothesized to be developed as a direct consequence of the egg-induced Th2 type of immune response. Herein, the intestinal egg numbers was reduced (60%) following rSm29 vaccination compared to control group. Additionally, the immune response elicited by Sm29 was associated with 50% reduction in granuloma counts. Since we detected IL-10 production following splenocyte activation with rSm19, we hypothesize here that this cytokine might be regulating Th2 responses and/or preventing the development of highly polarized Th1 responses and therefore, reducing inflammation and liver pathology [30],[31].

In order to investigate the impact of rSm29 vaccination on *S. mansoni*, worms that were recovered from vaccinated mice had their gene expression profile analyzed using microarrays. In worms recovered from mice vaccinated with adjuvanted rSm29 when compared to a control group injected with adjuvanted phosphate-buffered saline, 495 genes (96%) were down-regulated and only 22 genes (4%) were up-regulated. Among down-regulated genes, many of them encode surface antigens and proteins associated with immune signals suggesting that under immune attack schistosomes reduce the expression of critical surface proteins. We propose here that down-regulation of expression of important surface antigens is an important strategy of the parasite resulting in escape from host immune surveillance.

As observed in Table 3, Sm23, tetraspanin TE736 (a paralog of Sm-TSP-2), Sm14, Sm65 and superoxide dismutase are significantly (q<0.01) down-regulated in adult-worms recovered from rSm29 protected mice; it is noteworthy that these genes encode important schistosome antigens that are recognized by immune mice and humans. Sm14 is able to induce cellular immune responses in individuals from endemic areas for schistosomiasis [32]. Sm23 and Sm65 are antigens recognized by sera from schistosome infected patients [33],[34]. Moreover, Sm14 and Sm23 induce partial protection in the murine model for schistosomiasis [35],[36]. Regarding tetraspanins, they belong to a family of membrane proteins, such as Sm-TSP-2, that is currently one of the most important vaccine candidates for schistosomiasis [37]. Further, vaccination of mice with naked DNA construct containing Cu/Zn cytosolic superoxide dismutase showed significant levels of protection compared to a control group [38].

It is noteworthy that no significant up-regulation of heat shock or chaperone genes was detected in schistosomes recovered from rSm29 vaccinated mice (Table S1), therefore excluding a common stress response. Rather, lowering the expression of critical surface antigens seems to be an important and specific strategy developed by schistosomes to reduce the damage caused by the host immune system. Therefore, a combined antigen vaccination with Sm29 and one or more of the proteins encoded by these down-regulated critical surface antigens might be the next logical step, thus circumventing the adaptive response of the parasite and eventually boosting the protective effect of vaccination.

Among the down-regulated genes (Table 3) were those encoding protein domains from TGF-β receptor interacting protein, B-cell receptor-associated protein, CD36-like class B scavenger receptor, all of which are associated with host immune response. These parasite gene products could interact with or modulate the host immune response. For example, the scavenger receptors are present on different cells such as macrophages, platelets and neutrophils where they are involved with innate immunity by facilitating phagocytosis, cell adhesion and pathogen recognition. Recently, it was demonstrated that schistosome CD36-like class B scavenger receptor binds to host low density lipoproteins [39]. From the parasite perspective, it would not be difficult to envisage that a reduced expression of *S. mansoni* scavenger receptor-like molecules would decrease pathogen recognition and cell adhesion by host cells.

Additionally, we found down-regulated genes involved with parasite carbohydrate and lipid metabolism, protein biosynthesis, intracellular signaling cascade, among others (Table S1). Down-regulated genes include SmINSIG (an ortholog of the mammal Insulin Induced Gene), a recently described gene in *S. mansoni* [40] that has a key role in HMG-CoA reductase degradation. HMG-CoA reductase is vital for egg production by *S. mansoni* [41]. An alternative hypothesis to our findings is that down-regulation of these genes may affect the development or survival of parasites in vaccinated animals, thus reducing worm burden, although the worms recovered from mice vaccinated with adjuvanted rSm29 are morphologically similar to those recovered from control mice. Additionally, haemoglobinases and Sm29 gene expression were unaltered in worms recovered from rSm29 immunized animals. Recently, Dillon et al [42] in an attempt to identify antigens responsible for protection induced by irradiated-cercariae vaccination performed microarray expression analysis of irradiated and normal worms. These investigators detected down-regulation of genes involved in neuromuscular activity and cell cycle. Their hypothesis is that attenuated parasite might have an extended stay in the host enhancing immune priming against exposed antigens [42]. Their findings may explain why irradiated-cercariae can elicit protective immunity when normal cercariae do not.

Recent advances in antigen discovery with preclinical studies showing promising efficacy have reinvigorated the case for a schistosomiasis vaccine. The irradiated-cercariae vaccine and the use of recombinant antigens, such as Sm-TSP-2 and Sm29, that induce approximately 50% worm burden reduction, have demonstrated that a vaccine to schistosomes is achievable [37]. Furthermore, we identified an ortholog of Sm29 in the ESTs of the Asian schistosome *S. japonicum* that shares more than 50% identity in the amino acid sequence, indicating that a vaccine based on Sm29 might also be effective against *S. japonicum* [43]. Another important issue is the selection of a suitable adjuvant and/or delivery system to induce the appropriate immune responses. Herein, we used rSm29 with Freund́s adjuvant, however, it is not suitable for human application. Experiments are underway testing rSm29 with CpG or CpG plus alum as suggested by McManus & Loukas [44]. As a final conclusion of this work, we believe that Sm29 is an efficient vaccine candidate against schistosomiasis in light of the data obtained from murine studies. The best long-term strategy to control schistosomiasis might be immunization with anti-Sm29 vaccine associated with other protective surface antigens and possibly combined with drug treatment.

## Supporting Information

Figure S1 Immunolocaliztion of Sm29 on cercariae by confocal microscopy
(0.02 MB PDF)

Table S1 Complete list of all genes and fold-changes determined by microarray analysis
(0.15 MB PDF)

Table S2 List of primers used for validation by real-time RT-PCR
(0.04 MB PDF)

Video S1 Movie of confocal microscopy images. Immunolocalization of Sm29 on the tegument of male adult worm. File is in AVI format.
(7.04 MB ZIP)
