# Supplementary figures and images for: Schistosoma mansoni Tegument Protein Sm29 Is Able to Induce a Th1-Type of Immune Response and Protection against Parasite Infection
Source: PLoS Negl Trop Dis. 2008 Oct 1;2(10):e308. doi: 10.1371/journal.pntd.0000308 (PMC2553283; doi:10.1371/journal.pntd.0000308)

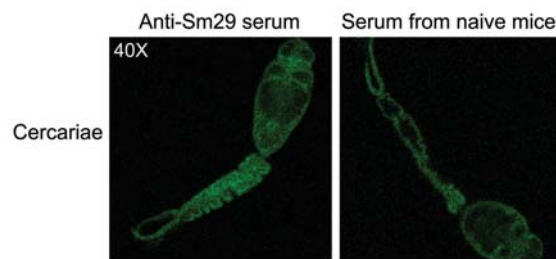

Supplement: Figure S1 — Immunolocaliztion of Sm29 on cercariae by confocal microscopy (0.02 MB PDF) [file pntd.0000308.s001.pdf]
