# Supplementary material for: Schistosoma mansoni Tegument Protein Sm29 Is Able to Induce a Th1-Type of Immune Response and Protection against Parasite Infection
Source: PLoS Negl Trop Dis. 2008 Oct 1;2(10):e308. doi: 10.1371/journal.pntd.0000308 (PMC2553283; doi:10.1371/journal.pntd.0000308)
Supplement: Table S1 — Complete list of all genes and fold-changes determined by microarray analysis (0.15 MB PDF) [file pntd.0000308.s002.pdf]

rank #	Contig	Putative identity (annotation based on the five best hits by BLAST search in GenBank)	log2 (test/control); Average 90_92(L)	log2 (test/control); Average 90_92(R)	log2 (test/control); Average 95_91(L)	log2 (test/control); Average 95_91(R)	Median log2 (test/control)	Fold Change*	up- or down-regulation**
1	C601646.1	gb|AAW24491.1||unknown [Schistosoma japonicum]; gb|AAW27052.1||unknown [Schistosoma japonicum]; gb|AAP05892.1||hypothetical protein [Schistosoma japonicum]; gb|ACE06850.1||unknown [Schistosoma japonicum];	-1,911635991	-1,916860881	-2,539131732	-2,483400315	-2,200130598	4,6	DOWN
2	C609462.1	gb|AAW27174.1||SJCHGC06407 protein [Schistosoma japonicum]; gb|AAW25739.1||SJCHGC02535 protein [Schistosoma japonicum]; gb|AAW27568.1||SJCHGC02279 protein [Schistosoma japonicum]; gb|ACE06927.1||unknown [Schistosoma japonicum];	-1,688135128	-1,796641657	-1,76949007	-1,664122819	-1,728812599	3,3	DOWN
3	C612169.1	No match	-1,464504037	-1,437284429	-1,957083948	-1,972765517	-1,710793993	3,3	DOWN
4	C608060.1	emb|CAG02932.1||unnamed protein product [Tetraodon nigroviridis]; gb|AAI16544.1||Thoc2 protein [Danio rerio]; ref|NP_001003847.1||Tho2 [Danio rerio] gb|AAT68136.1| Tho2 [Danio rerio]; gb|AAH93105.1||Thoc2 protein [Danio rerio];	-1,872416457	-1,896338176	-1,420751516	-1,307612643	-1,646583987	3,1	DOWN
5	C601379.1	No match	-1,89162378	-1,933031415	-1,367269277	-0,883171054	-1,629446529	3,1	DOWN
6	C607243.1	gb|ABW08111.1||alkaline phosphatase [Schistosoma mansoni]; ref|XP_606676.3||PREDICTED: similar to intestinal alkaline phosphatase III; IAP III [Bos taurus]; gb|AAG39908.1||tissue-nonspecific alkaline phosphatase [Mus musculus]; sp|P08289|PPBT_RAT|Alkaline phosphatase, tissue-nonspecific isozyme precursor (AP-TNAP) (TNSALP) (Alkaline phosphatase liver/bone/kidney isozyme) emb|CAA34160.1| alkaline phosphatase [Rattus norvegicus] gb|AAH88399.1| Alkaline phosphatase, liver/bone/kidney [Rattus norvegicus] gb|EDL80848.1| alkaline phosphatase, tissue-nonspecific, isoform CRA_a [Rattus norvegicus] gb|EDL80849.1| alkaline phosphatase, tissue-nonspecific, isoform CRA_a [Rattus norvegicus] gb|EDL80850.1| alkaline phosphatase, tissue-nonspecific, isoform CRA_a [Rattus norvegicus];	-1,234545141	-1,192710413	-2,028417714	-1,999880152	-1,617212647	3,1	DOWN
7	C601146.1	gb|AAW24964.1||SJCHGC03760 protein [Schistosoma japonicum]; gb|AAW24762.1||SJCHGC04453 protein [Schistosoma japonicum]; gb|AAW26349.1||SJCHGC03072 protein [Schistosoma japonicum]; gb|AAM53938.1|AF514300_1|IB1 protein [Schistosoma japonicum];	-1,341485213	-1,408984609	-1,689633984	-1,627427217	-1,518205913	2,9	DOWN
8	C606709.1	gb|AAX28327.2||SJCHGC05589 protein [Schistosoma japonicum]; ref|XP_001175610.1||PREDICTED: similar to Synaptophysin (Major synaptic vesicle protein p38) [Strongylocentrotus purpuratus]; ref|NP_989081.1||synaptophysin-like 1 [Xenopus tropicalis] gb|AAH62512.1| Synaptophysin-like 1 [Xenopus tropicalis] emb|CAJ81619.1| synaptophysin-like 1 [Xenopus tropicalis]; gb|EDV29003.1||hypothetical protein TRIADDRAFT_52445 [Trichoplax adhaerens];	-1,261818992	-1,372273074	-1,644519908	-1,741013975	-1,508396491	2,8	DOWN
9	C611037.1	gb|AAW27737.1||SJCHGC04773 protein [Schistosoma japonicum];	-1,50033629	-1,563610982	-1,434024306	-1,46215157	-1,48124393	2,8	DOWN
10	C609059.1	tpe|CAJ00234.1||TPA: hypothetical protein [Schistosoma mansoni];	-1,322380687	-1,473296649	-1,461626265	-1,438219799	-1,449923032	2,7	DOWN

11	C608810.1	ref|XP_001369707.1||PREDICTED: similar to rhoGAP protein [Monodelphis domestica]; gb|EDL04450.1||mCG141063, isoform CRA_b [Mus musculus]; gb|AAH10306.2||3110043J09Rik protein [Mus musculus]; sp|Q9CXP4.3|RHG08_MOUSE|Rho GTPase-activating protein 8 (Rho-type GTPase-activating protein 8) gb|AAH05563.1| RIKEN cDNA 3110043J09 gene [Mus musculus] dbj|BAC39750.1| unnamed protein product [Mus musculus];	-1,625317595	-1,658782319	-1,264853485	-1,243484259	-1,44508554	2,7	DOWN
12	C707839.1	ref|XP_001110803.1||PREDICTED: HtrA serine peptidase 2 isoform 2 [Macaca mulatta]; dbj|BAF84666.1||unnamed protein product [Homo sapiens]; ref|XP_001500096.1||PREDICTED: similar to HtrA serine peptidase 2 isoform 1 [Equus caballus]; ref|XP_001500119.1||PREDICTED: hypothetical protein isoform 2 [Equus caballus];	-1,412230832	-1,443759851	-1,468326964	-1,354259715	-1,427995342	2,7	DOWN
13	C603200.1	No match	-1,206112282	-1,422392002	-1,386728941	-1,468875708	-1,404560472	2,6	DOWN
14	C612148.1	gb|EDW79485.1||GK20386 [Drosophila willistoni]; gb|EDW18112.1||GI13050 [Drosophila mojavensis]; gb|EDV39506.1||GF24451 [Drosophila ananassae]; gb|EDW33061.1||GL16175 [Drosophila persimilis];	-1,41005322	-1,557144886	-1,394107429	-1,334247789	-1,402080325	2,6	DOWN
15	C609450.1	ref|XP_580486.2||PREDICTED: similar to DNA cross-link repair 1A (PSO2 homolog, S. cerevisiae) [Bos taurus]; ref|XP_001926894.1||PREDICTED: similar to DNA cross-link repair 1A (PSO2 homolog, S. cerevisiae) [Sus scrofa]; ref|NP_061301.2||DNA cross-link repair 1A, PSO2 homolog [Mus musculus] gb|EDL01762.1| DNA cross-link repair 1A, PSO2 homolog (S. cerevisiae), isoform CRA_b [Mus musculus]; dbj|BAD90139.1||mKIAA0086 protein [Mus musculus];	-1,378589405	-1,406377436	-1,735158412	-1,218460932	-1,392483421	2,6	DOWN
16	C600211.1	No match	-1,362917849	-1,4854718	-1,421016031	-1,152952641	-1,39196694	2,6	DOWN
17	C604312.1	gb|AAW25986.1||SJCHGC02396 protein [Schistosoma japonicum]; gb|AAP06073.1||similar to NM_022756 hypothetical protein FLJ11730 in Homo sapiens; hypothetical protein FLJ11730 in Homo sapiens [Schistosoma japonicum]; gb|AAW25871.1||SJCHGC00146 protein [Schistosoma japonicum]; gb|AAW25057.1||unknown [Schistosoma japonicum];	-1,45929147	-1,352518024	-1,398289729	-1,039995326	-1,375403877	2,6	DOWN
18	C600324.1	No match	-1,536961606	-1,566819379	-1,094790921	-1,196275042	-1,366618324	2,6	DOWN
19	C609525.1	gb|EDV23243.1||hypothetical protein TRIADDRAFT_27759 [Trichoplax adhaerens]; ref|NP_001007877.1||MGC89709 protein [Xenopus tropicalis] sp|Q68EQ9|TMM49_XENTR Transmembrane protein 49 gb|AAH80142.1| MGC89709 protein [Xenopus tropicalis]; ref|XP_415880.1||PREDICTED: hypothetical protein [Gallus gallus]; ref|NP_996943.1||transmembrane protein 49 [Danio rerio] sp|Q6NYY9|TMM49_BRARE Transmembrane protein 49 gb|AAH66412.1| Transmembrane protein 49 [Danio rerio];	-1,235777614	-1,273833154	-1,429465759	-1,480930785	-1,351649457	2,6	DOWN
20	C602031.1	gb|AAW25516.1||SJCHGC02507 protein [Schistosoma japonicum]; ref|XP_001504303.1||PREDICTED: similar to golgi SNAP receptor complex member 1 isoform 1 [Equus caballus]; ref|XP_001504305.1||PREDICTED: similar to golgi SNAP receptor complex member 1 isoform 2 [Equus caballus]; ref|NP_001006222.1||golgi SNAP receptor complex member 1 [Gallus gallus] emb|CAG31892.1| hypothetical protein [Gallus gallus];	-1,176546662	-1,234038948	-1,393808984	-1,435480802	-1,313923966	2,5	DOWN

21	C606451.1	No match	-1,068172875	-1,307823795	-1,37421799	-1,317624708	-1,312724252	2,5	DOWN
22	C600569.1	gb|AAW25266.1||SJCHGC09427 protein [Schistosoma japonicum]; ref|XP_001950428.1||PREDICTED: similar to Splicing factor 3A subunit 3 (Spliceosome-associated protein 61) (SAP 61) (SF3a60) [Acyrthosiphon pisum]; ref|XP_001606271.1||PREDICTED: hypothetical protein [Nasonia vitripennis]; ref|XP_785281.2||PREDICTED: hypothetical protein, partial [Strongylocentrotus purpuratus] ref|XP_001182994.1| PREDICTED: hypothetical protein, partial [Strongylocentrotus purpuratus];	-1,113628406	-1,149337064	-1,574393487	-1,388075559	-1,268706312	2,4	DOWN
23	C600192.1	ref|XP_419506.2||PREDICTED: similar to ATP-binding cassette, sub-family C, member 10 [Gallus gallus]; ref|XP_585169.3||PREDICTED: similar to FLJ00002 protein [Bos taurus]; dbj|BAA92227.1||FLJ00002 protein [Homo sapiens]; sp|Q5T3U5|MRP7_HUMAN|Multidrug resistance-associated protein 7 (ATP-binding cassette sub-family C member 10) emb|CAI23217.1| ATP-binding cassette, sub-family C (CFTR/MRP), member 10 [Homo sapiens] gb|EAX04181.1| ATP-binding cassette, sub-family C (CFTR/MRP), member 10, isoform CRA_b [Homo sapiens] gb|EAX04182.1| ATP-binding cassette, sub-family C (CFTR/MRP), member 10, isoform CRA_b [Homo sapiens] dbj|BAG10267.1| ATP-binding cassette, sub-family C member 10 [synthetic construct];	-1,337450303	-1,336983978	-1,190939057	-1,079621314	-1,263961518	2,4	DOWN
24	C606289.1	No match	-1,877981365	-0,37944501	-1,23903302	-1,288679617	-1,263856319	2,4	DOWN
25	C601991.1	gb|AAW26256.1||SJCHGC06423 protein [Schistosoma japonicum]; ref|XP_001635718.1||predicted protein [Nematostella vectensis] gb|EDO43655.1| predicted protein [Nematostella vectensis]; ref|NP_001016075.1||ORM1-like 1 [Xenopus tropicalis]; emb|CAG03979.1||unnamed protein product [Tetraodon nigroviridis];	-0,839263785	-0,894380521	-1,622558713	-1,599613379	-1,24699695	2,4	DOWN
26	C607394.1	gb|AAX24553.2||SJCHGC06523 protein [Schistosoma japonicum];	-1,283216941	-1,345699685	-1,177916928	-1,207814791	-1,245515866	2,4	DOWN
27	C602913.1	gb|AAW25145.1||SJCHGC02192 protein [Schistosoma japonicum]; ref|XP_001604182.1||PREDICTED: similar to conserved hypothetical protein [Nasonia vitripennis]; ref|XP_967379.1||PREDICTED: similar to CG11964 CG11964-PA [Tribolium castaneum]; gb|EDX13697.1||GD20833 [Drosophila simulans];	-0,925637164	-1,045381276	-1,503341567	-1,442646034	-1,244013655	2,4	DOWN
28	C604210.1	No match	-1,152875767	-1,121537306	-1,388943686	-1,30846746	-1,230671614	2,3	DOWN
29	C601385.1	emb|CAM34681.1||INSIG protein [Schistosoma mansoni]; ref|NP_956163.1||insulin induced gene 1 [Danio rerio] ref|XP_001922160.1| PREDICTED: similar to insulin induced gene 1 [Danio rerio] sp|Q8AV61|INSI1_BRARE Insulin-induced gene 1 protein (INSIG-1) gb|AAN28327.1| INSIG-1 membrane protein [Danio rerio] gb|AAH45341.1| Insulin induced gene 1 [Danio rerio] gb|AAI65115.1| Insig1 protein [synthetic construct]; ref|XP_001249364.1||PREDICTED: hypothetical protein LOC511899 isoform 1 [Bos taurus]; ref|NP_001091219.1||hypothetical protein LOC100036994 [Xenopus laevis] gb|AAI30075.1| LOC100036994 protein [Xenopus laevis];	-1,408537827	-1,287211021	-1,168369095	-0,871718322	-1,227790058	2,3	DOWN
30	C608987.1	No match	-1,432652068	-1,427696738	-1,018936296	-0,946580762	-1,223316517	2,3	DOWN

31	C602990.1	gb|AAW27619.1||SJCHGC06261 protein [Schistosoma japonicum]; ref|NP_505261.2||B0507.2 [Caenorhabditis elegans] sp|Q22885.2|BROX_CAEEL BRO1 domain-containing protein BROX homolog gb|AAB04821.2| Hypothetical protein B0507.2 [Caenorhabditis elegans]; ref|XP_419397.1||PREDICTED: similar to RIKEN cDNA 0610010K06 gene [Gallus gallus]; emb|CAG12984.1||unnamed protein product [Tetraodon nigroviridis];	-1,199861305	-1,295025785	-1,166821143	-1,243795651	-1,221828478	2,3	DOWN
32	C607921.1	ref|XP_969961.1||PREDICTED: similar to syntaxin 18 [Tribolium castaneum]; ref|NP_001019596.1||hypothetical protein LOC554130 [Danio rerio] sp|Q4VBI7|STX18_BRARE Syntaxin-18 gb|AAH95763.1| Zgc:112326 [Danio rerio]; gb|AAH73006.1||LOC443606 protein [Xenopus laevis]; gb|AAH92152.1||Unknown (protein for IMAGE:6949850) [Xenopus laevis];	-1,447576779	-1,425206619	-0,999483108	-0,731493256	-1,212344864	2,3	DOWN
33	C715533.1	gb|AAX24320.2||SJCHGC03143 protein [Schistosoma japonicum]; gb|EDL05030.1||autophagy-related 5 (yeast) [Mus musculus]; ref|NP_444299.1||autophagy-related 5-like [Mus musculus] sp|Q99J83|ATG5_MOUSE Autophagy protein 5 (APG5-like) gb|AAH02166.1| Autophagy-related 5 (yeast) [Mus musculus] dbj|BAB33383.1| mApg5 [Mus musculus] dbj|BAC25874.1| unnamed protein product [Mus musculus]; ref|XP_001503989.1||PREDICTED: similar to Autophagy protein 5 (APG5-like) (Apoptosis-specific protein) isoform 2 [Equus caballus] ref|XP_001503988.1| PREDICTED: similar to Autophagy protein 5 (APG5-like) (Apoptosis-specific protein) isoform 1 [Equus caballus];	-1,03479893	-1,150219358	-1,242173615	-1,307037386	-1,196196487	2,3	DOWN
34	C611698.1	gb|EDL24096.1||eukaryotic translation initiation factor 3, subunit 12, isoform CRA_a [Mus musculus]; gb|AAH89069.1||Eif3s12-prov protein [Xenopus tropicalis]; emb|CAJ83157.1||eukaryotic translation initiation factor 3, subunit 12 [Xenopus tropicalis] gb|AAI57543.1| Unknown (protein for MGC:180700) [Xenopus tropicalis]; ref|NP_082935.1||eukaryotic translation initiation factor 3, subunit K [Mus musculus] sp|Q9DBZ5.1|EIF3K_MOUSE Eukaryotic translation initiation factor 3 subunit K (eIF3k) (Eukaryotic translation initiation factor 3 subunit 12) (eIF-3 p25) dbj|BAB23454.1| unnamed protein product [Mus musculus] gb|AAH91749.1| Eukaryotic translation initiation factor 3, subunit K [Mus musculus];	-1,041671509	-1,151302084	-1,300764075	-1,234192867	-1,192747476	2,3	DOWN
35	C602061.1	No match	-1,427963928	-1,360236587	-0,99528509	-0,777538931	-1,177760839	2,3	DOWN

36	C606486.1	sp|Q9UHV7.2|MED13_HUMAN|Mediator of RNA polymerase II transcription subunit 13 (Mediator complex subunit 13) (Thyroid hormone receptor-associated protein complex 240 kDa component) (Trap240) (Thyroid hormone receptor-associated protein 1) (Vitamin D3 receptor-interacting protein complex component DRIP250) (DRIP 250) (Activator-recruited cofactor 250 kDa component) (ARC250); ref|NP_005112.2||mediator complex subunit 13 [Homo sapiens] gb|EAW51438.1| thyroid hormone receptor associated protein 1, isoform CRA_b [Homo sapiens] gb|EAW51439.1| thyroid hormone receptor associated protein 1, isoform CRA_b [Homo sapiens] gb|AAI40892.1| Mediator complex subunit 13 [Homo sapiens]; dbj|BAA25519.2||KIAA0593 protein [Homo sapiens]; gb|AAD22032.1||thyroid hormone receptor-associated protein complex component TRAP240 [Homo sapiens];	-1,093591134	-0,988696567	-1,25809344	-1,240242489	-1,166916812	2,2	DOWN
37	C604197.1	gb|AAW26476.1||SJCHGC01111 protein [Schistosoma japonicum]; ref|XP_001638494.1||predicted protein [Nematostella vectensis] gb|EDO46431.1| predicted protein [Nematostella vectensis]; gb|AAW25407.1||SJCHGC02502 protein [Schistosoma japonicum]; gb|EDW49160.1||GM23254 [Drosophila sechellia];	-1,179856567	-1,14651979	-1,260115199	-1,129145371	-1,163188179	2,2	DOWN
38	C603237.1	gb|EDV52205.1||GG15951 [Drosophila erecta]; ref|XP_392451.3||PREDICTED: similar to Transcription initiation factor TFIID subunit 4 (Transcription initiation factor TFIID 110 kDa subunit) (p110) (TAFII-110) (110 kDa TBP-associated factor) [Apis mellifera]; gb|EDV40044.1||GF24156 [Drosophila ananassae]; ref|XP_966532.1||PREDICTED: similar to transcription initiation factor [Tribolium castaneum];	-1,195153819	-1,200985984	-1,122228905	-0,910708589	-1,158691362	2,2	DOWN
39	C611641.1	ref|NP_476908.1||Vacuolar H[+]-ATPase 55kD B subunit CG17369-PB, isoform B [Drosophila melanogaster] ref|NP_731726.1| Vacuolar H[+]-ATPase 55kD B subunit CG17369-PA, isoform A [Drosophila melanogaster] sp|P31409|VATB_DROME Vacuolar ATP synthase subunit B (V-ATPase subunit B) (Vacuolar proton pump subunit B) (V-ATPase 55 kDa subunit) emb|CAA48034.1| vacuolar ATPase B subunit [Drosophila melanogaster] gb|AAF54836.1| CG17369-PA, isoform A [Drosophila melanogaster] gb|AAF54837.1| CG17369-PB, isoform B [Drosophila melanogaster] gb|AAK93047.1| GH27148p [Drosophila melanogaster] gb|AAN71057.1| AT12604p [Drosophila melanogaster] gb|EDV30327.1| GF23001 [Drosophila ananassae] gb|EDV49337.1| GG17111 [Drosophila erecta] gb|EDW15311.1| GI24752 [Drosophila mojavensis] gb|EDW83646.1| GK13566 [Drosophila willistoni] gb|EDW97128.1| GE24501 [Drosophila yakuba]; ref|XP_001357789.1||GA14484-PA [Drosophila pseudoobscura] gb|EAL26924.1| GA14484-PA [Drosophila pseudoobscura] gb|EDW39130.1| GL13570 [Drosophila persimilis]; gb|EDW42428.1||GM25995 [Drosophila sechellia] gb|EDX13185.1| GD20555 [Drosophila simulans]; sp|P31401|VATB	-1,256884225	-1,21431755	-1,004946098	-1,098672701	-1,156495126	2,2	DOWN
40	C608241.1	gb|AAW24763.1||SJCHGC05980 protein [Schistosoma japonicum]; gb|AAP06091.1||hypothetical protein [Schistosoma japonicum];	-0,851798823	-0,94544227	-1,36265391	-1,521643952	-1,15404809	2,2	DOWN
41	C603939.1	No match	-1,152120674	-1,152588876	-1,147362555	-0,932952437	-1,149741615	2,2	DOWN

42	C611360.1	gb|AAX27020.1||unknown [Schistosoma japonicum]; gb|AAX27010.1||unknown [Schistosoma japonicum] gb|AAX30131.1| SJCHGC00333 protein [Schistosoma japonicum]; gb|AAX27546.2||SJCHGC02390 protein [Schistosoma japonicum];	-0,891469404	-1,016190071	-1,316108353	-1,279418056	-1,147804064	2,2	DOWN
43	C600203.1	gb|AAX24848.2||SJCHGC09620 protein [Schistosoma japonicum];	-1,135086038	-1,054739593	-1,197689191	-1,141656171	-1,138371105	2,2	DOWN
44	C605209.1	gb|AAO59420.2||lactate dehydrogenase-like protein [Schistosoma japonicum]; gb|AAW25909.1||SJCHGC02274 protein [Schistosoma japonicum]; gb|AAP06144.1||similar to GenBank Accession Number AF070998 lactate dehydrogenase A in Ambystoma mexicanum [Schistosoma japonicum]; gb|AAV80238.1||lactate dehydrogenase [Clonorchis sinensis];	-1,030978064	-1,106018587	-1,17181833	-1,149102922	-1,127560755	2,2	DOWN
45	C606417.1	gb|AAW25054.1||SJCHGC05912 protein [Schistosoma japonicum]; gb|AAP06438.1||similar to NM_005731 actin related protein 2/3 complex, subunit 2 (34 kD) in Homo sapiens [Schistosoma japonicum]; ref|XP_001915183.1||PREDICTED: similar to actin related protein 2/3 complex subunit 2 [Equus caballus]; ref|XP_001925034.1||PREDICTED: similar to actin related protein 2/3 complex subunit 2 [Sus scrofa];	-1,051114724	0,290335283	-1,286142675	-1,201478285	-1,126296505	2,2	DOWN
46	C609140.1	gb|AAW26075.1||SJCHGC05150 protein [Schistosoma japonicum];	-1,038911567	-0,93138094	-1,275095676	-1,19562424	-1,117267904	2,2	DOWN
47	C604803.1	gb|AAW26542.1||SJCHGC05854 protein [Schistosoma japonicum];	-0,852963283	-0,930573288	-1,423037902	-1,296941893	-1,113757591	2,2	DOWN
48	C604360.1	gb|AAW27458.1||SJCHGC06870 protein [Schistosoma japonicum]; ref|XP_973073.1||PREDICTED: similar to putative GABA-A receptor associated protein [Tribolium castaneum]; gb|ABD98773.1||putative GABA-A receptor associated protein [Graphocephala atropunctata]; ref|XP_001844428.1||conserved hypothetical protein [Culex pipiens quinquefasciatus] gb|EDS36925.1| conserved hypothetical protein [Culex quinquefasciatus];	-1,126630311	-1,295304674	-1,100214006	-0,87694941	-1,113422159	2,2	DOWN
49	C610331.1	No match	-1,034379174	-1,008598461	-1,186859453	-1,236342126	-1,110619314	2,2	DOWN
50	C608397.1	No match	-1,004613078	-0,719512903	-1,210204951	-1,232828269	-1,107409015	2,2	DOWN
51	C611326.1	tpe|CAJ00239.1||TPA: hypothetical protein [Schistosoma mansoni]; tpe|CAJ00241.1||TPA: hypothetical protein [Schistosoma mansoni]; tpe|CAJ00234.1||TPA: hypothetical protein [Schistosoma mansoni];	-1,063129506	-1,137064824	-1,202929945	-0,93871013	-1,100097165	2,1	DOWN
52	C610848.1	sp|P25320|FRIH2_SCHMA|Ferritin-2 heavy chain gb|AAA29881.1| ferritin light chain; gb|AAW26332.1||SJCHGC06812 protein [Schistosoma japonicum]; gb|AAS92978.1||ferritin [Clonorchis sinensis]; gb|ABD75379.1||ferritin H [Bufo gargarizans];	-0,390065036	-0,565615937	-1,631665709	-1,785583211	-1,098640823	2,1	DOWN
53	C611493.1	No match	-0,850715805	-1,018348902	-1,202836749	-1,147263888	-1,082806395	2,1	DOWN
54	C604310.1	No match	-1,157134047	-1,164018133	-0,997781542	-0,925962871	-1,077457795	2,1	DOWN
55	C708663.1	gb|AAW24733.1||SJCHGC06204 protein [Schistosoma japonicum]; gb|EDW94314.1||GE20110 [Drosophila yakuba]; gb|EDW78772.1||GK12617 [Drosophila willistoni]; gb|EDW70509.1||GJ13811 [Drosophila virilis];	-1,529115581	-1,378682528	-0,769698248	-0,672076028	-1,074190388	2,1	DOWN
56	C610760.1	No match	-0,826926921	-0,95540418	-1,192191891	-1,248999443	-1,073798036	2,1	DOWN

57	C606116.1	gb|AAW26990.1||SJCHGC05537 protein [Schistosoma japonicum]; gb|AAS90835.1||G protein beta subunit [Pinctada fucata]; sp|P23232|GBB_LOLFO|Guanine nucleotide-binding protein subunit beta emb|CAA40077.1| GTP-binding protein beta subunit [Loligo forbesi] prf||1705183A GTP binding protein; gb|AAQ90156.1||visual G-protein beta subunit [Loligo pealei];	-0,789967769	-1,264298677	-1,050198002	-1,088311455	-1,069254729	2,1	DOWN
58	C606703.1	gb|AAW26108.1||SJCHGC05178 protein [Schistosoma japonicum]; ref|NP_082588.1||coiled-coil domain containing 12 [Mus musculus] dbj|BAB28353.1| unnamed protein product [Mus musculus]; sp|Q8R344|CCD12_MOUSE|Coiled-coil domain-containing protein 12 gb|EDL09006.1| coiled-coil domain containing 12 [Mus musculus]; ref|NP_001102253.1||coiled-coil domain containing 12 [Rattus norvegicus] gb|EDL77064.1| coiled-coil domain containing 12 (predicted), isoform CRA_a [Rattus norvegicus];	-0,987741383	-0,998254939	-1,158129123	-1,137015205	-1,067635072	2,1	DOWN
59	C604859.1	gb|AAW25677.1||SJCHGC01656 protein [Schistosoma japonicum]; ref|XP_001896774.1||UPF0279 protein C14orf129 homolog [Brugia malayi] gb|EDP34381.1| UPF0279 protein C14orf129 homolog, putative [Brugia malayi]; ref|XP_001367855.1||PREDICTED: similar to Chromosome 14 open reading frame 129 [Monodelphis domestica]; gb|EDV41299.1||GF23488 [Drosophila ananassae];	-0,616091889	-0,664292342	-1,466499461	-1,508303365	-1,065395902	2,1	DOWN
60	C605987.1	gb|EDV43759.1||GF16361 [Drosophila ananassae]; gb|EDV92742.1||GH18927 [Drosophila grimshawi]; gb|AAD39600.1|AC007858_14|10A19I.3 [Oryza sativa (japonica cultivar-group)]; ref|NP_001056416.1||Os05g0579000 [Oryza sativa (japonica cultivar-group)] gb|AAU10692.1| putative sugar transporter [Oryza sativa (japonica cultivar-group)] dbj|BAF18330.1| Os05g0579000 [Oryza sativa (japonica cultivar-group)];	-1,024202408	-0,856317488	-1,136497056	-1,104028481	-1,064115445	2,1	DOWN
61	C609884.1	gb|AAX24837.2||SJCHGC02515 protein [Schistosoma japonicum];	-1,196869826	-1,161990072	-0,964217854	-0,902551366	-1,063103963	2,1	DOWN
62	C604379.1	No match	-1,152521304	-1,084490637	-1,01756126	-1,041607049	-1,063048843	2,1	DOWN
63	C601898.1	gb|EDW77741.1||GK24389 [Drosophila willistoni]; gb|EDV30846.1||GF15062 [Drosophila ananassae]; gb|EDW90296.1||GE13195 [Drosophila yakuba]; gb|EDV54773.1||GG21122 [Drosophila erecta];	-0,703664993	-0,791624053	-1,421332845	-1,329367768	-1,060495911	2,1	DOWN
64	C606373.1	gb|AAP06363.1||hypothetical protein [Schistosoma japonicum]; gb|ACE06787.1||unknown [Schistosoma japonicum];	-1,278084033	-1,249687011	-0,863691577	-0,822719011	-1,056689294	2,1	DOWN
65	C602181.1	sp|P49965|SRP72_SCHMA|Signal recognition particle 72 kDa protein (SRP72) gb|AAA69689.1| signal recognition particle 72 kDa subunit; gb|AAW27494.1||unknown [Schistosoma japonicum];	-1,014181874	-1,008980481	-1,094446841	-1,108778576	-1,054314358	2,1	DOWN
66	C611543.1	gb|AAW25713.1||SJCHGC00391 protein [Schistosoma japonicum]; gb|AAW26336.1||unknown [Schistosoma japonicum]; ref|NP_998068.1||eukaryotic translation initiation factor 2B, subunit 2 beta [Danio rerio] gb|AAH67147.1| Eukaryotic translation initiation factor 2B, subunit 2 beta [Danio rerio] emb|CAH69026.1| novel protein (zgc:77512) [Danio rerio]; dbj|BAC36248.1||unnamed protein product [Mus musculus];	-1,081834815	-1,041044508	-0,990217201	-1,056064965	-1,048554737	2,1	DOWN
67	C600688.1	gb|AAX25206.2||SJCHGC02554 protein [Schistosoma japonicum];	-0,72622152	-1,051284523	-1,041515998	-1,041759708	-1,041637853	2,1	DOWN

68	C607201.1	gb|AAX28601.2||SJCHGC08387 protein [Schistosoma japonicum]; gb|AAG01032.1||Stat3-interacting protein [Mus musculus]; dbj|BAE39871.1||unnamed protein product [Mus musculus]; dbj|BAE39766.1||unnamed protein product [Mus musculus];	-1,02707029	-0,901615664	-1,055437831	-1,181268215	-1,041254061	2,1	DOWN
69	C607961.1	gb|ACE06868.1||unknown [Schistosoma japonicum]; gb|AAC69992.1||tetraspanin TE736 [Schistosoma japonicum]; gb|AAW24656.1||unknown [Schistosoma japonicum]; gb|AAW27678.1||unknown [Schistosoma japonicum];	-0,849241796	-0,937858865	-1,151337883	-1,139441123	-1,038649994	2,1	DOWN
70	C612131.1	No match	-1,131611412	-1,171233445	-0,94033123	-0,852997818	-1,035971321	2,1	DOWN
71	C606800.1	No match	-0,72473017	-0,952395236	-1,2114256	-1,114903974	-1,033649605	2,0	DOWN
72	C608727.1	gb|AAX27988.2||SJCHGC05935 protein [Schistosoma japonicum]; gb|AAW27057.1||SJCHGC05282 protein [Schistosoma japonicum]; emb|CAF99798.1||unnamed protein product [Tetraodon nigroviridis]; ref|XP_416562.2||PREDICTED: similar to MGC69357 protein [Gallus gallus];	-1,272229836	-1,1518321	-0,915246485	-0,889046859	-1,033539293	2,0	DOWN
73	C608278.1	No match	-1,10177231	-1,057965446	-0,973328313	-1,008603844	-1,033284645	2,0	DOWN
74	C606420.1	gb|AAX30499.2||SJCHGC04165 protein [Schistosoma japonicum]; ref|XP_001120146.1||PREDICTED: similar to CG8320-PA [Apis mellifera]; ref|NP_001040235.1||hypothetical protein LOC692928 [Bombyx mori] gb|ABD36286.1| unknown [Bombyx mori]; gb|ABI52781.1||DUF788 membrane protein [Argas monolakensis];	-1,107635192	-1,099179469	-0,966176558	-0,964157567	-1,032678014	2,0	DOWN
75	C606543.1	tpe|CAJ00236.1||TPA: endonuclease-reverse transcriptase [Schistosoma mansoni]; gb|AAY56755.1||reverse transcriptase SR3-left [Schistosoma mansoni]; gb|AAY56756.1||reverse transcriptase SR3-right [Schistosoma mansoni];	-1,067551847	-1,186203072	-0,926614506	-0,997295526	-1,032423687	2,0	DOWN
76	C605045.1	gb|AAP05921.1||similar to NM_024231 zinc finger like protein 1 [Schistosoma japonicum]; gb|ACE06939.1||unknown [Schistosoma japonicum];	-1,224384889	-1,033846607	-1,030250226	-0,877254526	-1,032048417	2,0	DOWN
77	C608792.1	ref|NP_001026008.1||ribonucleoside-diphosphate reductase M1 chain [Gallus gallus] emb|CAG31174.1| hypothetical protein [Gallus gallus]; ref|XP_001746543.1||predicted protein [Monosiga brevicollis MX1] gb|EDQ88439.1| predicted protein [Monosiga brevicollis MX1]; ref|XP_001505371.1||PREDICTED: similar to M1 subunit of ribonucleotide reductase [Ornithorhynchus anatinus]; ref|XP_968671.1||PREDICTED: similar to AGAP010198-PA [Tribolium castaneum];	-1,154538565	-1,199279308	-0,905183622	-0,759418163	-1,029861094	2,0	DOWN
78	C602575.1	gb|ABL86191.1||unknown [Schistosoma japonicum]; gb|AAW27089.1||unknown [Schistosoma japonicum]; gb|AAW26412.1||SJCHGC00398 protein [Schistosoma japonicum]; gb|AAW27337.1||unknown [Schistosoma japonicum];	-1,14107812	-1,030892638	-1,020795895	-1,015031104	-1,025844267	2,0	DOWN
79	C605938.1	gb|AAW27043.1||SJCHGC08900 protein [Schistosoma japonicum];	-0,707866714	-0,724822329	-1,3751306	-1,321814106	-1,023318218	2,0	DOWN
80	C608377.1	No match	-0,778100556	-0,936269349	-1,145380355	-1,109646592	-1,022957971	2,0	DOWN
81	C608749.1	gb|AAW26930.1||SJCHGC02342 protein [Schistosoma japonicum];	-0,856406365	-0,94412924	-1,206142181	-1,101518477	-1,022823859	2,0	DOWN

82	C612252.1	gb|AAW24984.1||SJCHGC06626 protein [Schistosoma japonicum]; emb|CAG06397.1||unnamed protein product [Tetraodon nigroviridis]; ref|NP_001007769.1||phenylalanine-tRNA synthetase-like [Danio rerio] gb|AAH85625.1| Phenylalanine-tRNA synthetase-like [Danio rerio]; ref|XP_864045.1||PREDICTED: similar to Phenylalanyl-tRNA synthetase beta chain (Phenylalanine-tRNA ligase beta chain) (PheRS) isoform 3 [Canis familiaris];	-0,675204497	-0,891144162	-1,137404483	-1,185982905	-1,014274323	2,0	DOWN
83	C607022.1	No match	-0,971549725	-1,033877217	-1,122351716	-0,990222423	-1,01204982	2,0	DOWN
84	C606084.1	tpg|DAA04500.1||TPA_exp: pol polyprotein [Schistosoma mansoni]; tpg|DAA04502.1||TPA_exp: ORF3 [Schistosoma mansoni];	-1,005217173	-0,853512339	-1,13796554	-1,015142466	-1,01017982	2,0	DOWN
85	C601007.1	ref|XP_001896274.1||UDP-sugar transporter-like protein [Brugia malayi] gb|EDP34887.1| UDP-sugar transporter-like protein [Brugia malayi]; ref|XP_395030.3||PREDICTED: similar to fringe connection CG3874-PA [Apis mellifera]; ref|XP_001637195.1||predicted protein [Nematostella vectensis] gb|EDO45132.1| predicted protein [Nematostella vectensis];	-0,721963963	-0,651245717	-1,403196664	-1,298080402	-1,010022183	2,0	DOWN
86	C610237.1	tpe|CAJ00235.1||TPA: endonuclease-reverse transcriptase [Schistosoma mansoni]; tpe|CAJ00242.1||TPA: endonuclease-reverse transcriptase [Schistosoma mansoni]; tpe|CAJ00240.1||TPA: endonuclease-reverse transcriptase [Schistosoma mansoni];	-0,834999256	-0,914959042	-1,137436533	-1,105064557	-1,0100118	2,0	DOWN
87	C610832.1	gb|AAX26272.2||SJCHGC07577 protein [Schistosoma japonicum]; ref|XP_320556.4||AGAP011975-PA [Anopheles gambiae str. PEST] gb|EAA00315.4| AGAP011975-PA [Anopheles gambiae str. PEST]; ref|XP_001651941.1||hypothetical protein AaeL_AAEL006337 [Aedes aegypti] gb|EAT42101.1| conserved hypothetical protein [Aedes aegypti]; ref|XP_001602901.1||PREDICTED: similar to conserved hypothetical protein [Nasonia vitripennis];	-1,030450519	-1,128743129	-0,913115359	-0,987735809	-1,009093164	2,0	DOWN
88	C609365.1	gb|AAW27442.1||SJCHGC02482 protein [Schistosoma japonicum]; gb|AAW27607.1||SJCHGC02483 protein [Schistosoma japonicum]; ref|XP_001490497.2||PREDICTED: similar to MIT, microtubule interacting and transport, domain containing 1 [Equus caballus]; ref|XP_001368545.1||PREDICTED: hypothetical protein [Monodelphis domestica];	-0,861492541	-0,869590884	-1,192971497	-1,142907842	-1,006249363	2,0	DOWN
89	C602638.1	ref|XP_974460.1||PREDICTED: similar to CG9752 CG9752-PA [Tribolium castaneum]; ref|XP_394550.2||PREDICTED: similar to CG9752-PA [Apis mellifera]; gb|EDW74027.1||GK21592 [Drosophila willistoni]; ref|XP_001604967.1||PREDICTED: similar to conserved hypothetical protein [Nasonia vitripennis];	-0,969128821	-1,071383009	-1,043014461	-0,91395858	-1,006071641	2,0	DOWN
90	C602560.1	ref|XP_972549.2||PREDICTED: similar to RNA (guanine-9-)-methyltransferase domain-containing protein 1 homolog, mitochondrial [Tribolium castaneum]; gb|EDW08762.1||GI20132 [Drosophila mojavensis]; gb|EDW02745.1||GH19713 [Drosophila grimshawi]; gb|EDW85720.1||GK23002 [Drosophila willistoni];	-0,958552418	-1,041765786	-1,344150694	-0,948003993	-1,000159102	2,0	DOWN

91	C604190.1	ref|XP_535891.2||PREDICTED: similar to NAD-dependent deacetylase sirtuin-5 (SIR2-like protein 5) [Canis familiaris]; ref|XP_001915269.1||PREDICTED: similar to sirtuin [Equus caballus]; gb|ABY65335.1||sirtuin 5 [Sus scrofa]; ref|NP_849179.1||sirtuin 5 (silent mating type information regulation 2 homolog) 5 [Mus musculus] sp|Q8K2C6|SIRT5_MOUSE NAD-dependent deacetylase sirtuin-5 (SIR2-like protein 5) gb|AAH31770.1| Sirtuin 5 (silent mating type information regulation 2 homolog) 5 (S. cerevisiae) [Mus musculus] gb|EDL40991.1| sirtuin 5 (silent mating type information regulation 2 homolog) 5 (S. cerevisiae) [Mus musculus];	-0,968060322	-1,031921667	-1,342327491	-0,95264541	-0,999990995	2,0	DOWN
92	C610421.1	gb|AAX25786.2||SJCHGC06308 protein [Schistosoma japonicum]; ref|XP_001950180.1||PREDICTED: similar to FCH domain only 2 [Acyrthosiphon pisum]; ref|XP_972256.2||PREDICTED: similar to FCH domain only 2 [Tribolium castaneum]; gb|EDW15768.1||GI10156 [Drosophila mojavensis];	-0,922180884	-0,898209352	-1,073895162	-1,087906993	-0,998038023	2,0	DOWN
93	C603786.1	gb|AAW26600.1||SJCHGC06283 protein [Schistosoma japonicum]; ref|XP_001640523.1||predicted protein [Nematostella vectensis] gb|EDO48460.1| predicted protein [Nematostella vectensis]; gb|EDW43046.1||GM23722 [Drosophila sechellia]; gb|EDV43144.1||GF18341 [Drosophila ananassae] gb|EDV49939.1| GG24917 [Drosophila erecta] gb|EDV91406.1| GH14195 [Drosophila grimshawi] gb|EDW15501.1| GI24868 [Drosophila mojavensis] gb|EDW68059.1| GJ24512 [Drosophila virilis] gb|EDW96503.1| GE25867 [Drosophila yakuba];	-1,030509833	-1,20051242	-0,931672781	-0,963255727	-0,99688278	2,0	DOWN
94	C602475.1	gb|AAW27690.1||SJCHGC04924 protein [Schistosoma japonicum]; emb|CAD21532.1||hypothetical protein [Taenia solium];	-0,990726052	-1,131634861	-1,000342966	-0,980379837	-0,995534509	2,0	DOWN
95	C609066.1	gb|AAX27215.2||SJCHGC03451 protein [Schistosoma japonicum]; ref|XP_001518734.1||PREDICTED: hypothetical protein, partial [Ornithorhynchus anatinus]; sp|Q8VDQ9.2|KRI1_MOUSE|Protein KRI1 homolog; ref|NP_663391.2||KRI1 homolog [Mus musculus] gb|EDL25175.1| cDNA sequence BC021438 [Mus musculus];	-0,971781897	-0,248839233	-1,063145379	-1,018806191	-0,995294044	2,0	DOWN
96	C602228.1	No match	-1,07668	-1,046097829	-0,944316846	-0,94142386	-0,995207338	2,0	DOWN
97	C612384.1	gb|AAW27684.1||SJCHGC08954 protein [Schistosoma japonicum]; ref|NP_001033227.1||PRELI domain containing 1 [Bos taurus] sp|Q32KN9|PRLD1_BOVIN PRELI domain-containing protein 1, mitochondrial precursor gb|AAI10002.1| PRELI domain containing 1 [Bos taurus]; dbj|BAE29582.1||unnamed protein product [Mus musculus]; ref|XP_001169493.1||PREDICTED: similar to CGI-106 protein isoform 3 [Pan troglodytes];	-1,049279841	-1,28004941	-0,936855288	-0,836302429	-0,993067565	2,0	DOWN
98	C609895.1	gb|AAZ57334.1||CREB-binding protein [Schistosoma mansoni]; gb|AAX24597.2||SJCHGC08730 protein [Schistosoma japonicum];	-0,710461744	-0,87854555	-1,193726101	-1,10289025	-0,9907179	2,0	DOWN

99	C603418.1	gb|AAW27439.2||SJCHGC02252 protein [Schistosoma japonicum]; gb|AAX27473.2||SJCHGC02253 protein [Schistosoma japonicum]; ref|XP_308251.4||AGAP007620-PA [Anopheles gambiae str. PEST] gb|EAA04505.4| AGAP007620-PA [Anopheles gambiae str. PEST]; sp|Q03751|CSP_DROME|Cysteine string protein gb|AAA28432.1| csp32;	-0,968860177	-1,15092698	-0,949378104	-1,012201635	-0,990530906	2,0	DOWN
100	C601295.1	sp|P19331|IM23_SCHMA|23 kDa integral membrane protein (Sm23) gb|AAA29900.1| integral membrane protein; gb|AAA73525.1||integral membrane protein 23; sp|Q26499|IM23_SCHHA|23 kDa integral membrane protein (Sh23) gb|AAC46959.1| Sh23 [Schistosoma haematobium]; gb|ABS19440.1||multivalent antigen sjTPI-23 [synthetic construct];	-0,787545563	-1,063120634	-0,967351467	-1,012688869	-0,990020168	2,0	DOWN
101	C711036.1	ref|XP_001380141.1||PREDICTED: hypothetical protein [Monodelphis domestica]; ref|XP_001625397.1||predicted protein [Nematostella vectensis] gb|EDO33297.1| predicted protein [Nematostella vectensis]; gb|AAM18868.1|AF391288_4|unknown [Branchiostoma floridae]; ref|XP_001599787.1||PREDICTED: similar to wd-repeat protein [Nasonia vitripennis];	-1,186096365	-1,082508927	-0,823428615	-0,895804972	-0,98915695	2,0	DOWN
102	C604966.1	gb|AAI28795.1||Aars protein [Danio rerio]; ref|NP_001037775.1||alanyl-tRNA synthetase [Danio rerio] emb|CAK04586.1| alanyl-tRNA synthetase [Danio rerio]; emb|CAQ13590.1||novel protein similar to vertebrate alanyl-tRNA synthetase (AARS) [Danio rerio]; emb|CAK04587.1||alanyl-tRNA synthetase [Danio rerio];	-0,943924193	-0,980214538	-1,045364207	-0,995415199	-0,987814869	2,0	DOWN
103	C605577.1	ref|XP_973990.1||PREDICTED: similar to O-fucosyltransferase, putative [Tribolium castaneum]; ref|NP_001037061.2||protein-O-fucosyltransferase 1 [Bombyx mori] gb|ABA29467.1| protein O-fucosyltransferase 1 [Bombyx mori]; emb|CAH40835.1||protein-O-fucosyltransferase 1 [Bombyx mori]; gb|ABA29465.1||protein O-fucosyltransferase 1 [Aedes aegypti];	-0,9372823	-0,781778951	-1,088789111	-1,026198006	-0,981740153	2,0	DOWN
104	C610121.1	gb|AAC79802.3||annexin [Schistosoma mansoni]; gb|ACC78610.1||annexin [Schistosoma bovis]; gb|AAW25344.1||SJCHGC06760 protein [Schistosoma japonicum]; gb|AAD34598.1|AF147955_1|antigen cC1 [Taenia solium];	-0,740030497	-0,573915943	-1,355218849	-1,216652165	-0,978341331	2,0	DOWN
105	C612596.1	No match	-0,966164515	-0,988132163	-0,808007658	-1,048173202	-0,977148339	2,0	DOWN
106	C610195.1	No match	-0,756372033	-0,841899356	-1,223602458	-1,112078898	-0,976989127	2,0	DOWN
107	C603901.1	gb|AAW26656.1||SJCHGC09074 protein [Schistosoma japonicum];	-0,964147385	-0,978198318	-1,032387582	-0,915050753	-0,971172852	2,0	DOWN
108	C607341.1	No match	-0,818688682	-0,978015355	-1,035372581	-0,96119463	-0,969604993	2,0	DOWN
109	C605188.1	gb|AAW27105.1||SJCHGC03317 protein [Schistosoma japonicum]; gb|EDW06752.1||GI15232 [Drosophila mojavensis]; gb|EDW65808.1||GJ18691 [Drosophila virilis]; gb|EDW86514.1||GK14620 [Drosophila willistoni];	-0,653151803	-0,74977649	-1,18494236	-1,220570914	-0,967359425	2,0	DOWN
110	C608724.1	No match	-0,881475989	-1,00306577	-1,061835204	-0,930719949	-0,96689286	2,0	DOWN
111	C612307.1	tpe|CAJ00236.1||TPA: endonuclease-reverse transcriptase [Schistosoma mansoni]; gb|AAX26802.2||SJCHGC02939 protein [Schistosoma japonicum];	-0,700550993	-0,752097738	-1,267305406	-1,180321581	-0,96620966	2,0	DOWN

112	C603749.1	gb|AAP06016.2||SJCHGC01960 protein [Schistosoma japonicum]; gb|AAW26587.1||SJCHGC09469 protein [Schistosoma japonicum]; emb|CAG02594.1||unnamed protein product [Tetraodon nigroviridis]; ref|NP_001006882.1||heat shock 10kDa protein 1 [Xenopus tropicalis] gb|AAH77653.1| Heat shock 10kDa protein 1 (chaperonin 10) [Xenopus tropicalis] emb|CAJ81476.1| heat shock 10kDa protein 1 (chaperonin 10) [Xenopus tropicalis];	-1,251656914	-1,244720404	-0,687070375	-0,646771551	-0,96589539	2,0	DOWN
113	C611581.1	gb|AAW26671.1||SJCHGC02427 protein [Schistosoma japonicum]; gb|AAX28339.1||unknown [Schistosoma japonicum]; sp|Q8AXS6|S35B1_XENTR|Solute carrier family 35 member B1 emb|CAJ82891.1| solute carrier family 35, member B1 [Xenopus tropicalis]; ref|NP_989418.1||endoplasmic reticulum nucleotide sugar transporter [Xenopus tropicalis] emb|CAD54627.1| endoplasmic reticulum nucleotide sugar transporter [Xenopus tropicalis];	-1,00977753	-1,01422161	-0,901342094	-0,917697548	-0,963737539	2,0	DOWN
114	C605890.1	ref|XP_001494366.1||PREDICTED: ATPase, aminophospholipid transporter (APLT), class I, type 8A, member 1 [Equus caballus]; ref|NP_777263.1||ATPase, aminophospholipid transporter (APLT), Class I, type 8A, member 1 [Bos taurus] sp|Q29449|AT8A1_BOVIN Probable phospholipid-transporting ATPase IA (Chromaffin granule ATPase II) (ATPase class I type 8A member 1) gb|AAD03352.1| chromaffin granule ATPase II [Bos taurus]; ref|XP_420729.2||PREDICTED: similar to ATPase II [Gallus gallus]; ref|XP_001337058.2||PREDICTED: similar to ATPase, aminophospholipid transporter (APLT), class I, type 8A, member 1, partial [Danio rerio];	-0,798676287	-0,924774514	-0,99805464	-1,028031378	-0,961414577	1,9	DOWN
115	C610316.1	gb|AAX24987.2||SJCHGC04429 protein [Schistosoma japonicum];	-0,811426295	-0,890323644	-1,030898612	-1,110034045	-0,960611128	1,9	DOWN
116	C602195.1	gb|AAX26785.2||SJCHGC05057 protein [Schistosoma japonicum]; ref|XP_001478203.1||PREDICTED: similar to Inosine 5-phosphate dehydrogenase 2 [Mus musculus]; gb|AAA39311.1||IMP dehydrogenase (EC 1.2.1.14); ref|NP_035960.2||inosine 5'-phosphate dehydrogenase 2 [Mus musculus] sp|P24547|IMDH2_MOUSE Inosine-5'-monophosphate dehydrogenase 2 (IMP dehydrogenase 2) (IMPDH-II) (IMPD 2) gb|AAH10314.1| Inosine 5'-phosphate dehydrogenase 2 [Mus musculus] gb|AAH52671.1| Inosine 5'-phosphate dehydrogenase 2 [Mus musculus] dbj|BAE35879.1| unnamed protein product [Mus musculus] dbj|BAE40908.1| unnamed protein product [Mus musculus] dbj|BAE41367.1| unnamed protein product [Mus musculus] dbj|BAE38929.1| unnamed protein product [Mus musculus] dbj|BAE29791.1| unnamed protein product [Mus musculus] dbj|BAE30225.1| unnamed protein product [Mus musculus] dbj|BAE35402.1| unnamed protein product [Mus musculus] dbj|BAE31422.1| unnamed protein product [Mus musculus] gb|EDL21302.1| inosine 5'-phosphate dehydrogenase 2 [Mus musculus];	-1,01472003	-0,931928806	-0,980234353	-0,940565324	-0,960399839	1,9	DOWN

117	C603420.1	gb|AAX26129.2||SJCHGC06300 protein [Schistosoma japonicum]; ref|XP_001602516.1||PREDICTED: similar to MGC53562 protein [Nasonia vitripennis]; ref|NP_001079679.1||hypothetical protein LOC379366 [Xenopus laevis] sp|Q7ZWN0.1|LMF2_XENLA Lipase maturation factor 2 gb|AAH46869.1| Lmf2 protein [Xenopus laevis]; ref|NP_001072170.1||hypothetical protein LOC594899 [Xenopus tropicalis] sp|Q0P4Y8.1|LMF2_XENTR Lipase maturation factor 2 gb|AAI21840.1| Lipase maturation factor 2 [Xenopus tropicalis];	#DIV/0!	-0,04390379	-0,959593214	-1,024477707	-0,959593214	1,9	DOWN
118	C602495.1	gb|AAW25367.1||unknown [Schistosoma japonicum]; gb|AAW24629.1||SJCHGC01322 protein [Schistosoma japonicum]; gb|AAK15271.1|AF308144_1|Sj-Ts4 protein [Schistosoma japonicum]; gb|AAX25898.2||SJCHGC07013 protein [Schistosoma japonicum];	-0,648175843	-0,828497035	-1,251488703	-1,090413804	-0,95945542	1,9	DOWN
119	C602650.1	No match	-1,073461763	-1,078416108	-0,838445712	-0,775430871	-0,955953738	1,9	DOWN
120	C607625.1	No match	-0,494637862	-0,72796806	-1,18354495	-1,411303326	-0,955756505	1,9	DOWN
121	C608302.1	gb|AAX25184.2||SJCHGC08832 protein [Schistosoma japonicum];	-0,664897261	-0,749908118	-1,159865564	-1,177403561	-0,954886841	1,9	DOWN
122	C610520.1	gb|AAX27136.2||SJCHGC07961 protein [Schistosoma japonicum]; ref|XP_001942990.1||PREDICTED: similar to SEC63 protein, putative [Acyrthosiphon pisum]; ref|XP_001638715.1||predicted protein [Nematostella vectensis] gb|EDO46652.1| predicted protein [Nematostella vectensis]; gb|EDX09557.1||GD14009 [Drosophila simulans];	-0,962060597	-0,978601378	-0,915869311	-0,940996852	-0,951528725	1,9	DOWN
123	C609184.1	No match	-0,965157527	-0,729974837	-0,936388243	-0,966737549	-0,950772885	1,9	DOWN
124	C609423.1	gb|AAW27625.1||SJCHGC01869 protein [Schistosoma japonicum];	-1,098154078	-0,864516793	-1,026912182	-0,848812828	-0,945714488	1,9	DOWN
125	C610194.1	No match	-0,836088557	-0,774540783	-1,097643125	-1,054711148	-0,945399853	1,9	DOWN
126	C611344.1	gb|AAP06286.1||SJCHGC00554 protein [Schistosoma japonicum]; gb|AAX27560.2||SJCHGC02038 protein [Schistosoma japonicum]; ref|XP_793449.2||PREDICTED: hypothetical protein [Strongylocentrotus purpuratus] ref|XP_001178548.1| PREDICTED: hypothetical protein [Strongylocentrotus purpuratus]; ref|NP_001090309.1||hypothetical protein LOC779218 [Xenopus laevis] gb|AAI23144.1| MGC154331 protein [Xenopus laevis];	-0,861361953	-0,77086676	-1,022244254	-1,025744743	-0,941803104	1,9	DOWN
127	C601552.1	No match	-0,772803102	-0,6454877	-1,108961294	-1,106975939	-0,939889521	1,9	DOWN
128	C603352.1	gb|AAW27868.1||SJCHGC06337 protein [Schistosoma japonicum]; gb|AAW27771.1||SJCHGC01525 protein [Schistosoma japonicum]; gb|AAW25162.1||unknown [Schistosoma japonicum]; dbj|BAD08526.1||17-beta hydroxysteroid dehydrogenase [Ciona intestinalis];	-1,215205718	-1,053562543	-0,817642804	-0,722569299	-0,935602674	1,9	DOWN
129	C612230.1	gb|AAX27733.2||SJCHGC06480 protein [Schistosoma japonicum]; ref|XP_395702.2||PREDICTED: similar to Xeroderma pigmentosum group A-like CG6358-PA [Apis mellifera]; dbj|BAA06690.1||Dxpa protein [Drosophila melanogaster]; ref|NP_476866.1||Xeroderma pigmentosum group A-like CG6358-PA [Drosophila melanogaster] sp|P28518|XPA_DROME DNA-repair protein complementing XP-A cells homolog (Xeroderma pigmentosum group A-complementing protein homolog) emb|CAA21834.1| EG:EG0007.8 [Drosophila melanogaster] gb|AAF45917.1| CG6358-PA [Drosophila melanogaster];	-0,946429437	-1,031890244	-0,923002075	-0,911969384	-0,934715756	1,9	DOWN

130	C606835.1	No match	-0,713709284	-0,77484006	-1,207541709	-1,088284164	-0,931562112	1,9	DOWN
131	C607154.1	gb|AAW27297.1||SJCHGC01783 protein [Schistosoma japonicum]; gb|EDV25878.1||expressed hypothetical protein [Trichoplax adhaerens]; emb|CAB46230.1||rab GDP-dissociation inhibitor [Branchiostoma floridae]; ref|XP_623197.2||PREDICTED: similar to GDP dissociation inhibitor CG4422-PA [Apis mellifera];	-0,734614123	-0,900583657	-1,00265488	-0,961765872	-0,931174765	1,9	DOWN
132	C607081.1	gb|AAX30490.2||SJCHGC04027 protein [Schistosoma japonicum]; gb|EDV25795.1||hypothetical protein TRIADDRAFT_23475 [Trichoplax adhaerens];	-0,826901307	-0,795324085	-1,032337024	-1,119710872	-0,929619166	1,9	DOWN
133	C714068.1	gb|AAX30649.1||SJCHGC06493 protein [Schistosoma japonicum]; ref|XP_001120892.1||PREDICTED: similar to CG14214-PA [Apis mellifera]; gb|EDV20899.1||conserved hypothetical protein [Trichoplax adhaerens]; ref|NP_001037674.1||transport protein Sec61 gamma subunit [Bombyx mori] gb|ABF51395.1| transport protein SEC61 [Bombyx mori] gb|ABF85697.1| transport protein Sec61 gamma subunit [Bombyx mori];	-0,496983746	-0,483967769	-1,36375159	-1,355571711	-0,926277729	1,9	DOWN
134	C600001.1	gb|AAX26813.2||SJCHGC05893 protein [Schistosoma japonicum]; ref|XP_780071.2||PREDICTED: similar to Chimerin (chimaerin) 1 [Strongylocentrotus purpuratus] ref|XP_001195747.1| PREDICTED: similar to Chimerin (chimaerin) 1 [Strongylocentrotus purpuratus]; ref|NP_497323.3||BE0003N10.2 [Caenorhabditis elegans]; gb|AAK73856.2|AC092690_2|Hypothetical protein BE0003N10.2 [Caenorhabditis elegans];	-0,872167559	-1,063087353	-0,950485725	-0,893912197	-0,922198961	1,9	DOWN
135	C611836.1	No match	-0,912079809	-1,001683527	-0,789738982	-0,929092382	-0,920586096	1,9	DOWN
136	C609023.1	gb|AAX27464.2||SJCHGC08006 protein [Schistosoma japonicum]; gb|AAP06311.1||hypothetical protein [Schistosoma japonicum];	-0,693691374	-0,707218199	-1,251670436	-1,130341686	-0,918779943	1,9	DOWN
137	C611483.1	No match	-0,850153466	-0,742348696	-1,063103345	-0,985924511	-0,918038989	1,9	DOWN
138	C609782.1	gb|AAG52888.1|AF333765_1|DAD-1-like protein [Schistosoma japonicum] gb|ACE06891.1| unknown [Schistosoma japonicum]; ref|XP_001123277.1||PREDICTED: similar to CG13393-PA [Apis mellifera]; gb|ABM55607.1||dolichyl-diphosphooligosaccharide--protein glycosyltransferase subunit DAD1-like protein [Maconellicoccus hirsutus]; ref|XP_966764.1||PREDICTED: similar to CG13393 CG13393-PA [Tribolium castaneum];	-0,936751902	-0,898705249	-0,982194022	-0,778800347	-0,917728576	1,9	DOWN
139	C601649.1	gb|AAX30160.2||SJCHGC01212 protein [Schistosoma japonicum]; ref|YP_873485.1||beta-lactamase domain protein [Acidothermus cellulolyticus 11B] gb|ABK53499.1| beta-lactamase domain protein [Acidothermus cellulolyticus 11B]; ref|XP_001949112.1||PREDICTED: similar to CG9117 CG9117-PA [Acyrthosiphon pisum]; ref|XP_692640.2||PREDICTED: similar to CG9117 CG9117-PA [Danio rerio];	-0,880709302	-0,936223645	-0,936314327	-0,899158874	-0,91769126	1,9	DOWN
140	C612154.1	gb|AAW27619.1||SJCHGC06261 protein [Schistosoma japonicum]; ref|XP_001915357.1||PREDICTED: similar to BRO1 domain-containing protein BROX [Equus caballus]; ref|XP_600599.3||PREDICTED: hypothetical protein [Bos taurus]; dbj|BAB71331.1||unnamed protein product [Homo sapiens] dbj|BAF56045.1| Bro1-domain-containing protein [Homo sapiens];	-0,882510863	-1,291703903	-0,869186866	-0,950765079	-0,916637971	1,9	DOWN

141	C603400.1	gb|AAW25867.1||SJCHGC02436 protein [Schistosoma japonicum]; ref|XP_001604174.1||PREDICTED: similar to HDC19127 [Nasonia vitripennis]; ref|NP_001018527.1||hypothetical protein LOC553720 [Danio rerio] gb|AAH95784.1| Zgc:112365 [Danio rerio] gb|AAI64762.1| Zgc:112365 [synthetic construct]; ref|NP_001096035.1||alkB, alkylation repair homolog 1 [Mus musculus];	-1,166996569	-0,90720598	-0,912362186	-0,910402101	-0,911382144	1,9	DOWN
142	C603357.1	No match	-1,061824895	-1,003819201	-0,735631435	-0,812668227	-0,908243714	1,9	DOWN
143	C608968.1	No match	-0,635287775	-0,651283872	-1,205872403	-1,157578249	-0,904431061	1,9	DOWN
144	C611828.1	gb|AAX24999.2||SJCHGC04012 protein [Schistosoma japonicum];	-1,083314683	-1,073619832	-0,726643063	-0,667324661	-0,900131448	1,9	DOWN
145	C611706.1	gb|AAW25843.1||unknown [Schistosoma japonicum]; gb|AAW24972.1||SJCHGC01067 protein [Schistosoma japonicum]; gb|EDV57554.1||GG24473 [Drosophila erecta]; ref|XP_001201855.1||PREDICTED: hypothetical protein, partial [Strongylocentrotus purpuratus] ref|XP_796678.2| PREDICTED: hypothetical protein, partial [Strongylocentrotus purpuratus];	-0,780310572	-0,868088416	-1,037301328	-0,926779355	-0,897433886	1,9	DOWN
146	C605248.1	gb|AAW24880.1||SJCHGC06172 protein [Schistosoma japonicum]; gb|ABY56687.1||Ts3 protein [Taenia solium]; ref|XP_001362363.1||PREDICTED: similar to ribosomal protein L18a [Monodelphis domestica]; gb|AAH42256.1||RPL18A protein [Xenopus laevis];	-0,648077044	-0,668725565	-1,122897732	-1,198689532	-0,895811649	1,9	DOWN
147	C601166.1	No match	-1,074784113	-1,217694422	-0,710196788	-0,710483909	-0,892634011	1,9	DOWN
148	C602909.1	No match	-0,523106523	-0,554337974	-1,230233748	-1,246708312	-0,892285861	1,9	DOWN
149	C610721.1	gb|AAW27711.1||SJCHGC02387 protein [Schistosoma japonicum]; gb|AAW25706.1||SJCHGC02386 protein [Schistosoma japonicum];	-0,877516761	-0,744188818	-0,903497332	-1,006015995	-0,890507047	1,9	DOWN
150	C607539.1	gb|AAX26809.2||SJCHGC06221 protein [Schistosoma japonicum];	-0,784386476	-0,788845002	-0,983086455	-1,004182934	-0,885965729	1,8	DOWN
151	C604842.1	No match	-0,73190702	-0,799371717	-1,031486469	-0,970823778	-0,885097748	1,8	DOWN
152	C602617.1	No match	-0,885093633	-0,819398995	-0,905731569	-0,880661047	-0,88287734	1,8	DOWN
153	C713563.1	gb|AAW27569.1||SJCHGC03345 protein [Schistosoma japonicum]; gb|ABO77964.1||nanos-like protein [Schmidtea mediterranea]; gb|ABO52809.1||nanos-like protein [Schmidtea mediterranea]; dbj|BAD88623.1||nanos-related protein [Dugesia japonica];	-0,826040095	-0,884872613	-0,878705308	-0,948456483	-0,881788961	1,8	DOWN
154	C605824.1	gb|AAW25423.1||SJCHGC06005 protein [Schistosoma japonicum];	-0,782308052	-0,882669684	-0,879707325	-1,277692888	-0,881188505	1,8	DOWN
155	C603972.1	gb|AAA29882.1||fimbrin; gb|AAX24922.2||SJCHGC01985 protein [Schistosoma japonicum]; gb|EDW05622.1||GI11134 [Drosophila mojavensis]; gb|EDV46485.1||GG18187 [Drosophila erecta];	-0,879935851	-0,879877699	-0,933398464	-0,852243172	-0,879906775	1,8	DOWN
156	C603408.1	No match	-1,099889263	-1,120736647	-0,656798853	-0,586580765	-0,878344058	1,8	DOWN
157	C603897.1	gb|AAV49163.1||polo-like kinase [Schistosoma mansoni]; ref|XP_001915939.1||PREDICTED: similar to Serine/threonine-protein kinase PLK1 (Polo-like kinase 1) (PLK-1) (Serine/threonine-protein kinase 13) (STPK13) [Equus caballus]; ref|XP_547091.2||PREDICTED: similar to Serine/threonine-protein kinase PLK1 (Polo-like kinase 1) (PLK-1) (Serine/threonine-protein kinase 13) (STPK13) [Canis familiaris]; dbj|BAG36038.1||unnamed protein product [Homo sapiens];	-0,552743924	-0,824852894	-0,915968268	-1,032814777	-0,870410581	1,8	DOWN

158	C606776.1	gb|AAW24796.1||SJCHGC06638 protein [Schistosoma japonicum]; gb|AAW25340.1||SJCHGC09559 protein [Schistosoma japonicum]; ref|XP_788872.1||PREDICTED: similar to Adenine phosphoribosyl transferase [Strongylocentrotus purpuratus] ref|XP_001202862.1| PREDICTED: similar to Adenine phosphoribosyl transferase [Strongylocentrotus purpuratus]; ref|ZP_01741785.1||adenine phosphoribosyltransferase [Rhodobacterales bacterium HTCC2150] gb|EBA04238.1| adenine phosphoribosyltransferase [Rhodobacterales bacterium HTCC2150];	-0,872835139	-0,867431426	-0,868412925	-0,795201581	-0,867922176	1,8	DOWN
159	C606907.1	gb|AAV65745.2||insulin receptor tyrosine kinase [Schistosoma mansoni]; emb|CAD30260.1||putative insulin receptor [Echinococcus multilocularis]; ref|XP_972770.2||PREDICTED: similar to insulin receptor, partial [Tribolium castaneum]; ref|XP_001377572.1||PREDICTED: similar to insulin receptor [Monodelphis domestica];	-0,882218928	-0,853316367	-0,684421136	-1,184136939	-0,867767648	1,8	DOWN
160	C608771.1	gb|AAD52698.1|AF091537_1|very low density lipoprotein binding protein precursor [Schistosoma japonicum] gb|ACE06889.1| unknown [Schistosoma japonicum];	-0,865999646	-0,699591727	-1,036084899	-0,866689835	-0,866344741	1,8	DOWN
161	C611764.1	gb|AAX25077.2||SJCHGC03771 protein [Schistosoma japonicum]; gb|AAX26627.2||SJCHGC07255 protein [Schistosoma japonicum];	-0,920491589	-1,032852461	-0,748989203	-0,807975504	-0,864233547	1,8	DOWN
162	C605909.1	No match	-0,557973858	-0,652265494	-1,058304557	-1,067984364	-0,855285026	1,8	DOWN
163	C606347.1	gb|AAW25679.1||SJCHGC01491 protein [Schistosoma japonicum]; gb|AAP05894.1||similar to GenBank Accession Number AF183413 dolichyl-phosphate beta-glucosyltransferase in Homo sapiens [Schistosoma japonicum]; ref|XP_417093.2||PREDICTED: similar to dolichyl-phosphate beta-glucosyltransferase [Gallus gallus]; ref|XP_001496408.2||PREDICTED: similar to Dolichyl-phosphate beta-glucosyltransferase (DolP-glucosyltransferase) (Asparagine-linked glycosylation protein 5) [Equus caballus];	-0,958663426	-0,949340541	-0,758881104	-0,662591204	-0,854110823	1,8	DOWN
164	C714516.1	gb|AAW25130.1||SJCHGC02831 protein [Schistosoma japonicum]; gb|AAY32924.1||TGF-beta receptor interacting protein 1 [Clonorchis sinensis]; ref|NP_001087664.1||eukaryotic translation initiation factor 3, subunit 2 beta [Xenopus laevis] gb|AAH81058.1| Eif3s2 protein [Xenopus laevis]; gb|AAI27353.1||Eif3s2 protein [Xenopus tropicalis];	-0,578253504	-0,64018225	-1,091136156	-1,067195847	-0,853689049	1,8	DOWN
165	C601947.1	gb|AAX26526.2||SJCHGC09040 protein [Schistosoma japonicum]; gb|AAF64527.1|AF254148_1|PUR-alpha-like protein [Schistosoma mansoni]; gb|AAW26889.1||SJCHGC06753 protein [Schistosoma japonicum]; gb|EDW50911.1||GM26800 [Drosophila sechellia];	-0,658073909	-0,80505112	-0,90112043	-1,009823433	-0,853085775	1,8	DOWN
166	C606089.1	No match	-0,860645166	-0,980944742	-0,84506953	-0,811201329	-0,852857348	1,8	DOWN
167	C602647.1	No match	-0,782837078	-0,538068725	-1,031210498	-0,91983169	-0,851334384	1,8	DOWN

168	C602253.1	gb|AAW26219.1||SJCHGC02185 protein [Schistosoma japonicum]; gb|AAP06082.1||similar to XM_040577 CGI-12 protein in Homo sapiens [Schistosoma japonicum]; ref|XP_001951989.1||PREDICTED: similar to CG5047 CG5047-PA [Acyrthosiphon pisum]; ref|NP_001002615.1||MTERF domain containing 1 [Danio rerio] gb|AAH75975.1| MTERF domain containing 1 [Danio rerio];	-0,89554423	-0,852454534	-0,777423973	-0,844495646	-0,84847509	1,8	DOWN
169	C601216.1	No match	-0,996591184	-0,8432526	-0,840867176	-0,84998355	-0,846618075	1,8	DOWN
170	C608615.1	tpe|CAJ00235.1||TPA: endonuclease-reverse transcriptase [Schistosoma mansoni];	-0,72111761	0,211029298	-0,966389971	-1,175164237	-0,843753791	1,8	DOWN
171	C601960.1	gb|AAW26659.1||SJCHGC01731 protein [Schistosoma japonicum]; gb|EDW27311.1||GL21220 [Drosophila persimilis]; gb|EDW13480.1||GI18231 [Drosophila mojavensis]; ref|XP_001356252.1||GA20298-PA [Drosophila pseudoobscura] gb|EAL33315.1| GA20298-PA [Drosophila pseudoobscura];	-0,839927769	-0,757668827	-0,839364833	-0,894513514	-0,839646301	1,8	DOWN
172	C610618.1	No match	-0,317768923	-0,447728424	-1,423058994	-1,220634631	-0,834181528	1,8	DOWN
173	C609011.1	gb|AAP06411.1||SJCHGC06675 protein [Schistosoma japonicum]; gb|ABS45569.1||QM protein [Marsupenaeus japonicus]; gb|ABU41039.1||putative QM protein [Lepeophtheirus salmonis]; gb|ABY61391.1||QM protein [Polyrhachis vicina];	-0,816637617	-0,77524753	-0,905174126	-0,848830014	-0,832733816	1,8	DOWN
174	C605584.1	ref|XP_001380983.1||PREDICTED: similar to PIG-A protein [Monodelphis domestica]; ref|XP_001138368.1||PREDICTED: phosphatidylinositol N-acetylglucosaminyltransferase subunit A isoform 2 [Pan troglodytes]; ref|XP_520945.2||PREDICTED: phosphatidylinositol N-acetylglucosaminyltransferase subunit A isoform 6 [Pan troglodytes]; dbj|BAG37537.1||unnamed protein product [Homo sapiens];	-0,692307254	-0,709185289	-1,01394869	-0,945697792	-0,827441541	1,8	DOWN
175	C604320.1	No match	-0,77632374	-0,770488883	-0,888400923	-0,876530672	-0,826427206	1,8	DOWN
176	C601338.1	No match	-0,84377598	-0,80638567	-0,70012312	-0,858694278	-0,825080825	1,8	DOWN
177	C610092.1	gb|AAW27429.1||SJCHGC02676 protein [Schistosoma japonicum];	-0,778886204	-0,771250954	-0,950312752	-0,871163133	-0,825024669	1,8	DOWN
178	C603197.1	ref|NP_031888.2||discs large homolog 1 [Mus musculus] ref|XP_001479222.1| PREDICTED: similar to Discs, large homolog 1 (Drosophila) isoform 2 [Mus musculus] gb|AAH57118.1| Discs, large homolog 1 (Drosophila) [Mus musculus] gb|EDK97752.1| discs, large homolog 1 (Drosophila), isoform CRA_a [Mus musculus]; ref|XP_001500922.1||PREDICTED: discs, large homolog 1 (Drosophila) [Equus caballus]; gb|EDM11461.1||discs, large homolog 1 (Drosophila), isoform CRA_e [Rattus norvegicus]; gb|EDM11460.1||discs, large homolog 1 (Drosophila), isoform CRA_d [Rattus norvegicus];	-0,757837551	-0,66753714	-0,889984981	-1,181235368	-0,823911266	1,8	DOWN
179	C612334.1	No match	-0,965379167	-0,831712429	-0,813830635	-0,728685345	-0,822771532	1,8	DOWN
180	C603446.1	No match	-0,909316419	-1,041414795	-0,734912801	-0,470899793	-0,82211461	1,8	DOWN
181	C612595.1	No match	-0,727654773	-0,732236981	-0,966693704	-0,909826736	-0,821031859	1,8	DOWN
182	C604281.1	gb|AAW25623.1||SJCHGC01247 protein [Schistosoma japonicum]; ref|NP_001123800.1||hypothetical protein LOC100170551 [Xenopus (Silurana) tropicalis] gb|AAI67594.1| Unknown (protein for MGC:184902) [Xenopus tropicalis]; gb|EDW17245.1||GI16794 [Drosophila mojavensis]; gb|EDV38789.1||GF24975 [Drosophila ananassae];	-0,776176835	-0,68186374	-0,862130524	-0,86311472	-0,81915368	1,8	DOWN
183	C611102.1	No match	-0,838579458	-0,864883997	-0,798589639	-0,355500651	-0,818584549	1,8	DOWN

184	C608994.1	gb|AAX30391.1||SJCHGC03240 protein [Schistosoma japonicum];	-0,88597884	-1,043504616	-0,691833551	-0,750125357	-0,818052099	1,8	DOWN
185	C608093.1	ref|NP_001106670.1||nascent polypeptide-associated complex alpha subunit isoform a [Mus musculus]; gb|EDL24536.1||mCG17022, isoform CRA_a [Mus musculus]; sp|P70670|NACAM_MOUSE|Nascent polypeptide-associated complex subunit alpha, muscle-specific form (Alpha-NAC, muscle-specific form) gb|AAB18732.1| alpha-NAC, muscle-specific form gp220 gb|AAB18734.1| alpha-NAC, muscle-specific form gp220 [Mus musculus]; ref|XP_001115411.1||PREDICTED: similar to Nascent polypeptide-associated complex alpha subunit, muscle-specific form (Alpha-NAC, muscle-specific form) [Macaca mulatta];	-0,261623737	-0,787636519	-0,870716555	-0,84603105	-0,816833785	1,8	DOWN
186	C611222.1	gb|AAI57611.1||Unknown (protein for IMAGE:8857551) [Xenopus tropicalis]; gb|AAI54786.1||Zgc:64155 [Danio rerio]; gb|AAI52670.1||Zgc:64155 protein [Danio rerio]; ref|NP_956343.2||zinc finger, DHHC domain containing 4 [Danio rerio];	-0,845125075	-0,819579631	-0,813420013	-0,769800764	-0,816499822	1,8	DOWN
187	C610843.1	No match	-0,542332694	-0,747242544	-0,882841918	-1,104451171	-0,815042231	1,8	DOWN
188	C609637.1	gb|AAW27050.1||SJCHGC05369 protein [Schistosoma japonicum];	-0,842698546	-0,931944845	-0,786777828	-0,726319631	-0,814738187	1,8	DOWN
189	C610204.1	No match	-0,637120811	-0,722665889	-0,934705164	-0,89747376	-0,810069825	1,8	DOWN
190	C603465.1	No match	-0,783159065	-0,535842059	-0,83973335	-0,835866162	-0,809512614	1,8	DOWN
191	C609020.1	gb|AAX24535.2||SJCHGC07456 protein [Schistosoma japonicum];	-0,716612808	-0,923095116	-0,801609055	-0,817081901	-0,809345478	1,8	DOWN
192	C605069.1	gb|AAW25441.1||SJCHGC06472 protein [Schistosoma japonicum]; ref|XP_553715.3||AGAP009835-PA [Anopheles gambiae str. PEST] gb|EAL39215.3| AGAP009835-PA [Anopheles gambiae str. PEST]; gb|EDW77669.1||GK24436 [Drosophila willistoni]; ref|XP_001355895.1||GA19445-PA [Drosophila pseudoobscura] gb|EAL32954.1| GA19445-PA [Drosophila pseudoobscura];	-0,815194722	-0,67350664	-0,801463483	-0,826936061	-0,808329103	1,8	DOWN
193	C611089.1	gb|AAW26023.1||SJCHGC09218 protein [Schistosoma japonicum];	-0,774177282	-0,868619134	-0,788991913	-0,815454476	-0,802223195	1,7	DOWN
194	C607480.1	gb|AAW25563.1||SJCHGC04928 protein [Schistosoma japonicum];	-0,849169983	-0,821471523	-0,756257802	-0,781632119	-0,801551821	1,7	DOWN
195	C607654.1	gb|AAX27329.2||SJCHGC00542 protein [Schistosoma japonicum]; gb|AAP06056.1||hypothetical protein [Schistosoma japonicum];	-0,708210437	-0,815522272	-0,834752181	-0,7872582	-0,801390236	1,7	DOWN
196	C607067.1	gb|AAW27124.1||SJCHGC03490 protein [Schistosoma japonicum]; gb|AAX25376.2||SJCHGC08502 protein [Schistosoma japonicum];	-0,75197777	-0,909864352	-0,84866551	-0,752361623	-0,800513567	1,7	DOWN
197	C610245.1	No match	-0,638100089	-0,687000126	-1,034358487	-0,907854303	-0,797427215	1,7	DOWN
198	C711918.1	gb|AAX26453.2||SJCHGC06422 protein [Schistosoma japonicum]; gb|AAX26480.2||SJCHGC07601 protein [Schistosoma japonicum]; ref|XP_792209.2||PREDICTED: hypothetical protein [Strongylocentrotus purpuratus] ref|XP_001195941.1| PREDICTED: hypothetical protein [Strongylocentrotus purpuratus]; ref|NP_001026408.1||acyl-CoA synthetase long-chain family member 5 [Gallus gallus] emb|CAG32476.1| hypothetical protein [Gallus gallus];	-0,738388638	-0,026191026	-0,851801746	-0,858173356	-0,795095192	1,7	DOWN

199	C608103.1	gb|AAW25842.1||SJCHGC09285 protein [Schistosoma japonicum]; ref|NP_001086484.1||ribosomal protein L19 [Xenopus laevis] gb|AAH75206.1| Rpl19-prov protein [Xenopus laevis]; gb|ABN14900.1||ribosomal protein L19 [Taenia asiatica]; emb|CAD91441.1||ribosomal protein L19 [Crassostrea gigas];	-0,603753059	-0,728481123	-0,964098701	-0,859624361	-0,794052742	1,7	DOWN
200	C605075.1	No match	-0,860879907	-0,66184195	-0,912075216	-0,726455654	-0,793667781	1,7	DOWN
201	C608919.1	No match	-0,834442641	-1,00138371	-0,751520498	-0,463861182	-0,79298157	1,7	DOWN
202	C612498.1	gb|AAX27118.2||SJCHGC07833 protein [Schistosoma japonicum];	-1,057517366	-0,995591019	-0,589710526	-0,354863342	-0,792650773	1,7	DOWN
203	C717897.1	ref|XP_556087.3||AGAP005449-PA [Anopheles gambiae str. PEST] gb|EAL39826.3| AGAP005449-PA [Anopheles gambiae str. PEST]; ref|NP_001006802.1||Cas-Br-M (murine) ecotropic retroviral transforming sequence b [Xenopus tropicalis] sp|Q6DFR2|CBLB_XENTR E3 ubiquitin-protein ligase CBL-B (Signal transduction protein CBL-B) (SH3-binding protein CBL-B) gb|AAH76671.1| Cas-Br-M (murine) ecotropic retroviral transforming sequence b [Xenopus tropicalis]; ref|XP_556088.3||AGAP005449-PB [Anopheles gambiae str. PEST] gb|EAL39827.3| AGAP005449-PB [Anopheles gambiae str. PEST]; ref|NP_001084790.1||hypothetical protein LOC431829 [Xenopus laevis] sp|Q6NRE7|CBLBB_XENLA E3 ubiquitin-protein ligase CBL-B-B (Signal transduction protein CBL-B-B) (SH3-binding protein CBL-B-B) gb|AAH70806.1| MGC83879 protein [Xenopus laevis];	-0,510337189	-0,503928543	-1,190459387	-1,072349258	-0,791343224	1,7	DOWN
204	C603037.1	gb|AAX25383.2||SJCHGC05029 protein [Schistosoma japonicum];	-0,504740954	-0,47458321	-1,076775187	-1,13567746	-0,790758071	1,7	DOWN
205	C601160.1	No match	-0,638987112	-0,655827375	-1,056395931	-0,924794419	-0,790310897	1,7	DOWN
206	C606357.1	ref|XP_643550.1||hypothetical protein DDBDRAFT_0167213 [Dictyostelium discoideum AX4] gb|AAO53180.1| hypothetical protein [Dictyostelium discoideum] gb|EAL69515.1| hypothetical protein DDBDRAFT_0167213 [Dictyostelium discoideum AX4];	-0,70967841	-0,660618964	-1,146623724	-0,870381902	-0,790030156	1,7	DOWN
207	C600716.1	gb|ABB51559.1||CD36-like class B scavenger receptor [Schistosoma mansoni]; gb|AAW26454.1||SJCHGC06304 protein [Schistosoma japonicum]; ref|NP_001095623.1||scavenger receptor class B, member 2 [Bos taurus] gb|AAI49936.1| SCARB2 protein [Bos taurus]; ref|XP_001376794.1||PREDICTED: hypothetical protein [Monodelphis domestica];	-0,952223138	-0,853703638	-0,602883501	-0,726231966	-0,789967802	1,7	DOWN
208	C601151.1	gb|AAX27845.2||SJCHGC05385 protein [Schistosoma japonicum]; ref|XP_001337123.1||PREDICTED: similar to Abcf2 protein [Danio rerio]; gb|AAH66505.1||Abcf2 protein [Danio rerio]; ref|NP_958472.1||ATP-binding cassette, sub-family F (GCN20), member 2 [Danio rerio] gb|AAH47181.1| ATP-binding cassette, sub-family F (GCN20), member 2 [Danio rerio] gb|AAI65771.1| Abcf2 protein [synthetic construct];	-0,858423924	-0,65485496	-0,750406179	-0,828666012	-0,789536096	1,7	DOWN
209	C610602.1	No match	-0,732482444	-0,748617449	-0,826503279	-1,008863343	-0,787560364	1,7	DOWN

210	C609479.1	ref|XP_001378737.1||PREDICTED: similar to protein phosphatase 2A 65 kDa regulatory subunit, alpha [Monodelphis domestica]; ref|NP_001080135.1||protein phosphatase 2 (formerly 2A), regulatory subunit A (PR 65), alpha isoform [Xenopus laevis] gb|AAH46723.1| Ppp2r1a-prov protein [Xenopus laevis]; ref|NP_989405.1||hypothetical protein LOC395042 [Xenopus tropicalis] gb|AAH64863.1| Hypothetical protein MGC76072 [Xenopus tropicalis] emb|CAJ81356.1| protein phosphatase 2 (formerly 2A), regulatory subunit A (PR 65), alpha isoform [Xenopus tropicalis]; dbj|BAF83282.1||unnamed protein product [Homo sapiens];	-0,846092041	-0,833071399	-0,739006652	-0,680539917	-0,786039026	1,7	DOWN
211	C609859.1	gb|AAW25614.1||SJCHGC07048 protein [Schistosoma japonicum]; ref|XP_001657058.1||neurobeachin [Aedes aegypti] gb|EAT45124.1| neurobeachin [Aedes aegypti]; ref|XP_001649459.1||neurobeachin [Aedes aegypti] gb|EAT33014.1| neurobeachin [Aedes aegypti]; ref|XP_311142.4||AGAP000017-PA [Anopheles gambiae str. PEST] gb|EAA06488.4| AGAP000017-PA [Anopheles gambiae str. PEST];	-0,946178343	-0,779680644	-0,754771759	-0,788188633	-0,783934639	1,7	DOWN
212	C602080.1	gb|AAX28101.2||SJCHGC06578 protein [Schistosoma japonicum]; gb|AAP06171.1||hypothetical protein [Schistosoma japonicum]; gb|ACE06837.1||unknown [Schistosoma japonicum];	-0,649687901	-0,651525672	-0,935093673	-0,91040697	-0,780966321	1,7	DOWN
213	C608256.1	No match	-0,69528818	-0,831749464	-0,728911639	-0,863652392	-0,780330552	1,7	DOWN
214	C600229.1	gb|AAA96714.1||ATPase; gb|ABS19816.1||sarco/endoplasmic reticulum calcium ATPase isoform B [Pinctada fucata]; gb|ABS19817.1||sarco/endoplasmic reticulum calcium ATPase isoform C [Pinctada fucata]; gb|ABS19815.1||sarco/endoplasmic reticulum calcium ATPase isoform A [Pinctada fucata];	-0,649961984	-0,680315025	-1,039461292	-0,879807606	-0,780061316	1,7	DOWN
215	C604563.1	gb|AAW24708.1||SJCHGC01209 protein [Schistosoma japonicum]; gb|AAW27547.1||SJCHGC06637 protein [Schistosoma japonicum]; gb|AAW25408.1||SJCHGC02061 protein [Schistosoma japonicum]; gb|ACE06868.1||unknown [Schistosoma japonicum];	-0,640172599	-0,676035392	-1,013598003	-0,881356281	-0,778695837	1,7	DOWN
216	C601272.1	No match	-0,849517234	-0,740802585	-0,816310466	-0,695177704	-0,778556526	1,7	DOWN
217	C602198.1	No match	-0,934200577	-0,869648271	-0,686792303	-0,619563739	-0,778220287	1,7	DOWN
218	C603876.1	emb|CAK26791.1||PrA2 protein [Schistosoma mansoni]; emb|CAK26789.1||PrB [Schistosoma mansoni];	-0,568862768	-0,573020265	-1,141322578	-0,982719759	-0,777870012	1,7	DOWN
219	C607463.1	gb|AAW27114.1||SJCHGC06129 protein [Schistosoma japonicum];	-0,69951889	-0,695868738	-0,902467956	-0,849380299	-0,774449595	1,7	DOWN
220	C606724.1	No match	-0,648531755	-0,714910306	-0,832464012	-0,874113638	-0,773687159	1,7	DOWN

221	C602476.1	gb|AAW26315.1||SJCHGC05429 protein [Schistosoma japonicum]; ref|NP_001006327.1||mediator complex subunit 20 [Gallus gallus] sp|Q5ZKY9.1|MED20_CHICK Mediator of RNA polymerase II transcription subunit 20 (Mediator complex subunit 20) (TRF-proximal protein homolog) emb|CAG31604.1| hypothetical protein [Gallus gallus]; ref|NP_001096763.1||mediator complex subunit 20 [Bos taurus] gb|AAI46259.1| MED20 protein [Bos taurus]; ref|NP_001013196.1||mediator complex subunit 20 [Rattus norvegicus] sp|Q5XIE9.1|MED20_RAT Mediator of RNA polymerase II transcription subunit 20 (Mediator complex subunit 20) (TRF-proximal protein homolog) gb|AAH83734.1| Mediator complex subunit 20 [Rattus norvegicus] gb|EDM18891.1| rCG43485, isoform CRA_b [Rattus norvegicus];	-0,782719552	-0,829813669	-0,753065834	-0,759275026	-0,770997289	1,7	DOWN
222	C610914.1	gb|AAX27546.2||SJCHGC02390 protein [Schistosoma japonicum]; gb|AAP06359.1||hypothetical protein [Schistosoma japonicum]; gb|AAX27010.1||unknown [Schistosoma japonicum] gb|AAX30131.1| SJCHGC00333 protein [Schistosoma japonicum]; gb|AAX27020.1||unknown [Schistosoma japonicum];	-1,064281434	-0,835009792	-0,676009745	-0,703181225	-0,769095509	1,7	DOWN
223	C608351.1	sp|P42637|TPM1_SCHMA|Tropomyosin-1 (TMI) (Polypeptide 49) gb|AAA03011.1| tropomyosin; sp|Q26503|TPM_SCHHA|Tropomyosin gb|AAA88530.1| 'Schistosoma haematobium tropomyosin'; gb|AAS66088.1||tropomyosin [Orientobilharzia turkestanicum]; gb|ACE06925.1||unknown [Schistosoma japonicum];	-0,573756086	-0,574112294	-0,99721354	-0,961491611	-0,767801953	1,7	DOWN
224	C605114.1	dbj|BAE40671.1||unnamed protein product [Mus musculus]; ref|NP_081086.1||MutL protein homolog 1 [Mus musculus] gb|AAH21815.1| MutL homolog 1 (E. coli) [Mus musculus]; sp|Q9JK91|MLH1_MOUSE|DNA mismatch repair protein Mlh1 (MutL protein homolog 1) gb|AAF64514.1|AF250844_1 MutL homolog 1 protein [Mus musculus]; ref|XP_859198.1||PREDICTED: similar to MutL protein homolog 1 isoform 3 [Canis familiaris];	-1,039184231	-0,877318359	-0,653143083	-0,648201383	-0,765230721	1,7	DOWN
225	C603116.1	gb|AAP06391.1||similar to NM_001414 eukaryotic translation initiation factor 2B, subunit 1 (alpha, 26kD) in Homo sapiens [Schistosoma japonicum]; gb|AAW26067.1||SJCHGC06103 protein [Schistosoma japonicum]; ref|XP_001927637.1||PREDICTED: similar to Translation initiation factor eIF-2B subunit alpha (eIF-2B GDP-GTP exchange factor subunit alpha) [Sus scrofa]; ref|NP_001405.1||eukaryotic translation initiation factor 2B, subunit 1 alpha, 26kDa [Homo sapiens] ref|XP_522583.2| PREDICTED: eukaryotic translation initiation factor 2B, subunit 1 alpha, 26kDa [Pan troglodytes] sp|Q5RAR0.2|EI2BA_PONAB Translation initiation factor eIF-2B subunit alpha (eIF-2B GDP-GTP exchange factor subunit alpha) sp|Q14232|EI2BA_HUMAN Translation initiation factor eIF-2B subunit alpha (eIF-2B GDP-GTP exchange factor subunit alpha) emb|CAA64950.1| eIF-2B [Homo sapiens] emb|CAG33112.1| EIF2B1 [Homo sapiens] gb|AAI03764.1| Eukaryotic translation initiation factor 2B, subunit 1 alpha, 26kDa [Homo sapiens] gb|AAI04189.1| Eukaryotic translation initiation factor 2B, subunit 1 alpha, 26kDa [Homo sapiens] gb|AAI04190.1| Eukaryotic translatior	-0,787811455	-0,812882243	-0,742389903	-0,718730012	-0,765100679	1,7	DOWN

226	C602958.1	gb|AAC36355.1||apoferritin-2 [Schistosoma japonicum]; gb|ACE06912.1||unknown [Schistosoma japonicum]; gb|ABU49726.1||ferritin [Solanum tuberosum]; emb|CAH05075.1||ferritin [Conyza canadensis];	-0,658754457	-0,721122253	-0,809017003	-0,840189267	-0,765069628	1,7	DOWN
227	C600599.1	gb|AAW27499.1||SJCHGC06360 protein [Schistosoma japonicum]; ref|XP_309798.4||AGAP010894-PA [Anopheles gambiae str. PEST] gb|EAA05506.5| AGAP010894-PA [Anopheles gambiae str. PEST]; ref|XP_001599499.1||PREDICTED: similar to signal recognition particle receptor alpha subunit [Nasonia vitripennis]; ref|XP_395013.2||PREDICTED: similar to GTP-binding protein CG2522-PA isoform 1 [Apis mellifera];	-0,783119406	-0,859301392	-0,742051614	-0,637529616	-0,76258551	1,7	DOWN
228	C606929.1	No match	-0,669365766	-0,682582125	-0,863784252	-0,830888975	-0,75673555	1,7	DOWN
229	C603551.1	gb|AAW27453.1||SJCHGC09200 protein [Schistosoma japonicum]; gb|AAW24780.1||SJCHGC06442 protein [Schistosoma japonicum]; gb|AAX26772.2||SJCHGC09234 protein [Schistosoma japonicum]; gb|AAW27224.1||SJCHGC06580 protein [Schistosoma japonicum];	-0,417969054	-0,430441656	-1,081686037	-1,109633298	-0,756063847	1,7	DOWN
230	C605350.1	gb|AAW26983.1||SJCHGC09421 protein [Schistosoma japonicum]; ref|XP_001369190.1||PREDICTED: similar to Signal recognition particle 68 kDa protein (SRP68) [Monodelphis domestica]; ref|NP_001120316.1||hypothetical protein LOC100145378 [Xenopus tropicalis] ref|XP_001922310.1| PREDICTED: signal recognition particle 68 [Danio rerio] gb|AAI52636.1| Srp68 protein [Danio rerio] gb|AAI60941.1| Unknown (protein for MGC:180907) [Xenopus tropicalis]; gb|AAT68165.1||68kDa signal recognition particle [Danio rerio];	-0,719225751	-0,712130999	-0,791756354	-1,163837713	-0,755491053	1,7	DOWN
231	C603060.1	No match	-0,602475662	-0,648307297	-0,895837295	-0,861870581	-0,755088939	1,7	DOWN
232	C607110.1	ref|XP_001637772.1||predicted protein [Nematostella vectensis] gb|EDO45709.1| predicted protein [Nematostella vectensis]; ref|NP_061377.2||phosphatidylinositol glycan anchor biosynthesis, class B [Mus musculus] sp|Q9JJQ0|PIGB_MOUSE GPI mannosyltransferase 3 (GPI mannosyltransferase III) (GPI-MT-III) (Phosphatidylinositol-glycan biosynthesis class B protein) (PIG-B) dbj|BAE32195.1| unnamed protein product [Mus musculus]; dbj|BAA94827.1||Pig-b [Mus musculus] gb|EDL26282.1| phosphatidylinositol glycan anchor biosynthesis, class B, isoform CRA_b [Mus musculus]; gb|AAH52658.1||Pigb protein [Mus musculus];	-1,010944181	-1,059363301	-0,497356682	-0,44646123	-0,754150432	1,7	DOWN
233	C602218.1	gb|AAX25391.2||SJCHGC07955 protein [Schistosoma japonicum];	-0,821300428	-0,815211718	-0,657874375	-0,691406953	-0,753309336	1,7	DOWN

234	C606706.1	gb|AAW24618.1||SJCHGC07079 protein [Schistosoma japonicum]; ref|NP_001017874.1||hypothetical protein LOC550572 [Danio rerio] sp|Q561X0.1|TM188_DANRE Transmembrane protein 188 gb|AAH92973.1| Zgc:110674 [Danio rerio]; ref|NP_001099643.1||transmembrane protein 188 [Rattus norvegicus] gb|EDL87521.1| similar to CG8009-PA (predicted), isoform CRA_b [Rattus norvegicus]; ref|NP_001011315.1||hypothetical protein LOC496772 [Xenopus tropicalis] sp|Q5M8F7.1|TM188_XENTR Transmembrane protein 188 gb|AAH88044.1| Hypothetical LOC496772 [Xenopus tropicalis] emb|CAJ81789.1| novel protein [Xenopus tropicalis];	-0,894860769	-0,782930967	-0,720195944	-0,714535498	-0,751563456	1,7	DOWN
235	C611835.1	No match	-0,765399409	-0,814503376	-0,735604298	-0,65931614	-0,750501854	1,7	DOWN
236	C609457.1	No match	-0,363860839	-0,573340944	-0,999053683	-0,927451752	-0,750396348	1,7	DOWN
237	C611442.1	gb|AAW24724.1||SJCHGC03621 protein [Schistosoma japonicum]; ref|NP_001107356.1||hypothetical protein LOC100135181 [Xenopus tropicalis] gb|AAI57204.1| Unknown (protein for MGC:135899) [Xenopus tropicalis]; gb|EDV27133.1||hypothetical protein TRIADDRAFT_54778 [Trichoplax adhaerens]; ref|NP_001079638.1||hypothetical protein LOC379325 [Xenopus laevis] sp|Q7ZWY2.1|PIHD1_XENLA PIH1 domain-containing protein 1 gb|AAH46655.1| MGC52879 protein [Xenopus laevis] gb|AAI06294.1| MGC52879 protein [Xenopus laevis];	-0,753814824	-0,744574756	-0,911191895	-0,715907294	-0,74919479	1,7	DOWN
238	C601416.1	gb|ABA40919.1||SJCHGC09844 protein [Schistosoma japonicum]; gb|AAX30374.1||SJCHGC03136 protein [Schistosoma japonicum]; gb|AAX31002.2||SJCHGC09704 protein [Schistosoma japonicum];	-0,626914544	-0,64508488	-0,874621038	-0,850191552	-0,747638216	1,7	DOWN
239	C603360.1	gb|AAW26296.1||SJCHGC05033 protein [Schistosoma japonicum]; ref|NP_001025681.1||MGC97807 protein [Xenopus tropicalis] gb|AAH93470.1| MGC97807 protein [Xenopus tropicalis]; ref|NP_957030.1||hypothetical protein LOC393709 [Danio rerio] gb|AAH59520.1| Chromosome 20 open reading frame 20 (H. sapiens) [Danio rerio]; gb|AAQ97748.1||chromosome 20 open reading frame 20 [Danio rerio];	-0,662581005	-0,801439701	-0,774689296	-0,719835585	-0,747262441	1,7	DOWN
240	C606707.1	gb|AAP06324.1||similar to NM_016539 sir2-related protein type 6 in Homo sapiens [Schistosoma japonicum]; ref|NP_989353.1||sirtuin (silent mating type information regulation 2 homolog) 6 [Xenopus (Silurana) tropicalis] gb|AAH64193.1| Sirtuin (silent mating type information regulation 2 homolog) 6 [Xenopus tropicalis]; emb|CAG33481.1||SIRT6 [Homo sapiens]; ref|XP_001624617.1||predicted protein [Nematostella vectensis] gb|EDO32517.1| predicted protein [Nematostella vectensis];	-0,990459417	-0,889597397	-0,604521901	-0,601046431	-0,747059649	1,7	DOWN
241	C610981.1	No match	-0,89832866	-0,864982803	-0,629122787	-0,542288831	-0,747052795	1,7	DOWN
242	C610259.1	No match	-0,664381911	-0,69415158	-0,96743182	-0,799393029	-0,746772305	1,7	DOWN
243	C603229.1	gb|AAX25526.2||SJCHGC06794 protein [Schistosoma japonicum];	-0,661691161	-0,720287983	-0,820339508	-0,773228845	-0,746758414	1,7	DOWN
244	C605936.1	No match	-0,80155867	-0,752924723	-0,740388368	-0,683612438	-0,746656546	1,7	DOWN

245	C711705.1	ref|NP_001085191.1||hypothetical protein LOC432280 [Xenopus laevis] gb|AAH72276.1| MGC82410 protein [Xenopus laevis]; emb|CAG02724.1||unnamed protein product [Tetraodon nigroviridis]; ref|XP_001372829.1||PREDICTED: hypothetical protein [Monodelphis domestica];	-0,838852317	-0,724423108	-0,766484151	-0,715908107	-0,74545363	1,7	DOWN
246	C609950.1	No match	-0,559447253	-0,693964304	-0,808110152	-0,791691994	-0,742828149	1,7	DOWN
247	C608473.1	gb|AAX24393.2||SJCHGC08648 protein [Schistosoma japonicum];	-0,906132779	-0,835515475	-0,649778283	-0,642614975	-0,742646879	1,7	DOWN
248	C610954.1	gb|ACE06921.1||unknown [Schistosoma japonicum]; gb|AAG40801.1||Mf2 protein [Schistosoma japonicum]; gb|AAX30252.1||SJCHGC02425 protein [Schistosoma japonicum];	-0,453452143	-0,620446859	-0,864831078	-0,883406342	-0,742638969	1,7	DOWN
249	C612177.1	gb|AAX24517.2||SJCHGC05069 protein [Schistosoma japonicum]; gb|AAX24756.2||SJCHGC07014 protein [Schistosoma japonicum];	-0,724796428	-0,71267254	-0,760368574	-0,865025662	-0,742582501	1,7	DOWN
250	C603980.1	gb|AAX27122.2||SJCHGC07140 protein [Schistosoma japonicum]; ref|NP_808446.1||armadillo repeat containing 7 [Mus musculus] sp|Q3UJZ3.2|ARMC7_MOUSE Armadillo repeat-containing protein 7 gb|AAH23126.1| Armadillo repeat containing 7 [Mus musculus] dbj|BAC31232.1| unnamed protein product [Mus musculus] emb|CAM23019.1| novel protein [Mus musculus] gb|EDL34497.1| armadillo repeat containing 7, isoform CRA_c [Mus musculus]; dbj|BAE27011.1||unnamed protein product [Mus musculus]; ref|XP_001138859.1||PREDICTED: hypothetical protein [Pan troglodytes];	-0,838042392	-0,896791459	-0,646657732	-0,523219549	-0,742350062	1,7	DOWN
251	C604782.1	No match	-0,853540183	-1,05220495	-0,626871919	-0,605703187	-0,740206051	1,7	DOWN
252	C600304.1	emb|CAE51198.1||src tyrosine kinase [Schistosoma mansoni];	-0,604860846	-0,618620327	-0,95985719	-0,860149062	-0,739384695	1,7	DOWN
253	C603401.1	No match	-0,705646466	-0,774783366	-0,769988599	-0,664365464	-0,737817533	1,7	DOWN
254	C605174.1	gb|EDW07154.1||GI15022 [Drosophila mojavensis]; ref|XP_001866846.1||histidyl-tRNA synthetase [Culex pipiens quinquefasciatus] gb|EDS43994.1| histidyl-tRNA synthetase [Culex quinquefasciatus]; gb|EDW04530.1||GH23584 [Drosophila grimshawi]; gb|EDV46605.1||GG19169 [Drosophila erecta];	-0,724928045	-0,606349925	-0,781139544	-0,748849921	-0,736888983	1,7	DOWN
255	C609178.1	No match	-0,565497238	-0,5721339	-0,899883724	-0,955383707	-0,736008812	1,7	DOWN
256	C607906.1	gb|EDV22419.1||hypothetical protein TRIADDRAFT_59005 [Trichoplax adhaerens]; ref|XP_001648295.1||flotillin-2 [Aedes aegypti] gb|EAT44635.1| flotillin-2 [Aedes aegypti]; ref|XP_001601913.1||PREDICTED: similar to CG32593-PB [Nasonia vitripennis]; ref|XP_001121998.1||PREDICTED: similar to flotillin 2 CG32593-PB, isoform B [Apis mellifera];	-0,974862667	-0,824502919	-0,532761572	-0,646137794	-0,735320357	1,7	DOWN
257	C601025.1	gb|AAR89512.1||ankyrin-like protein [Schistosoma mansoni]; ref|XP_001055725.1||PREDICTED: similar to ankyrin repeat domain 15 [Rattus norvegicus]; ref|XP_001080106.1||PREDICTED: similar to ankyrin repeat domain 15 [Rattus norvegicus]; ref|NP_852069.4||KN motif and ankyrin repeat domains 1 [Mus musculus];	-0,84801001	-0,769111608	-0,655548562	-0,698791811	-0,73395171	1,7	DOWN
258	C605465.1	No match	-0,814918276	-0,705698174	-0,734789444	-0,730580864	-0,732685154	1,7	DOWN

259	C604714.1	ref|XP_001655563.1||paxillin, putative [Aedes aegypti] gb|EAT48671.1| paxillin, putative [Aedes aegypti]; ref|XP_001845508.1||paxillin [Culex pipiens quinquefasciatus] gb|EDS40541.1| paxillin [Culex quinquefasciatus]; ref|XP_316909.4||AGAP008532-PA [Anopheles gambiae str. PEST] gb|EAA12524.5| AGAP008532-PA [Anopheles gambiae str. PEST]; gb|EDW90406.1||GE12653 [Drosophila yakuba];	-0,982141172	-0,740881061	-0,69038629	-0,720560111	-0,730720586	1,7	DOWN
260	C611441.1	No match	-0,046832709	-0,487355414	-1,051355402	-0,972086096	-0,729720755	1,7	DOWN
261	C606274.1	ref|XP_001197710.1||PREDICTED: similar to KIAA0268 [Strongylocentrotus purpuratus] ref|XP_001192525.1| PREDICTED: similar to KIAA0268 [Strongylocentrotus purpuratus]; ref|XP_317566.4||AGAP007919-PA [Anopheles gambiae str. PEST] gb|EAA12907.4| AGAP007919-PA [Anopheles gambiae str. PEST]; ref|XP_968468.2||PREDICTED: similar to Transport and Golgi organization 1 CG11098-PA [Tribolium castaneum]; ref|XP_001376049.1||PREDICTED: similar to C219-reactive peptide (FLJ39207) [Monodelphis domestica];	-0,734876244	-0,724498054	-0,760233264	-0,67147708	-0,729687149	1,7	DOWN
262	C607204.1	ref|NP_989228.1||thyroid hormone receptor interactor 13 [Xenopus tropicalis] gb|AAH63217.1| Thyroid hormone receptor interactor 13 [Xenopus tropicalis]; ref|XP_785120.1||PREDICTED: similar to Thyroid hormone receptor interactor 13 isoform 1 [Strongylocentrotus purpuratus] ref|XP_001185736.1| PREDICTED: similar to Thyroid hormone receptor interactor 13 [Strongylocentrotus purpuratus]; ref|XP_418892.2||PREDICTED: similar to HPV16 E1 protein binding protein [Gallus gallus]; ref|XP_001203908.1||PREDICTED: similar to Thyroid hormone receptor interactor 13, partial [Strongylocentrotus purpuratus] ref|XP_795235.2| PREDICTED: similar to Thyroid hormone receptor interactor 13, partial [Strongylocentrotus purpuratus];	-1,199056783	-0,895957032	-0,562149156	-0,453184516	-0,729053094	1,7	DOWN
263	C601420.1	gb|AAW27593.1||SJCHGC09090 protein [Schistosoma japonicum]; ref|XP_001626060.1||predicted protein [Nematostella vectensis] gb|EDO33960.1| predicted protein [Nematostella vectensis]; gb|AAX43326.1||ribosomal protein S13 [synthetic construct]; ref|NP_001008.1||ribosomal protein S13 [Homo sapiens] ref|NP_080809.1| ribosomal protein S13 [Mus musculus] ref|NP_569116.1| ribosomal protein S13 [Rattus norvegicus] ref|NP_001001783.1| ribosomal protein S13 [Gallus gallus] ref|NP_001020513.1| ribosomal protein S13 [Bos taurus] ref|XP_508306.1| PREDICTED: hypothetical protein [Pan troglodytes] ref|XP_852037.1| PREDICTED: similar to ribosomal protein S13 isoform 1 [Canis familiaris] ref|XP_864902.1| PREDICTED: similar to ribosomal protein S13 isoform 2 [Canis familiaris] ref|XP_894950.1| PREDICTED: hypothetical protein [Mus musculus] ref|XP_913564.1| PREDICTED: hypothetical protein [Mus musculus] ref|XP_001086088.1| PREDICTED: similar to ribosomal protein S13 [Macaca mulatta] ref|XP_001053043.1| PREDICTED: similar to ribosomal protein S13 [Rattus norvegicus] ref|XP_001479810.1| PREDICTED: hypothetical protein [Mus musc	-0,584408147	-0,556984404	-0,890227194	-0,87143531	-0,727921729	1,7	DOWN
264	C606928.1	No match	-0,63011682	-0,560872979	-0,825421933	-0,852168191	-0,727769377	1,7	DOWN
265	C610100.1	No match	-0,43808925	-0,389809875	-1,017361655	-1,339640273	-0,727725453	1,7	DOWN

266	C607572.1	gb|AAX26584.2||SJCHGC03347 protein [Schistosoma japonicum]; ref|XP_001398362.1||hypothetical protein An17g01330 [Aspergillus niger] emb|CAK43029.1| unnamed protein product [Aspergillus niger]; emb|CAB40376.1||adenosine kinase [Zea mays]; gb|ABR25519.1||adenosine kinase 2 [Oryza sativa (indica cultivar-group)];	-0,709144491	-0,71735682	-0,737428071	-0,753154637	-0,727392446	1,7	DOWN
267	C602981.1	gb|AAW25159.1||SJCHGC05901 protein [Schistosoma japonicum]; gb|AAI58932.1||Unknown (protein for IMAGE:7534917) [Xenopus tropicalis]; ref|NP_001016176.1||hypothetical protein LOC548930 [Xenopus tropicalis] emb|CAJ82567.1| Novel protein containing CRAL/TRIO domain [Xenopus tropicalis]; ref|NP_001026025.1||chromosome 20 open reading frame 121 [Gallus gallus] emb|CAH65429.1| hypothetical protein [Gallus gallus];	-0,77982841	-0,87232879	-0,669439268	-0,636407509	-0,724633839	1,7	DOWN
268	C612311.1	gb|AAX25647.2||SJCHGC05300 protein [Schistosoma japonicum];	-0,589946851	-0,453624073	-0,859210248	-0,947659255	-0,72457855	1,7	DOWN
269	C605879.1	No match	-0,563219081	-0,647808062	-0,899035369	-0,801337709	-0,724572886	1,7	DOWN
270	C604622.1	No match	-0,53671302	-0,551942799	-0,896369227	-1,10999212	-0,724156013	1,7	DOWN
271	C600901.1	No match	-0,717943008	-0,779184578	-0,727807337	-0,528492703	-0,722875173	1,7	DOWN
272	C612459.1	gb|AAX30386.1||SJCHGC03195 protein [Schistosoma japonicum]; dbj|BAD22612.1||Immt-Es [Meriones unguiculatus]; dbj|BAD22611.1||Immt-Er [Meriones unguiculatus]; gb|EDK98954.1||inner membrane protein, mitochondrial, isoform CRA_a [Mus musculus];	-0,837831303	-0,756620548	-0,684133218	-0,685752407	-0,721186478	1,6	DOWN
273	C602449.1	No match	-0,229812829	-0,41142566	-1,029156948	-1,062846999	-0,720291304	1,6	DOWN
274	C611219.1	gb|AAA99798.1||Pro-His-rich protein;	-0,668769522	-0,842577957	-0,771773304	-0,582748417	-0,720271413	1,6	DOWN
275	C602251.1	No match	-0,617402544	-0,698537179	-0,74168698	-0,787856656	-0,72011208	1,6	DOWN
276	C605523.1	gb|AAW27171.1||SJCHGC09312 protein [Schistosoma japonicum]; ref|XP_001602921.1||PREDICTED: similar to retinoblastoma-binding protein 4 (rbbp4) [Nasonia vitripennis]; ref|XP_973479.1||PREDICTED: similar to retinoblastoma-binding protein 4 (rbbp4) [Tribolium castaneum]; ref|XP_624580.1||PREDICTED: similar to Chromatin assembly factor 1 subunit CG4236-PA [Apis mellifera];	-1,046637945	-0,931854087	-0,507724755	-0,364217745	-0,719789421	1,6	DOWN
277	C604536.1	No match	-0,664700553	-0,519232747	-0,926751985	-0,774272231	-0,719486392	1,6	DOWN
278	C606129.1	gb|AAW26537.1||SJCHGC05331 protein [Schistosoma japonicum]; gb|EDM02368.1||SGT1, suppressor of G2 allele of SKP1 (S. cerevisiae), isoform CRA_c [Rattus norvegicus]; gb|EDM02366.1||SGT1, suppressor of G2 allele of SKP1 (S. cerevisiae), isoform CRA_a [Rattus norvegicus]; ref|XP_214242.2||PREDICTED: similar to SGT1, suppressor of G2 allele of SKP1 [Rattus norvegicus] ref|XP_001073449.1| PREDICTED: similar to SGT1, suppressor of G2 allele of SKP1 [Rattus norvegicus] sp|B0BN85.1|SUGT1_RAT Suppressor of G2 allele of SKP1 homolog gb|EDM02367.1| SGT1, suppressor of G2 allele of SKP1 (S. cerevisiae), isoform CRA_b [Rattus norvegicus] gb|AAI58725.1| Sugt1 protein [Rattus norvegicus];	-0,533231982	-0,489956777	-0,903135429	-0,948921984	-0,718183706	1,6	DOWN
279	C607076.1	gb|AAX25389.2||SJCHGC05691 protein [Schistosoma japonicum];	-0,632023231	-0,786881103	-0,64763071	-1,087498906	-0,717255907	1,6	DOWN
280	C602153.1	gb|AAW25030.1||SJCHGC06604 protein [Schistosoma japonicum];	-0,818451603	-0,802970133	-0,631341733	-0,434762404	-0,717155933	1,6	DOWN

281	C610101.1	gb|AAW26064.1||SJCHGC06192 protein [Schistosoma japonicum]; ref|XP_001199439.1||PREDICTED: hypothetical protein [Strongylocentrotus purpuratus]; ref|XP_396596.1||PREDICTED: similar to CG9769-PA [Apis mellifera]; ref|XP_001659715.1||eukaryotic translation initiation factor 3f, eif3f [Aedes aegypti] gb|ABF18280.1| eukaryotic translation initiation factor 3 subunit 5 epsilon-like protein [Aedes aegypti] gb|EAT39069.1| eukaryotic translation initiation factor 3f, eif3f [Aedes aegypti];	-0,472645917	-0,522997741	-0,980822719	-0,910403175	-0,716700458	1,6	DOWN
282	C611337.1	No match	-0,393848196	-0,487555479	-1,012724212	-0,942770202	-0,715162841	1,6	DOWN
283	C610659.1	gb|AAX26112.2||SJCHGC03668 protein [Schistosoma japonicum]; ref|XP_001640751.1||predicted protein [Nematostella vectensis] gb|EDO48688.1| predicted protein [Nematostella vectensis]; ref|XP_001371170.1||PREDICTED: hypothetical protein [Monodelphis domestica]; gb|EDM04317.1||nucleolar protein family A, member 2 (predicted), isoform CRA_c [Rattus norvegicus] gb|AAI62010.1| Nola2 protein [Rattus norvegicus];	-0,301634961	-0,574004839	-0,853735794	-1,111418888	-0,713870317	1,6	DOWN
284	C603770.1	No match	-0,636526561	-0,689663238	-0,74630028	-0,736926105	-0,713294672	1,6	DOWN
285	C609856.1	No match	-0,771705609	-0,801279554	-0,65334683	-0,63045739	-0,71252622	1,6	DOWN
286	C608869.1	No match	-0,626777704	-0,177817293	-0,796465925	-0,83348933	-0,711621815	1,6	DOWN
287	C611909.1	No match	-0,835891082	-0,86741178	-0,587226232	-0,434691322	-0,711558657	1,6	DOWN
288	C600917.1	No match	-0,613752472	-0,588554171	-0,906085449	-0,807651461	-0,710701967	1,6	DOWN
289	C603382.1	No match	-0,784835136	-0,790313735	-0,634209348	-0,572949443	-0,709522242	1,6	DOWN
290	C606958.1	No match	-0,845998879	-0,68577134	-0,659650677	-0,732741389	-0,709256365	1,6	DOWN
291	C610841.1	No match	-0,488958488	-0,555846712	-0,893163095	-0,859331403	-0,707589058	1,6	DOWN
292	C612451.1	ref|NP_001085639.1||MGC82703 protein [Xenopus laevis] sp|Q6GPP7.1|TM209_XENLA Transmembrane protein 209 gb|AAH73065.1| MGC82703 protein [Xenopus laevis]; ref|XP_682843.3||PREDICTED: similar to transmembrane protein 209 [Danio rerio]; ref|NP_001086561.1||MGC83764 protein [Xenopus laevis] gb|AAH76809.1| MGC83764 protein [Xenopus laevis]; ref|XP_780705.1||PREDICTED: hypothetical protein [Strongylocentrotus purpuratus] ref|XP_001193783.1| PREDICTED: hypothetical protein [Strongylocentrotus purpuratus];	-0,765592677	-0,658113916	-0,645838216	-0,755321212	-0,706717564	1,6	DOWN
293	C608370.1	gb|AAW26144.1||SJCHGC02223 protein [Schistosoma japonicum]; gb|AAF73250.1|AF153977_1|eukaryotic translation initiation factor 5 [Schistosoma mansoni]; gb|EDV24103.1||hypothetical protein TRIADDRAFT_50382 [Trichoplax adhaerens]; ref|NP_955885.1||eukaryotic translation initiation factor 5 [Danio rerio] gb|AAH49502.1| Eukaryotic translation initiation factor 5 [Danio rerio] gb|AAH66614.1| Eukaryotic translation initiation factor 5 [Danio rerio] gb|AAS92648.1| eukaryotic translation initiation factor 5 [Danio rerio] emb|CAI21349.1| novel protein (zgc:77026) [Danio rerio];	-0,308841383	-0,338395654	-1,073657356	-1,086691705	-0,706026505	1,6	DOWN

294	C605237.1	sp|Q86QS6|AN32_SCHMA|Acidic leucine-rich nuclear phosphoprotein 32-related protein (ANP32/acidic nuclear phosphoprotein-like protein) gb|AAO21365.1| leucine-rich protein [Schistosoma mansoni]; gb|AAW25017.1||SJCHGC00366 protein [Schistosoma japonicum] gb|ABA40386.2| SJCHGC02024 protein [Schistosoma japonicum]; gb|AAW24968.1||unknown [Schistosoma japonicum]; gb|AAW24920.1||unknown [Schistosoma japonicum];	-0,508863356	-0,536249204	-0,968210492	-0,874930088	-0,705589646	1,6	DOWN
295	C603706.1	gb|ACE06846.1||unknown [Schistosoma japonicum]; gb|ABG21812.1||60S ribosomal protein L15-like protein [Schistosoma mansoni]; gb|AAP06105.1||similar to GenBank Accession Number X78167 ribosomal protein L15 in Rattus norvegicus [Schistosoma japonicum]; ref|XP_932648.1||PREDICTED: hypothetical protein isoform 1 [Homo sapiens] ref|XP_949197.1| PREDICTED: hypothetical protein isoform 4 [Homo sapiens] ref|XP_001725140.1| PREDICTED: hypothetical protein [Homo sapiens];	-0,873721955	-0,871843167	-0,537203866	-0,310260326	-0,704523517	1,6	DOWN
296	C606337.1	gb|AAW27674.1||SJCHGC04249 protein [Schistosoma japonicum]; gb|ABW23219.1||ribosomal protein rpl13a [Eurythoe complanata]; gb|ABX75375.1||60S ribosomal protein L13A [Lycosa singoriensis]; gb|ABZ04228.1||ribosomal protein rpl13a [Lineus viridis];	-0,324751489	-0,425272922	-1,007997354	-0,983419993	-0,704346458	1,6	DOWN
297	C611150.1	No match	-0,522660654	-0,360421018	-0,884171294	-0,90567964	-0,703415974	1,6	DOWN
298	C608712.1	gb|AAX26800.2||SJCHGC06570 protein [Schistosoma japonicum];	-0,743445726	-0,780182656	-0,663313708	-0,639127407	-0,703379717	1,6	DOWN
299	C609829.1	ref|XP_855370.1||PREDICTED: similar to Delta-aminolevulinic acid dehydratase (Porphobilinogen synthase) (ALADH) [Canis familiaris]; ref|XP_001101487.1||PREDICTED: similar to delta-aminolevulinic acid dehydratase isoform a isoform 2 [Macaca mulatta]; ref|XP_001101392.1||PREDICTED: similar to delta-aminolevulinic acid dehydratase isoform a isoform 1 [Macaca mulatta]; ref|XP_001101576.1||PREDICTED: similar to delta-aminolevulinic acid dehydratase isoform b isoform 3 [Macaca mulatta] dbj|BAE02020.1| unnamed protein product [Macaca fascicularis];	-0,800016276	-0,932101987	-0,606436244	-0,18524033	-0,70322626	1,6	DOWN
300	C602959.1	No match	-0,463931324	-0,543286672	-0,862108541	-0,917536963	-0,702697607	1,6	DOWN
301	C611397.1	gb|AAX27308.2||SJCHGC04233 protein [Schistosoma japonicum]; ref|XP_968299.1||PREDICTED: similar to N-glycosylase/DNA lyase [Tribolium castaneum]; ref|XP_001619921.1||hypothetical protein NEMVEDRAFT_v1g149802 [Nematostella vectensis] gb|EDO27821.1| predicted protein [Nematostella vectensis]; ref|XP_001375382.1||PREDICTED: similar to 8-oxoguanine DNA glycosylase [Monodelphis domestica];	-0,626500245	-0,898273118	-0,686444174	-0,718379777	-0,702411976	1,6	DOWN
302	C601498.1	gb|AAW26397.1||SJCHGC09094 protein [Schistosoma japonicum];	-0,723032251	-0,620019037	-0,72723921	-0,678789803	-0,700911027	1,6	DOWN
303	C609300.1	No match	-0,596499744	-0,683849615	-0,803881273	-0,716572524	-0,70021107	1,6	DOWN
304	C610858.1	No match	-0,593914772	-0,645075295	-0,849540735	-0,749499218	-0,697287257	1,6	DOWN

305	C603255.1	gb|AAW24485.1||SJCHGC06694 protein [Schistosoma japonicum]; gb|EDV21550.1||hypothetical protein TRIADDRAFT_59660 [Trichoplax adhaerens]; ref|NP_997968.1||eukaryotic translation initiation factor 4E binding protein 2 [Danio rerio] gb|AAH66546.1| Eukaryotic translation initiation factor 4E binding protein 2 [Danio rerio]; ref|NP_001028241.1||eukaryotic translation initiation factor 4E binding protein 2 [Rattus norvegicus] gb|AAI00639.1| Eukaryotic translation initiation factor 4E binding protein 2 [Rattus norvegicus] gb|EDL93024.1| eukaryotic translation initiation factor 4E binding protein 2, isoform CRA_b [Rattus norvegicus];	-0,683957941	-0,62570637	-0,72605246	-0,706759933	-0,695358937	1,6	DOWN
306	C608005.1	gb|EDV20694.1||hypothetical protein TRIADDRAFT_31577 [Trichoplax adhaerens]; gb|AAG60622.1|AF294916_1|phosphoinositide-dependent protein kinase I [Aplysia californica]; emb|CAG04372.1||unnamed protein product [Tetraodon nigroviridis]; ref|XP_786576.2||PREDICTED: similar to phosphoinositide dependent kinase-1 [Strongylocentrotus purpuratus] ref|XP_001189048.1| PREDICTED: similar to phosphoinositide dependent kinase-1 [Strongylocentrotus purpuratus];	-0,851657231	-1,219246167	-0,535862834	-0,479986656	-0,693760033	1,6	DOWN
307	C606376.1	gb|ABQ15152.1||nuclear coactivator 1 [Schistosoma mansoni]; emb|CAI59265.1||ski interacting protein [Echinococcus multilocularis]; ref|NP_001017145.1||SKI interacting protein [Xenopus tropicalis] emb|CAJ83628.1| SKI interacting protein [Xenopus tropicalis]; ref|NP_001002864.1||SNW domain containing 1 [Danio rerio] gb|AAT68034.1| ski-interacting protein [Danio rerio];	-0,652583479	-0,730036457	-0,735290866	-0,604064522	-0,691309968	1,6	DOWN
308	C605630.1	gb|AAX30712.2||SJCHGC07106 protein [Schistosoma japonicum];	-0,650478272	-0,790614323	-0,702215296	-0,67870379	-0,690459543	1,6	DOWN
309	C602841.1	No match	-0,397170657	-0,489879899	-0,950741548	-0,890614415	-0,690247157	1,6	DOWN
310	C608753.1	gb|AAX26899.2||SJCHGC03553 protein [Schistosoma japonicum];	-0,636756755	-0,510984288	-0,984669572	-0,743551366	-0,690154061	1,6	DOWN
311	C602787.1	tpe|CAJ00244.1||TPA: endonuclease-reverse transcriptase [Schistosoma mansoni];	-0,576099168	-0,542763447	-0,836036942	-0,803681975	-0,689890572	1,6	DOWN
312	C607827.1	gb|AAW26207.1||SJCHGC02796 protein [Schistosoma japonicum];	-0,931042224	-0,759750019	-0,619546993	-0,514956992	-0,689648506	1,6	DOWN
313	C603861.1	No match	-0,577624372	-0,694674628	-0,870573443	-0,680680273	-0,687677451	1,6	DOWN
314	C603772.1	ref|XP_624814.2||PREDICTED: similar to CG31717-PA, partial [Apis mellifera];	-0,807021369	-0,594576364	-0,780626999	-0,594035142	-0,687601682	1,6	DOWN
315	C606086.1	No match	-0,270300967	-0,797484976	-0,577661962	-1,089132358	-0,687573469	1,6	DOWN
316	C610127.1	No match	-0,651620426	-0,589177641	-0,733809493	-0,718372332	-0,684996379	1,6	DOWN
317	C606115.1	gb|AAW24653.1||SJCHGC09419 protein [Schistosoma japonicum];	-0,86178136	-0,774099179	-0,576175843	-0,595717809	-0,684908494	1,6	DOWN
318	C605917.1	No match	-0,958684866	-0,75972997	-0,564674083	-0,605796568	-0,682763269	1,6	DOWN
319	C608120.1	gb|AAX24759.2||SJCHGC04711 protein [Schistosoma japonicum];	-0,727790806	-0,711304548	-0,626138449	-0,635233488	-0,673269018	1,6	DOWN
320	C607128.1	ref|XP_001599458.1||PREDICTED: similar to CG32156-PC [Nasonia vitripennis];	-0,445589965	-0,513875141	-0,92937341	-0,831017604	-0,672446373	1,6	DOWN
321	C601174.1	No match	-0,534847548	-0,708149275	-0,743034083	-0,635169316	-0,671659296	1,6	DOWN
322	C612014.1	gb|AAW27065.1||SJCHGC05775 protein [Schistosoma japonicum];	-0,936998812	-0,762502308	-0,57584278	-0,313020462	-0,669172544	1,6	DOWN
323	C611710.1	No match	-0,79145199	-0,750096664	-0,534436704	-0,587371071	-0,668733868	1,6	DOWN

324	C608609.1	gb|AAP06199.1||similar to porin 31HM in human, skeletal muscle membranes [Schistosoma japonicum]; gb|AAI35546.1||Vdac1 protein [Xenopus tropicalis]; ref|NP_001016492.1||voltage-dependent anion channel 1 [Xenopus tropicalis] emb|CAJ83146.1| voltage-dependent anion channel 1 [Xenopus tropicalis] gb|AAI27349.1| Voltage-dependent anion channel 1 [Xenopus tropicalis]; ref|XP_780266.1||PREDICTED: similar to voltage-dependent anion channel 2 isoform 1 [Strongylocentrotus purpuratus] ref|XP_001177783.1| PREDICTED: similar to voltage-dependent anion channel 2 [Strongylocentrotus purpuratus];	-0,633414051	-0,635702992	-0,813047313	-0,701410458	-0,668556725	1,6	DOWN
325	C600948.1	gb|AAP06073.1||similar to NM_022756 hypothetical protein FLJ11730 in Homo sapiens; hypothetical protein FLJ11730 in Homo sapiens [Schistosoma japonicum]; gb|AAW25861.1||unknown [Schistosoma japonicum]; gb|AAW25871.1||SJCHGC00146 protein [Schistosoma japonicum]; gb|AAW25057.1||unknown [Schistosoma japonicum];	-0,874436554	-0,778277641	-0,493159849	-0,558794983	-0,668536312	1,6	DOWN
326	C606432.1	gb|AAW26531.1||unknown [Schistosoma japonicum]; gb|AAW25871.1||SJCHGC00146 protein [Schistosoma japonicum]; gb|AAW25213.1||unknown [Schistosoma japonicum]; gb|AAW27485.1||SJCHGC01610 protein [Schistosoma japonicum];	-0,376790577	-0,47313811	-0,87518738	-0,862179311	-0,667658711	1,6	DOWN
327	C603837.1	No match	-0,594139763	-0,616601024	-0,883348932	-0,714139527	-0,665370276	1,6	DOWN
328	C609703.1	gb|AAW25973.1||SJCHGC09415 protein [Schistosoma japonicum]; gb|EDX15624.1||GD15199 [Drosophila simulans]; gb|EDL84806.1||within bgcn homolog (Drosophila) (predicted), isoform CRA_a [Rattus norvegicus]; gb|AAI61580.1||Hypothetical protein LOC549028 [Xenopus tropicalis];	-0,604736865	-0,575415149	-0,725951027	-0,761558589	-0,665343946	1,6	DOWN
329	C607489.1	gb|AAW26907.1||SJCHGC09493 protein [Schistosoma japonicum]; gb|AAV97787.1||inhibitor of apoptosis protein [Schistosoma japonicum]; gb|ABZ82033.1||inhibitor of apoptosis protein [Clonorchis sinensis];	-0,573886758	-0,641180279	-0,759848251	-0,685379409	-0,663279844	1,6	DOWN
330	C607349.1	No match	-0,078431333	-0,419575431	-0,905719172	-0,918796108	-0,662647302	1,6	DOWN
331	C606245.1	No match	-0,320147211	-0,524349265	-0,818836219	-0,797683178	-0,661016222	1,6	DOWN
332	C607552.1	ref|XP_001812886.1||PREDICTED: similar to gmp synthase [Tribolium castaneum]; ref|XP_393336.2||PREDICTED: similar to GMP synthase [glutamine-hydrolyzing] (Glutamine amidotransferase) (GMP synthetase) [Apis mellifera]; ref|NP_001006556.1||guanine monphosphate synthetase [Gallus gallus] emb|CAG32582.1| hypothetical protein [Gallus gallus]; ref|XP_001512299.1||PREDICTED: similar to guanosine 5-monophosphate synthetase, partial [Ornithorhynchus anatinus];	-0,815821227	-0,788558387	-0,532933398	-0,529498142	-0,660745893	1,6	DOWN
333	C611310.1	No match	-0,724667236	-0,754664454	-0,430468702	-0,596770469	-0,660718853	1,6	DOWN
334	C607884.1	gb|AAW26173.1||SJCHGC06268 protein [Schistosoma japonicum]; gb|ABA40291.1||SJCHGC03923 protein [Schistosoma japonicum];	-0,709086505	-0,704758264	-0,616140603	-0,555201721	-0,660449434	1,6	DOWN
335	C601632.1	No match	-0,536789526	-0,47385286	-0,829994043	-0,781934968	-0,659362247	1,6	DOWN

336	C610640.1	ref|XP_001178408.1||PREDICTED: similar to translation initiation factor 2 gamma subunit [Strongylocentrotus purpuratus] ref|XP_787081.2| PREDICTED: similar to translation initiation factor 2 gamma subunit [Strongylocentrotus purpuratus]; ref|XP_001607783.1||PREDICTED: similar to eukaryotic translation initiation factor 2 gamma [Nasonia vitripennis]; emb|CAG29663.1||translation initiation factor 2 gamma subunit [Lithobius forficatus]; emb|CAG29668.1||translation initiation factor 2 gamma subunit [Lepismachilis sp. VK-2004];	-0,64121094	-0,674713459	-0,715847697	-0,643179358	-0,658946409	1,6	DOWN
337	C610615.1	gb|AAW26828.1||SJCHGC09446 protein [Schistosoma japonicum];	-0,73328078	-0,766812953	-0,584105742	-0,578510221	-0,658693261	1,6	DOWN
338	C604546.1	No match	-0,594511405	-0,46745921	-0,752253302	-0,722484381	-0,658497893	1,6	DOWN
339	C612582.1	gb|AAW25491.1||unknown [Schistosoma japonicum]; gb|ABN14902.1||60S ribosomal protein L10a [Taenia asiatica]; ref|XP_001891868.1||60S ribosomal protein L10a [Brugia malayi] gb|EDP39318.1| 60S ribosomal protein L10a, putative [Brugia malayi]; ref|NP_001120029.1||hypothetical protein LOC100144995 [Xenopus tropicalis] gb|AAI58336.1| Unknown (protein for MGC:185542) [Xenopus tropicalis];	-0,573944492	-0,471048957	-0,742388621	-0,901152193	-0,658166557	1,6	DOWN
340	C609005.1	No match	-0,632123236	-0,731908617	-0,664961468	-0,649981672	-0,65747157	1,6	DOWN
341	C605552.1	gb|AAX27943.2||SJCHGC05670 protein [Schistosoma japonicum];	-0,387863948	-0,606964198	-0,706323359	-0,793814357	-0,656643779	1,6	DOWN
342	C611119.1	gb|AAX24328.2||SJCHGC05542 protein [Schistosoma japonicum];	-0,633093413	-0,736771771	-0,680076905	-0,626601514	-0,656585159	1,6	DOWN
343	C605103.1	gb|AAW25211.1||SJCHGC02533 protein [Schistosoma japonicum]; ref|XP_001599894.1||PREDICTED: similar to past-1 [Nasonia vitripennis]; ref|XP_001674306.1||Hypothetical protein CBG18887 [Caenorhabditis briggsae AF16] emb|CAP36229.1| C. briggsae CBR-RME-1 protein [Caenorhabditis briggsae]; ref|XP_396463.2||PREDICTED: similar to Putative Achaete Scute Target 1 CG6148-PB, isoform B [Apis mellifera];	-1,070189129	-0,882764321	-0,429741143	-0,333911241	-0,656252732	1,6	DOWN
344	C610123.1	gb|AAW27501.1||SJCHGC01083 protein [Schistosoma japonicum]; gb|AAR16282.1||capping protein (actin filament) muscle Z-line, alpha 2 [Takifugu rubripes]; sp|P25229.1|CAZA1_XENLA|F-actin-capping protein subunit alpha-1 (Actin-binding protein chain A) (ABP-A) emb|CAA35948.1| nuclear actin-binding protein chain a [Xenopus laevis] prf||1602248A nuclear actin binding protein; gb|ABJ08879.1||capping protein (actin filament) muscle Z-line, alpha 2 [Ginglymostoma cirratum];	-0,519477361	-0,502649032	-0,797875545	-0,792212763	-0,655845062	1,6	DOWN
345	C611942.1	No match	-0,961287005	-0,239409836	-0,697601298	-0,611561422	-0,65458136	1,6	DOWN
346	C604799.1	No match	-0,699128257	-0,654301788	-0,259975556	-0,653695684	-0,653998736	1,6	DOWN
347	C601080.1	gb|AAW26780.1||SJCHGC02748 protein [Schistosoma japonicum];	-0,604572945	-0,801730841	-0,70026364	-0,512840558	-0,652418293	1,6	DOWN
348	C600648.1	gb|AAX30655.1||SJCHGC06537 protein [Schistosoma japonicum]; gb|ABW23211.1||ribosomal protein rps21 [Eurythoe complanata]; gb|ABK32077.1||ribosomal protein S21 [Branchiostoma belcheri]; sp|Q86CT3|RS21_BRABE|40S ribosomal protein S21 gb|AAP21828.1| ribosomal protein S21 [Branchiostoma belcheri tsingtaunese];	-0,167584648	-0,274040117	-1,100838128	-1,030572641	-0,652306379	1,6	DOWN
349	C612121.1	No match	-0,616324254	-0,643962358	-0,708755568	-0,660063734	-0,652013046	1,6	DOWN

350	C607061.1	gb|AAX26988.2||SJCHGC08857 protein [Schistosoma japonicum];	-0,795869646	-0,73502389	-0,506261497	-0,568047839	-0,651535865	1,6	DOWN
351	C605483.1	No match	-0,46217891	-0,643581582	-0,712506892	-0,658808358	-0,65119497	1,6	DOWN
352	C603801.1	No match	-0,809466094	-0,883565048	-0,463774844	-0,492113471	-0,650789783	1,6	DOWN
353	C611076.1	No match	-0,663857314	-0,641417711	-0,658740195	-0,632280032	-0,650078953	1,6	DOWN
354	C607705.1	gb|AAX25542.2||SJCHGC07903 protein [Schistosoma japonicum];	-0,643353947	-0,656718279	-0,614818761	-0,664250547	-0,650036113	1,6	DOWN
355	C611926.1	No match	-0,81829595	-0,7867082	-0,462181312	-0,511247793	-0,648977997	1,6	DOWN
356	C610202.1	ref|XP_001497523.2||PREDICTED: similar to serine/threonine kinase receptor associated protein [Equus caballus]; ref|XP_001159216.1||PREDICTED: serine/threonine kinase receptor associated protein [Pan troglodytes]; ref|XP_001159067.1||PREDICTED: serine/threonine kinase receptor associated protein [Pan troglodytes]; ref|XP_001159167.1||PREDICTED: serine/threonine kinase receptor associated protein [Pan troglodytes];	-0,596262182	-0,700574508	-0,526111483	-0,843607528	-0,648418345	1,6	DOWN
357	C602929.1	gb|AAW25379.1||SJCHGC09123 protein [Schistosoma japonicum]; gb|AAW26667.1||SJCHGC09411 protein [Schistosoma japonicum];	-0,398687602	-0,446501401	-0,878469137	-0,850045112	-0,648273257	1,6	DOWN
358	C612081.1	ref|XP_784978.2||PREDICTED: similar to mKIAA1219 protein [Strongylocentrotus purpuratus] ref|XP_001179040.1| PREDICTED: similar to mKIAA1219 protein [Strongylocentrotus purpuratus]; gb|EDW71187.1||GJ16196 [Drosophila virilis]; ref|NP_001096938.1||CG34408 CG34408-PB, isoform B [Drosophila melanogaster] gb|AAN09629.2| CG34408-PB, isoform B [Drosophila melanogaster]; gb|EDX16334.1||GD24698 [Drosophila simulans];	-0,49658552	-0,715435393	-0,669441844	-0,625632615	-0,64753723	1,6	DOWN
359	C600628.1	gb|AAF98445.1||Sj-Ts1 [Schistosoma japonicum]; gb|ACE06845.1||unknown [Schistosoma japonicum];	-0,060440999	-0,223966501	-1,065902177	-1,093566502	-0,644934339	1,6	DOWN
360	C612476.1	gb|ABC69927.1||STIP [Tetraodon nigroviridis]; emb|CAG08558.1||unnamed protein product [Tetraodon nigroviridis]; ref|NP_001073674.1||Sip1/TFIP11 interacting protein [Apis mellifera] gb|ABC69933.1| STIP [Apis mellifera]; gb|AAH17682.1||Tuftelin interacting protein 11 [Mus musculus];	-0,783898874	-0,727719457	-0,5613534	-0,272788287	-0,644536429	1,6	DOWN
361	C609825.1	gb|ABG21823.1||ribosomal protein L4-like protein [Schistosoma mansoni]; gb|AAW25794.1||SJCHGC06667 protein [Schistosoma japonicum]; gb|ABN14894.1||ribosomal protein L4 [Taenia asiatica]; gb|AAH63811.1||Ribosomal protein L4 [Rattus norvegicus] gb|AAH81801.1| Ribosomal protein L4 [Rattus norvegicus] gb|EDL95788.1| rCG58071, isoform CRA_a [Rattus norvegicus];	-0,255174852	-0,454944396	-0,833661141	-0,872183083	-0,644302769	1,6	DOWN
362	C610769.1	No match	-0,51305626	-0,544792946	-0,741394389	-0,779314114	-0,643093668	1,6	DOWN
363	C611067.1	No match	-0,714481162	-0,505978253	-0,638790213	-0,64697773	-0,642883972	1,6	DOWN
364	C707411.1	gb|AAW26906.1||SJCHGC00905 protein [Schistosoma japonicum]; ref|NP_001080498.1||excision repair cross-complementing rodent repair deficiency, complementation group 1 (includes overlapping antisense sequence) [Xenopus laevis] gb|AAH43824.1| Ercc1-prov protein [Xenopus laevis]; gb|AAW27225.1||SJCHGC04667 protein [Schistosoma japonicum]; ref|NP_001107393.1||hypothetical protein LOC100135224 [Xenopus tropicalis] gb|AAI57332.1| Unknown (protein for MGC:147821) [Xenopus tropicalis];	-0,641898072	-0,723440612	-0,610612014	-0,643021421	-0,642459747	1,6	DOWN

365	C611587.1	dbj|BAB28672.1||unnamed protein product [Mus musculus]; ref|NP_219486.1||hypothetical protein MGC9084 [Homo sapiens] sp|O95568.1|CA156_HUMAN UPF0558 protein C1orf156 (Arsenic transactivated protein 2) (AsTP2) emb|CAA23019.1| hypothetical protein [Homo sapiens] gb|AAH08679.1| C1orf156 protein [Homo sapiens] emb|CAG29326.1| MGC9084 [Homo sapiens] gb|AAU95377.1| arsenic-transactivated protein 2 [Homo sapiens] emb|CAI19361.1| chromosome 1 open reading frame 156 [Homo sapiens] gb|EAW90861.1| chromosome 1 open reading frame 156, isoform CRA_a [Homo sapiens] gb|EAW90862.1| chromosome 1 open reading frame 156, isoform CRA_a [Homo sapiens] dbj|BAG36632.1| unnamed protein product [Homo sapiens]; ref|XP_524959.1||PREDICTED: hypothetical protein MGC9084 isoform 3 [Pan troglodytes] ref|XP_001139157.1| PREDICTED: hypothetical protein MGC9084 isoform 1 [Pan troglodytes] ref|XP_001139315.1| PREDICTED: hypothetical protein MGC9084 isoform 2 [Pan troglodytes]; ref|NP_001020839.1||hypothetical protein LOC304928 [Rattus norvegicus] sp|Q4KM84.1|CA156_RAT UPF0558 protein C1orf156 homolog gb|AAH98702.1| Similar to 2810422O20Rik prot	-0,365895892	-0,47407021	-0,809629915	-0,898971932	-0,641850063	1,6	DOWN
366	C611810.1	No match	-0,494375929	-0,561834354	-0,721821452	-0,835776927	-0,641827903	1,6	DOWN
367	C610317.1	No match	-0,558599411	-0,60147784	-0,678622273	-0,733453271	-0,640050057	1,6	DOWN
368	C607250.1	No match	-0,496118432	-0,502237056	-0,777675808	-0,812976191	-0,639956432	1,6	DOWN
369	C603654.1	No match	-0,569878427	-0,484190584	-0,764699986	-0,707476372	-0,6386774	1,6	DOWN
370	C605489.1	gb|ACE06931.1||unknown [Schistosoma japonicum]; gb|AAP06215.1||hypothetical protein [Schistosoma japonicum];	-0,699429661	-0,670240853	-0,604693759	-0,470043375	-0,637467306	1,6	DOWN
371	C603825.1	gb|AAW24827.1||SJCHGC09339 protein [Schistosoma japonicum];	-0,600926878	-0,550854045	-0,672889306	-0,766586385	-0,636908092	1,6	DOWN
372	C606983.1	gb|AAD24794.1|AF120929_1|phosphoenolpyruvate carboxykinase [Schistosoma mansoni]; gb|AAW25103.1||SJCHGC06900 protein [Schistosoma japonicum]; emb|CAJ28913.1||phosphoenolpyruvate carboxykinase [Crassostrea gigas]; ref|NP_001021590.1||R11A5.4d [Caenorhabditis elegans] emb|CAF31483.1| C. elegans protein R11A5.4d, confirmed by transcript evidence [Caenorhabditis elegans];	-0,351091528	-0,440657555	-0,831364788	-0,893474242	-0,636011172	1,6	DOWN
373	C606441.1	gb|AAX25715.2||SJCHGC04764 protein [Schistosoma japonicum]; gb|EDW57502.1||GJ18122 [Drosophila virilis]; gb|EDW13455.1||GI18216 [Drosophila mojavensis]; gb|EDW03178.1||GH11094 [Drosophila grimshawi];	-0,895186169	-0,882310797	-0,388923877	-0,294027742	-0,635617337	1,6	DOWN
374	C605169.1	No match	-0,26359896	-0,425037562	-0,902431538	-0,845815725	-0,635426644	1,6	DOWN
375	C611231.1	No match	-0,669655067	-0,795493051	-0,44705371	-0,600867179	-0,635261123	1,6	DOWN
376	C609409.1	No match	-0,41337467	-0,480824414	-0,957901976	-0,787326881	-0,634075648	1,6	DOWN
377	C611476.1	gb|ABG78546.1||forkhead transcription factor [Schistosoma mansoni];	-0,632585707	-0,627285405	-0,665330406	-0,63523492	-0,633910314	1,6	DOWN

378	C612653.1	gb|AAW24976.1||SJCHGC08993 protein [Schistosoma japonicum]; ref|XP_972067.1||PREDICTED: similar to Protein C20orf11 (Two hybrid associated protein 1 with RanBPM) (Twa1) [Tribolium castaneum]; ref|XP_001600429.1||PREDICTED: similar to conserved hypothetical protein [Nasonia vitripennis]; ref|XP_392965.2||PREDICTED: similar to BWK-1 isoform 1 [Apis mellifera] ref|XP_623151.1| PREDICTED: similar to BWK-1 isoform 2 [Apis mellifera];	-0,551944485	-0,381848367	-0,950221559	-0,713386319	-0,632665402	1,6	DOWN
379	C601526.1	gb|AAW25382.1||SJCHGC03376 protein [Schistosoma japonicum]; ref|NP_001084550.1||hypothetical protein LOC414499 [Xenopus laevis] gb|AAH68951.1| MGC83218 protein [Xenopus laevis]; ref|NP_001007290.1||ribose 5-phosphate isomerase A (ribose 5-phosphate epimerase) [Danio rerio] gb|AAH85542.1| Ribose 5-phosphate isomerase A (ribose 5-phosphate epimerase) [Danio rerio]; gb|AAH67177.1||Rpia protein [Danio rerio];	-0,586304257	-0,550036021	-0,677975668	-0,732893745	-0,632139963	1,5	DOWN
380	C606453.1	gb|ABA40782.1||SJCHGC02235 protein [Schistosoma japonicum]; gb|AAX31017.1||SJCHGC09722 protein [Schistosoma japonicum];	-0,655068825	-0,597432542	-0,609560961	-0,654263601	-0,631912281	1,5	DOWN
381	C609670.1	No match	-0,830752613	-1,119959115	-0,432793736	-0,194690998	-0,631773175	1,5	DOWN
382	C606327.1	gb|ABN14897.1||mitochondrial ribosomal protein L15 [Taenia asiatica]; gb|AAX25668.2||SJCHGC06954 protein [Schistosoma japonicum]; gb|EDV52517.1||GG13310 [Drosophila erecta]; gb|EDX11241.1||GD12186 [Drosophila simulans];	-0,729354313	-0,768026379	-0,500860577	-0,532717218	-0,631035766	1,5	DOWN
383	C607294.1	No match	-0,700750699	-0,853038241	-0,560816071	-0,544355967	-0,630783385	1,5	DOWN
384	C611746.1	gb|AAX25413.2||SJCHGC05296 protein [Schistosoma japonicum]; gb|AAW27491.1||SJCHGC07173 protein [Schistosoma japonicum];	0,075829924	-0,27690037	-1,041861604	-0,984353649	-0,63062701	1,5	DOWN
385	C602194.1	No match	-0,486610323	-0,52438707	-0,79546358	-0,73513202	-0,629759545	1,5	DOWN
386	C604764.1	gb|AAW27023.1||SJCHGC06275 protein [Schistosoma japonicum]; gb|AAX30014.1||Ubc9 [Schistosoma mansoni] gb|AAX30015.1| Ubc9 [Schistosoma mansoni]; ref|NP_001040407.1||ubiquitin-conjugating enzyme E2 [Bombyx mori] gb|ABF51329.1| ubiquitin-conjugating enzyme E2 isoform 1 [Bombyx mori]; ref|XP_001640489.1||predicted protein [Nematostella vectensis] gb|EDO48426.1| predicted protein [Nematostella vectensis];	-0,353660369	-0,408217564	-0,849990515	-0,857817803	-0,62910404	1,5	DOWN
387	C602079.1	gb|AAX24641.2||SJCHGC04887 protein [Schistosoma japonicum];	-0,433515551	-0,56958375	-0,718074665	-0,687802087	-0,628692919	1,5	DOWN
388	C603705.1	gb|AAW26476.1||SJCHGC01111 protein [Schistosoma japonicum]; gb|AAX24756.2||SJCHGC07014 protein [Schistosoma japonicum]; ref|NP_694818.2||eukaryotic translation initiation factor 2C, 2 [Mus musculus] dbj|BAC15767.1| Piwi/Argonaute family protein meIF2C2 [Mus musculus]; sp|Q8CJG0|I2C2_MOUSE|Eukaryotic translation initiation factor 2C 2 (eIF2C 2) (eIF-2C 2) (Argonaute-2) (Slicer protein) (Piwi/argonaute family protein meIF2C2);	-0,429868748	-0,525439575	-0,729949127	-0,810828813	-0,627694351	1,5	DOWN
389	C606335.1	tpe|CAJ00252.1||TPA: gag-pol polyprotein [Schistosoma mansoni];	-0,584702823	-0,501043739	-0,668468778	-0,697702867	-0,626585801	1,5	DOWN
390	C609801.1	gb|AAW25936.1||SJCHGC05387 protein [Schistosoma japonicum];	-0,627373244	-0,624148854	-0,654393351	-0,603214794	-0,625761049	1,5	DOWN
391	C606909.1	No match	-0,293359501	-0,427218902	-0,822712418	-0,927728819	-0,62496566	1,5	DOWN

392	C610879.1	gb|AAX25661.2||SJCHGC04404 protein [Schistosoma japonicum];	-1,534908831	-0,96565788	-0,281636732	-0,267473006	-0,623647306	1,5	DOWN
393	C610625.1	gb|AAW25508.1||SJCHGC05689 protein [Schistosoma japonicum]; gb|AAH84616.1||LOC495283 protein [Xenopus laevis]; emb|CAF94336.1||unnamed protein product [Tetraodon nigroviridis]; ref|NP_001006926.1||aminolevulinate, delta-, synthase 2 [Xenopus tropicalis] gb|AAH76921.1| Aminolevulinate, delta-, synthase 2 [Xenopus tropicalis];	-0,608739319	-0,596021794	-0,667648924	-0,636878409	-0,622808864	1,5	DOWN
394	C606450.1	gb|AAC62254.1||lysophospholipase homolog [Schistosoma mansoni]; gb|ACE06918.1||unknown [Schistosoma japonicum]; gb|AAD52700.1|AF091539_1|lysophospholipase [Schistosoma japonicum]; ref|NP_001085785.1||MGC80756 protein [Xenopus laevis] gb|AAH73342.1| MGC80756 protein [Xenopus laevis];	-0,589648598	-0,560578205	-0,698077507	-0,655923056	-0,622785827	1,5	DOWN
395	C607608.1	ref|XP_001625612.1||predicted protein [Nematostella vectensis] gb|EDO33512.1| predicted protein [Nematostella vectensis]; ref|XP_623551.1||PREDICTED: similar to Recombination repair protein 1 (DNA-(apurinic or apyrimidinic site) lyase) [Apis mellifera]; ref|ZP_02431429.1||hypothetical protein CLOSCI_01649 [Clostridium scindens ATCC 35704] gb|EDS07306.1| hypothetical protein CLOSCI_01649 [Clostridium scindens ATCC 35704]; ref|ZP_02272247.1||Exodeoxyribonuclease III [Fusobacterium nucleatum subsp. polymorphum ATCC 10953] gb|EDK87855.1| exodeoxyribonuclease III [Fusobacterium nucleatum subsp. polymorphum ATCC 10953];	-0,550222463	-0,403879208	-0,779403384	-0,694505239	-0,622363851	1,5	DOWN
396	C602071.1	ref|NP_998628.1||eukaryotic translation initiation factor 3, subunit C [Danio rerio] gb|AAH57465.1| Eukaryotic translation initiation factor 3, subunit C [Danio rerio]; ref|XP_623580.2||PREDICTED: similar to eIF3-S8 CG4954-PA [Apis mellifera]; ref|XP_001807880.1||PREDICTED: similar to translation initiation factor 3, subunit S8, putative [Tribolium castaneum]; emb|CAG07597.1||unnamed protein product [Tetraodon nigroviridis];	-0,71026866	-0,687807106	-0,553143181	-0,413168504	-0,620475144	1,5	DOWN
397	C601311.1	No match	-0,464074408	-0,483171632	-0,751961724	-0,778535408	-0,617566678	1,5	DOWN
398	C610638.1	No match	-0,677673307	-0,598892265	-0,630580394	-0,520729682	-0,61473633	1,5	DOWN
399	C611201.1	No match	-0,947065563	-1,03817599	-0,277276058	-0,281318559	-0,614192061	1,5	DOWN
400	C612132.1	No match	-0,30489902	-1,072573752	-0,52015886	-0,707872026	-0,614015443	1,5	DOWN
401	C703445.1	gb|AAO17782.1||wnt5-like protein [Girardia tigrina]; ref|NP_001006767.1||wingless-type MMTV integration site family, member 5B [Xenopus tropicalis] gb|AAH75560.1| Wingless-type MMTV integration site family, member 5B [Xenopus tropicalis] emb|CAJ83472.1| wingless-type MMTV integration site family, member 5B [Xenopus tropicalis]; ref|NP_001080150.1||C. elegans WNT family member precursor (40.4 kD) (wnt-2) [Xenopus laevis] gb|AAH56128.1| Wnt-2-prov protein [Xenopus laevis]; sp|P33945|WNT5C_XENLA|Protein Wnt-5c precursor (XWnt-5c) emb|CAA51916.1| Xwnt-5c [Xenopus laevis];	-0,556005618	-0,363844036	-0,851619916	-0,671087658	-0,613546638	1,5	DOWN

402	C601194.1	dbj|BAF83282.1||unnamed protein product [Homo sapiens]; ref|XP_001116610.1||PREDICTED: similar to alpha isoform of regulatory subunit A, protein phosphatase 2 isoform 10 [Macaca mulatta] ref|XP_001174541.1| PREDICTED: alpha isoform of regulatory subunit A, protein phosphatase 2 isoform 9 [Pan troglodytes]; emb|CAG29336.1||PPP2R1A [Homo sapiens]; ref|NP_055040.2||alpha isoform of regulatory subunit A, protein phosphatase 2 [Homo sapiens] ref|XP_001116592.1| PREDICTED: similar to alpha isoform of regulatory subunit A, protein phosphatase 2 isoform 8 [Macaca mulatta] ref|XP_001116604.1| PREDICTED: similar to alpha isoform of regulatory subunit A, protein phosphatase 2 isoform 9 [Macaca mulatta] ref|XP_001116617.1| PREDICTED: similar to alpha isoform of regulatory subunit A, protein phosphatase 2 isoform 11 [Macaca mulatta] ref|XP_001174546.1| PREDICTED: alpha isoform of regulatory subunit A, protein phosphatase 2 isoform 10 [Pan troglodytes] sp|P30153|2AAA_HUMAN Serine/threonine-protein phosphatase 2A 65 kDa regulatory subunit A alpha isoform (PP2A, subunit A, PR65-alpha isoform) (PP2A, subunit A, R1-alpha	-0,723542192	-0,630323774	-0,51318119	-0,59223519	-0,611279482	1,5	DOWN
403	C601555.1	gb|AAU34080.1||glutathione peroxidase-2 [Schistosoma mansoni]; gb|ABK58680.1||PHGPx isoform 2 [Clonorchis sinensis]; gb|ABE68811.1||phospholipid hydroperoxide glutathione peroxidase isoform 1 [Paragonimus westermani]; gb|ABE68812.1||phospholipid hydroperoxide glutathione peroxidase isoform 2 [Paragonimus westermani];	-0,453957832	-0,545585477	-0,675683465	-0,68571532	-0,610634471	1,5	DOWN
404	C602425.1	No match	-0,457135175	-0,011191309	-0,777271652	-0,763460702	-0,610297939	1,5	DOWN
405	C603422.1	gb|AAW26144.1||SJCHGC02223 protein [Schistosoma japonicum]; ref|NP_955885.1||eukaryotic translation initiation factor 5 [Danio rerio] gb|AAH49502.1| Eukaryotic translation initiation factor 5 [Danio rerio] gb|AAH66614.1| Eukaryotic translation initiation factor 5 [Danio rerio] gb|AAS92648.1| eukaryotic translation initiation factor 5 [Danio rerio] emb|CAI21349.1| novel protein (zgc:77026) [Danio rerio]; ref|XP_001507766.1||PREDICTED: similar to Eukaryotic translation initiation factor 5 [Ornithorhynchus anatinus]; ref|XP_001366943.1||PREDICTED: hypothetical protein [Monodelphis domestica];	-0,446225568	-0,361967535	-0,799693893	-0,77186155	-0,609043559	1,5	DOWN
406	C603036.1	ref|NP_001090412.1||hypothetical protein LOC779324 [Xenopus laevis] gb|AAI23367.1| MGC154882 protein [Xenopus laevis]; ref|XP_786594.2||PREDICTED: hypothetical protein [Strongylocentrotus purpuratus] ref|XP_001176705.1| PREDICTED: hypothetical protein [Strongylocentrotus purpuratus]; ref|XP_001364011.1||PREDICTED: similar to C14orf126 protein [Monodelphis domestica]; ref|XP_001512645.1||PREDICTED: similar to C14orf126 protein [Ornithorhynchus anatinus];	-0,484654255	-0,443022517	-0,729842536	-0,754922363	-0,607248396	1,5	DOWN
407	C602131.1	gb|AAW24903.1||SJCHGC05946 protein [Schistosoma japonicum]; gb|AAK29203.1|AF225905_1|ribosomal protein S15a [Taenia solium] gb|AAP35026.1| ribosomal S15a protein [Taenia solium] gb|AAP35027.1| ribosomal S15a protein [Taenia saginata]; gb|ACD65146.1||putative 40S ribosomal protein RPS15A [Phoronis muelleri]; gb|ABR27827.1||40S ribosomal protein S15/S22 [Triatoma infestans];	-0,395914531	-0,450841523	-0,803275038	-0,762606286	-0,606723905	1,5	DOWN

408	C705316.1	dbj|BAG54197.1||unnamed protein product [Homo sapiens]; sp|Q6NXD8.2|RA61L_DANRE|RAB6IP1-like protein; ref|XP_001477177.1||PREDICTED: similar to Rab6 interacting protein 1 [Mus musculus]; gb|EDL16964.1||Rab6 interacting protein 1, isoform CRA_b [Mus musculus] gb|EDL16965.1| Rab6 interacting protein 1, isoform CRA_b [Mus musculus];	-0,539797102	-0,576570663	-0,692698852	-0,634687162	-0,605628913	1,5	DOWN
409	C603342.1	gb|AAX25143.2||SJCHGC07760 protein [Schistosoma japonicum];	-0,737839317	-0,595878173	-0,61351056	-0,436939804	-0,604694367	1,5	DOWN
410	C610415.1	gb|AAW25037.1||SJCHGC03665 protein [Schistosoma japonicum]; ref|NP_001072776.1||cyclin-dependent kinase 5 [Xenopus tropicalis] gb|AAI18781.1| Cyclin-dependent kinase 5 [Xenopus tropicalis] gb|AAI35927.1| Cyclin-dependent kinase 5 [Xenopus tropicalis]; ref|NP_001084086.1||neuronal cyclin-dependent kinase 5 [Xenopus laevis] sp|P51166|CDK5_XENLA Cell division protein kinase 5 (Cyclin-dependent kinase 5) (Neuronal cyclin-dependent kinase 5) gb|AAB37091.1| neuronal cyclin-dependent kinase 5 gb|AAH72894.1| Cdk5 protein [Xenopus laevis]; gb|AAP36712.1||Homo sapiens cyclin-dependent kinase 5 [synthetic construct] gb|AAQ02523.1| cyclin-dependent kinase 5 [synthetic construct] gb|AAV38941.1| cyclin-dependent kinase 5 [synthetic construct] gb|AAX36868.1| cyclin-dependent kinase 5 [synthetic construct] gb|AAX43084.1| cyclin-dependent kinase 5 [synthetic construct] gb|AAX43934.1| cyclin-dependent kinase 5 [synthetic construct] gb|AAX43935.1| cyclin-dependent kinase 5 [synthetic construct];	-0,63842027	-0,639712526	-0,569342227	-0,413694723	-0,603881249	1,5	DOWN
411	C600552.1	No match	-0,443640744	-0,493804465	-0,713417271	-0,855040906	-0,603610868	1,5	DOWN
412	C610467.1	gb|AAW27291.1||SJCHGC06584 protein [Schistosoma japonicum]; ref|NP_001086339.1||MGC81898 protein [Xenopus laevis] gb|AAH75131.1| MGC81898 protein [Xenopus laevis]; gb|AAN71554.1||RH27395p [Drosophila melanogaster]; ref|NP_001089775.1||hypothetical protein LOC734839 [Xenopus laevis] gb|AAI06543.1| MGC131302 protein [Xenopus laevis];	-0,605379397	-0,597833669	-0,641487896	-0,593046429	-0,601606533	1,5	DOWN
413	C600632.1	gb|AAX26725.2||SJCHGC04721 protein [Schistosoma japonicum]; ref|XP_001950539.1||PREDICTED: similar to AGAP004743-PA [Acyrthosiphon pisum]; ref|XP_791284.1||PREDICTED: similar to transmembrane protein [Strongylocentrotus purpuratus] ref|XP_001179656.1| PREDICTED: similar to transmembrane protein [Strongylocentrotus purpuratus]; ref|XP_971914.1||PREDICTED: similar to predicted protein [Tribolium castaneum];	-0,453723325	-0,826558102	-0,666803951	-0,535401289	-0,60110262	1,5	DOWN
414	C600861.1	gb|ABG21818.1||B-cell receptor-associated protein-like protein [Schistosoma mansoni]; gb|AAW27231.1||SJCHGC06848 protein [Schistosoma japonicum]; ref|XP_001656331.1||bcr-associated protein, bap [Aedes aegypti] gb|EAT45594.1| bcr-associated protein, bap [Aedes aegypti]; ref|XP_001656330.1||bcr-associated protein, bap [Aedes aegypti] gb|EAT45593.1| bcr-associated protein, bap [Aedes aegypti];	-0,596265219	-0,427561122	-0,617861767	-0,605422804	-0,600844012	1,5	DOWN

415	C607157.1	ref|XP_001923230.1||PREDICTED: alpha thalassemia/mental retardation syndrome X-linked, like [Danio rerio]; gb|AAH57486.1||Atrxl protein [Danio rerio]; gb|AAH47849.1||Atrxl protein [Danio rerio]; ref|XP_001128623.2||PREDICTED: similar to putative DNA dependent ATPase and helicase [Homo sapiens];	-0,571726607	-0,614356122	-0,586139497	-0,686655948	-0,60024781	1,5	DOWN
416	C609195.1	No match	-0,445934252	-0,478288512	-0,788971516	-0,721799949	-0,600044231	1,5	DOWN
417	C602569.1	gb|AAW26420.1||SJCHGC06602 protein [Schistosoma japonicum]; ref|NP_001013487.1||signal peptidase complex subunit 2 homolog [Danio rerio] sp|Q5BJI9|SPCS2_BRARE Probable signal peptidase complex subunit 2 (Microsomal signal peptidase 25 kDa subunit) (SPase 25 kDa subunit) gb|AAH91463.1| Signal peptidase complex subunit 2 homolog (S. cerevisiae) [Danio rerio]; gb|AAO27767.1||signal peptidase 25 kDA subunit [Gasterosteus aculeatus]; emb|CAG02153.1||unnamed protein product [Tetraodon nigroviridis];	-0,208752419	-0,229425026	-0,986949005	-0,970317314	-0,59987117	1,5	DOWN
418	C610344.1	gb|AAW27211.1||SJCHGC09295 protein [Schistosoma japonicum]; gb|AAX25500.2||SJCHGC05365 protein [Schistosoma japonicum];	-0,553865619	-0,554316003	-0,752827672	-0,645273882	-0,599794943	1,5	DOWN
419	C604353.1	ref|XP_001942806.1||PREDICTED: similar to CG10671 CG10671-PA [Acyrthosiphon pisum]; gb|EDW93488.1||GE20570 [Drosophila yakuba]; gb|EDW50561.1||GM13929 [Drosophila sechellia]; gb|AAL13842.1||LD30661p [Drosophila melanogaster];	-0,392987957	-0,052494995	-0,93815732	-0,806277741	-0,599632849	1,5	DOWN
420	C612571.1	gb|AAW25086.1||SJCHGC09282 protein [Schistosoma japonicum]; ref|XP_001632964.1||predicted protein [Nematostella vectensis] gb|EDO40901.1| predicted protein [Nematostella vectensis]; ref|NP_998955.1||histidine triad protein member 5 [Sus scrofa] sp|Q8MIZ3|DCPS_PIG Scavenger mRNA-decapping enzyme DcpS (DCS-1) (Hint-related 7meGMP-directed hydrolase) (Histidine triad protein member 5) (HINT-5) gb|AAK91766.1| histidine triad protein member 5 [Sus scrofa]; sp|Q8MJJ7|DCPS_BOVIN|Scavenger mRNA-decapping enzyme DcpS (DCS-1) (Hint-related 7meGMP-directed hydrolase) (Histidine triad protein member 5) (HINT-5) gb|AAM90585.1| HINT-5 [Bos taurus];	-0,392692867	-0,592429664	-0,605681233	-0,830351671	-0,599055449	1,5	DOWN
421	C610016.1	gb|AAW24791.1||SJCHGC06107 protein [Schistosoma japonicum]; gb|EDV98017.1||GH14373 [Drosophila grimshawi]; ref|XP_624693.1||PREDICTED: similar to Aut1 CG6877-PA [Apis mellifera]; gb|EDW19783.1||GI11317 [Drosophila mojavensis];	-0,487360129	-0,553694337	-0,707628338	-0,643837477	-0,598765907	1,5	DOWN
422	C606333.1	gb|AAW27044.1||SJCHGC05003 protein [Schistosoma japonicum]; gb|AAP06320.1||hypothetical protein [Schistosoma japonicum]; gb|EDW76652.1||GK14546 [Drosophila willistoni]; ref|NP_609619.1||CG16812 CG16812-PA [Drosophila melanogaster] gb|AAF53264.1| CG16812-PA [Drosophila melanogaster] gb|AAL13891.1| LD36945p [Drosophila melanogaster];	-0,419474551	-0,539298687	-0,65745261	-0,840725244	-0,598375649	1,5	DOWN
423	C605292.1	No match	-0,481950529	-0,395408405	-0,712955357	-0,779706149	-0,597452943	1,5	DOWN
424	C611615.1	No match	-0,452713782	-0,507253196	-0,729372472	-0,687121829	-0,597187513	1,5	DOWN

425	C600144.1	gb|AAX27647.2||SJCHGC05167 protein [Schistosoma japonicum]; gb|AAP06067.1||similar to NM_021205 CDC42-like GTPase; novel Ras family protein; Wrch-1; Ryu GTPase in Homo sapiens [Schistosoma japonicum]; gb|AAX20136.1||ras-like protein Rhoua [Danio rerio]; ref|NP_001007444.1||ras homolog gene family, member Ua [Danio rerio] gb|AAH85398.1| Ras homolog gene family, member Ua [Danio rerio] emb|CAK04404.1| ras homolog gene family, member Ua [Danio rerio] gb|AAI65147.1| Rhoua protein [synthetic construct];	-0,519503109	-0,453792331	-0,67240096	-0,748779696	-0,595952035	1,5	DOWN
426	C604407.1	gb|AAW26910.1||SJCHGC06873 protein [Schistosoma japonicum]; ref|NP_001108317.1||hypothetical protein LOC558272 [Danio rerio] emb|CAM16012.1| novel protein similar to vertebrate hexosaminidase A (alpha polypeptide) (HEXA) [Danio rerio]; gb|AAB30707.2||beta-hexosaminidase beta subunit [Felis catus]; ref|NP_001009333.1||hexosaminidase B [Felis catus] gb|AAB67612.1| beta-N-acetylhexosaminidase beta subunit [Felis catus];	-0,486076452	-0,539022213	-0,652028404	-0,731317962	-0,595525309	1,5	DOWN
427	C611867.1	gb|AAW26628.1||SJCHGC05347 protein [Schistosoma japonicum];	-0,682912309	-0,740449846	-0,507602545	-0,35248306	-0,595257427	1,5	DOWN
428	C610657.1	No match	-0,807871891	-0,7050261	-0,480252517	-0,468191458	-0,592639309	1,5	DOWN
429	C604912.1	gb|ABK60662.1||conserved hypothetical protein [Clostridium novyi NT] gb|ABK60736.1| conserved hypothetical protein [Clostridium novyi NT] gb|ABK60862.1| conserved hypothetical protein [Clostridium novyi NT] gb|ABK61024.1| conserved hypothetical protein [Clostridium novyi NT] gb|ABK61536.1| conserved hypothetical protein [Clostridium novyi NT] gb|ABK62404.1| conserved hypothetical protein [Clostridium novyi NT] gb|ABK62644.1| conserved hypothetical protein [Clostridium novyi NT] gb|ABK62651.1| conserved hypothetical protein [Clostridium novyi NT]; ref|ZP_02863215.1||conserved hypothetical protein [Clostridium botulinum C str. Eklund] ref|ZP_02863226.1| conserved hypothetical protein [Clostridium botulinum C str. Eklund] ref|ZP_02863230.1| conserved hypothetical protein [Clostridium botulinum C str. Eklund] gb|ABK62310.1| conserved hypothetical protein [Clostridium novyi NT] gb|ABK62642.1| conserved hypothetical protein [Clostridium novyi NT] gb|EDS76142.1| conserved hypothetical protein [Clostridium botulinum C str. Eklund] gb|EDS76170.1| conserved hypothetical protein [Clostridium botulinum C str. Eklund] gb|EDS76282.1| conse	-0,458535984	-0,231097205	-0,726422756	-0,790090652	-0,59247937	1,5	DOWN
430	C610488.1	No match	-0,503845811	-0,403741162	-0,724443281	-0,67525638	-0,589551096	1,5	DOWN
431	C608151.1	No match	-0,521168191	-0,812821882	-0,533040938	-0,645532565	-0,589286752	1,5	DOWN
432	C603564.1	No match	-0,397623895	-0,514751961	-0,662104166	-0,675515167	-0,588428064	1,5	DOWN
433	C601781.1	No match	-0,507521812	-0,477425344	-0,6945878	-0,668612803	-0,588067308	1,5	DOWN
434	C608034.1	gb|AAB86568.1||unknown [Schistosoma mansoni]; gb|AAB86567.1||unknown [Schistosoma mansoni]; gb|AAW26969.1||SJCHGC04169 protein [Schistosoma japonicum];	-0,457090348	0,016264198	-1,155755247	-0,718781832	-0,58793609	1,5	DOWN
435	C610146.1	tpg|DAA04497.1||TPA_exp: pol polyprotein [Schistosoma mansoni];	-0,602477053	-0,575168229	-0,600231111	-0,3697049	-0,58769967	1,5	DOWN
436	C611006.1	No match	-0,743273213	-0,844804471	-0,42967295	-0,383605637	-0,586473082	1,5	DOWN

437	C601609.1	gb|AAW25659.1||SJCHGC01319 protein [Schistosoma japonicum]; gb|AAO86771.1||casein kinase II beta subunit [Schistosoma japonicum]; gb|AAP06151.1||similar to NM_131187 casein kinase 2 beta in Danio rerio [Schistosoma japonicum]; gb|AAP06476.1||similar to NM_009975 Casein kinase II beta subunit in Homo sapiens [Schistosoma japonicum];	-0,513865068	-0,205575041	-1,043355309	-0,657722888	-0,585793978	1,5	DOWN
438	C605137.1	sp|Q5BQX9.1|SJ766_SCHJA|Uncharacterized protein SJCHGC09766 precursor gb|AAX31057.1| SJCHGC09766 protein [Schistosoma japonicum]; sp|Q5BQU6.1|SJ803_SCHJA|Uncharacterized protein SJCHGC09803 precursor gb|AAX31090.1| SJCHGC09803 protein [Schistosoma japonicum]; sp|Q5BQU9.1|SJ800_SCHJA|Uncharacterized protein SJCHGC09800 precursor gb|AAX31087.1| SJCHGC09800 protein [Schistosoma japonicum];	-0,136049634	-0,235286221	-0,935911741	-1,016223612	-0,585598981	1,5	DOWN
439	C609659.1	gb|AAW24768.1||SJCHGC00713 protein [Schistosoma japonicum];	-0,415926235	-0,390740434	-0,755111263	-0,795693536	-0,585518749	1,5	DOWN
440	C603157.1	gb|AAX27791.2||SJCHGC02238 protein [Schistosoma japonicum]; ref|NP_997856.1||syndecan binding protein [Danio rerio] gb|AAH44454.1| Syndecan binding protein (syntenin) [Danio rerio] gb|AAQ97846.1| syndecan binding protein [Danio rerio] gb|AAH65851.1| Syndecan binding protein (syntenin) [Danio rerio] gb|AAI64064.1| Sdcbp protein [synthetic construct]; ref|NP_001079704.1||similar to syndecan binding protein (syntenin) [Xenopus laevis] gb|AAH48018.1| MGC52622 protein [Xenopus laevis]; ref|XP_001843453.1||syntenin-1 [Culex pipiens quinquefasciatus] gb|EDS32612.1| syntenin-1 [Culex quinquefasciatus];	-0,43412785	-0,492545137	-0,6897555	-0,678194339	-0,585369738	1,5	DOWN
441	C610329.1	No match	-0,235246876	-0,262418863	-0,907713129	-0,940831568	-0,585065996	1,5	DOWN
442	C609515.1	No match	-0,536716329	-0,688588191	-0,596599712	-0,573272664	-0,584936188	1,5	DOWN
443	C603215.1	No match	-0,421296662	-0,445475866	-0,773149084	-0,72177162	-0,583623743	1,5	DOWN
444	C711508.1	ref|XP_001869981.1||dihydrolipoamide dehydrogenase [Culex pipiens quinquefasciatus] gb|EDS31040.1| dihydrolipoamide dehydrogenase [Culex quinquefasciatus]; ref|NP_502753.2||LLC1.3 [Caenorhabditis elegans] emb|CAB05249.2| C. elegans protein LLC1.3, confirmed by transcript evidence [Caenorhabditis elegans]; ref|XP_320877.4||AGAP011629-PA [Anopheles gambiae str. PEST] gb|EAA00422.4| AGAP011629-PA [Anopheles gambiae str. PEST]; ref|XP_782447.2||PREDICTED: similar to Dihydrolipoyl dehydrogenase, mitochondrial precursor (Dihydrolipoamide dehydrogenase) [Strongylocentrotus purpuratus] ref|XP_001194682.1| PREDICTED: similar to Dihydrolipoyl dehydrogenase, mitochondrial precursor (Dihydrolipoamide dehydrogenase) [Strongylocentrotus purpuratus];	-0,65035121	-0,828077704	-0,469232079	-0,515010602	-0,582680906	1,5	DOWN
445	C602785.1	No match	-0,261847988	-0,491553059	-0,733865616	-0,672768141	-0,5821606	1,5	DOWN
446	C612007.1	gb|ACE06877.1||unknown [Schistosoma japonicum]; gb|AAP06287.1||similar to GenBank Accession Number AE003423 CG3073 gene product in Drosophila melanogaster [Schistosoma japonicum];	-0,481324537	-0,465134382	-0,680861196	-0,899840936	-0,581092867	1,5	DOWN
447	C601690.1	No match	-0,704471502	-1,097268164	-0,413682996	-0,455428108	-0,579949805	1,5	DOWN

448	C609870.1	gb|AAX27166.2||SJCHGC05459 protein [Schistosoma japonicum]; gb|AAI23082.1||LOC779589 protein [Xenopus tropicalis]; ref|XP_001376019.1||PREDICTED: hypothetical protein [Monodelphis domestica]; ref|NP_001006148.1||bromodomain containing 8 [Gallus gallus] emb|CAG32596.1| hypothetical protein [Gallus gallus];	-0,438708471	-0,450391997	-0,709017591	-0,711211245	-0,579704794	1,5	DOWN
449	C608304.1	gb|EDV42819.1||GF16839 [Drosophila ananassae]; gb|EDW25876.1||GL14258 [Drosophila persimilis]; gb|EDW66433.1||GJ15556 [Drosophila virilis]; gb|EDW06198.1||GI16044 [Drosophila mojavensis];	-0,535481578	-0,398993516	-0,790334289	-0,622273721	-0,57887765	1,5	DOWN
450	C607711.1	gb|AAW26347.1||SJCHGC02297 protein [Schistosoma japonicum]; emb|CAF90783.1||unnamed protein product [Tetraodon nigroviridis]; gb|EDL30780.1||Yip1 domain family, member 1, isoform CRA_d [Mus musculus]; emb|CAM23043.1||Yip1 domain family, member 1 [Mus musculus];	-0,925319792	-0,964556831	-0,180332261	-0,23141968	-0,578369736	1,5	DOWN
451	C603814.1	emb|CAD21542.1||hypothetical protein [Taenia solium];	-0,40843961	-0,247203522	-0,74369658	-0,743071916	-0,575755763	1,5	DOWN
452	C611329.1	gb|AAP05966.1||hypothetical protein [Schistosoma japonicum]; gb|AAW27241.1||SJCHGC02711 protein [Schistosoma japonicum];	-0,677547302	-0,628212448	-0,519671804	-0,447098753	-0,573942126	1,5	DOWN
453	C605963.1	No match	-0,660033729	-0,559647905	-0,588227861	-0,414009757	-0,573937883	1,5	DOWN
454	C608763.1	ref|XP_001488649.2||PREDICTED: similar to Splicing factor 3b, subunit 4 [Equus caballus]; ref|XP_001926524.1||PREDICTED: similar to Splicing factor 3b, subunit 4 [Sus scrofa]; ref|XP_001365466.1||PREDICTED: similar to Splicing factor 3b, subunit 4 [Monodelphis domestica]; ref|XP_423721.2||PREDICTED: similar to Splicing factor 3b, subunit 4 [Gallus gallus];	-0,224782498	-0,285323824	-0,862235636	-1,090589893	-0,57377973	1,5	DOWN
455	C600467.1	gb|AAX28044.2||SJCHGC08177 protein [Schistosoma japonicum]; gb|AAX25108.2||SJCHGC02950 protein [Schistosoma japonicum];	-0,624784085	-0,575466451	-0,570056454	-0,518032065	-0,572761453	1,5	DOWN
456	C600512.1	gb|AAW25846.1||SJCHGC06777 protein [Schistosoma japonicum]; gb|AAW27680.1||SJCHGC02820 protein [Schistosoma japonicum];	-0,648024099	-0,629196787	-0,515192196	-0,486003702	-0,572194492	1,5	DOWN
457	C608846.1	No match	-0,37612044	-0,387014357	-0,75578106	-0,826621685	-0,571397709	1,5	DOWN
458	C604746.1	prf||2019440B|Sm65 antigen; gb|AAX26501.2||SJCHGC07856 protein [Schistosoma japonicum];	-0,756843404	-0,647937596	-0,400594744	-0,493458047	-0,570697822	1,5	DOWN
459	C602619.1	gb|AAW24565.1||SJCHGC03729 protein [Schistosoma japonicum];	-0,358444384	-0,506075389	-0,631962644	-0,842245983	-0,569019017	1,5	DOWN
460	C610620.1	No match	-0,698217761	-0,670807079	-0,465592953	-0,346095888	-0,568200016	1,5	DOWN
461	C600271.1	gb|AAX30374.1||SJCHGC03136 protein [Schistosoma japonicum]; gb|AAX25849.2||SJCHGC03094 protein [Schistosoma japonicum]; gb|AAW27225.1||SJCHGC04667 protein [Schistosoma japonicum];	-0,584208771	-0,436358994	-0,592678	-0,551277928	-0,56774335	1,5	DOWN

462	C606402.1	ref|NP_705949.1||spinster homolog 1 [Danio rerio] gb|AAL69987.1|AF465772_1 not really started [Danio rerio]; sp|Q7ZU13.1|SPNS1_DANRE|Protein spinster homolog 1 (Spinster-like protein) (Protein not really started) gb|AAH48024.1| Spinster homolog 1 (Drosophila) [Danio rerio] gb|AAI65625.1| Spns1 protein [synthetic construct]; ref|XP_972584.1||PREDICTED: similar to spinster CG8428-PC [Tribolium castaneum]; ref|XP_001204134.1||PREDICTED: similar to RIKEN cDNA 2210013K02 gene, partial [Strongylocentrotus purpuratus] ref|XP_787421.2| PREDICTED: similar to RIKEN cDNA 2210013K02 gene, partial [Strongylocentrotus purpuratus];	-0,772560883	-0,623084733	-0,49559909	-0,509846917	-0,566465825	1,5	DOWN
463	C609740.1	gb|AAX30243.3||SJCHGC02237 protein [Schistosoma japonicum]; gb|AAX28303.2||SJCHGC01433 protein [Schistosoma japonicum]; ref|XP_971825.1||PREDICTED: similar to signal recognition particle receptor beta subunit [Tribolium castaneum]; ref|XP_001641944.1||predicted protein [Nematostella vectensis] gb|EDO49881.1| predicted protein [Nematostella vectensis];	-0,673945442	-0,541497434	-0,548120598	-0,584543969	-0,566332284	1,5	DOWN
464	C604860.1	gb|AAW27060.1||SJCHGC04744 protein [Schistosoma japonicum];	-0,278580728	-0,370180473	-0,761679509	-0,763354563	-0,565929991	1,5	DOWN
465	C605752.1	No match	-0,487847117	-0,395952809	-0,64335145	-0,732923121	-0,565599284	1,5	DOWN
466	C609775.1	gb|AAX27411.2||SJCHGC08952 protein [Schistosoma japonicum]; ref|XP_395705.3||PREDICTED: similar to CG18497-PA, isoform A, partial [Apis mellifera]; ref|XP_001606953.1||PREDICTED: similar to predicted protein [Nasonia vitripennis]; ref|XP_001689173.1||AGAP009990-PA [Anopheles gambiae str. PEST] gb|EDO63446.1| AGAP009990-PA [Anopheles gambiae str. PEST];	-0,757047431	-0,551697818	-0,43681148	-0,577878951	-0,564788385	1,5	DOWN
467	C609274.1	gb|AAX26685.2||SJCHGC04347 protein [Schistosoma japonicum];	-0,416429591	-0,502755187	-0,625438157	-0,770881755	-0,564096672	1,5	DOWN
468	C602410.1	gb|AAW26110.1||SJCHGC06773 protein [Schistosoma japonicum]; gb|AAX25852.2||SJCHGC06264 protein [Schistosoma japonicum]; gb|AAP06361.1||similar to GenBank Accession Number AK004968 putative ribophorin II in Mus musculus [Schistosoma japonicum]; ref|NP_001079661.1||similar to ribophorin II [Xenopus laevis] gb|AAH46727.1| MGC53764 protein [Xenopus laevis];	-0,174429685	-0,352305128	-0,808213396	-0,773134111	-0,56271962	1,5	DOWN
469	C716135.1	ref|NP_001098258.1||caspase-8 [Oryzias latipes] gb|AAS91704.1| caspase-8 [Oryzias latipes]; ref|XP_001184391.1||PREDICTED: similar to caspase-3 [Strongylocentrotus purpuratus]; ref|XP_792621.2||PREDICTED: similar to caspase-3, partial [Strongylocentrotus purpuratus] ref|XP_001184344.1| PREDICTED: similar to caspase-3, partial [Strongylocentrotus purpuratus]; ref|NP_001026949.1||caspase-8 [Sus scrofa] gb|AAS91710.1| caspase-8 [Sus scrofa];	-0,564230198	-1,124543241	-0,557733057	-0,455824212	-0,560981628	1,5	DOWN

470	C604638.1	ref|XP_789857.2||PREDICTED: hypothetical protein [Strongylocentrotus purpuratus] ref|XP_001176595.1| PREDICTED: hypothetical protein [Strongylocentrotus purpuratus]; ref|NP_001093679.1||hypothetical protein LOC100101683 [Xenopus tropicalis] gb|AAI35670.1| C19orf10 protein [Xenopus tropicalis]; emb|CAG08012.1||unnamed protein product [Tetraodon nigroviridis]; ref|XP_854682.1||PREDICTED: similar to chromosome 19 open reading frame 10 [Canis familiaris];	-0,492428549	-0,500130995	-0,620981498	-0,692742969	-0,560556247	1,5	DOWN
471	C605194.1	No match	-0,329761164	-0,36840612	-0,752355241	-0,755486133	-0,560380681	1,5	DOWN
472	C602592.1	gb|AAW27615.1||SJCHGC02437 protein [Schistosoma japonicum];	-0,339392526	-0,362893851	-0,757859029	-0,761551717	-0,56037644	1,5	DOWN
473	C606545.1	ref|ZP_02844269.1||hypothetical protein Tmz1tDRAFT_3429 [Thauera sp. MZ1T] gb|EDS55905.1| hypothetical protein Tmz1tDRAFT_3429 [Thauera sp. MZ1T]; ref|ZP_02008652.1||hypothetical protein Rpic12DDRAFT_1928 [Ralstonia pickettii 12D] gb|EDN40584.1| hypothetical protein Rpic12DDRAFT_1928 [Ralstonia pickettii 12D]; ref|ZP_02863229.1||conserved hypothetical protein [Clostridium botulinum C str. Eklund] gb|EDS76152.1| conserved hypothetical protein [Clostridium botulinum C str. Eklund]; ref|ZP_02863215.1||conserved hypothetical protein [Clostridium botulinum C str. Eklund] ref|ZP_02863226.1| conserved hypothetical protein [Clostridium botulinum C str. Eklund] ref|ZP_02863230.1| conserved hypothetical protein [Clostridium botulinum C str. Eklund] gb|ABK62310.1| conserved hypothetical protein [Clostridium novyi NT] gb|ABK62642.1| conserved hypothetical protein [Clostridium novyi NT] gb|EDS76142.1| conserved hypothetical protein [Clostridium botulinum C str. Eklund] gb|EDS76170.1| conserved hypothetical protein [Clostridium botulinum C str. Eklund] gb|EDS76282.1| conserved	-0,767242205	-0,483285576	-0,636508464	-0,466164158	-0,55989702	1,5	DOWN
474	C601120.1	gb|AAW26691.1||SJCHGC07316 protein [Schistosoma japonicum]; ref|NP_001002868.1||dpy-19-like 1, like [Danio rerio] gb|AAT68046.1| KIAA0877-like [Danio rerio] gb|AAI63534.1| Dpy-19-like 1, like (H. sapiens) [Danio rerio] gb|AAI63516.1| Dpy-19-like 1, like (H. sapiens) [Danio rerio]; ref|XP_001900769.1||Protein dpy-19 [Brugia malayi] gb|EDP30524.1| Protein dpy-19, putative [Brugia malayi]; ref|XP_785579.1||PREDICTED: similar to KIAA0877-like [Strongylocentrotus purpuratus] ref|XP_001187578.1| PREDICTED: similar to KIAA0877-like [Strongylocentrotus purpuratus];	-0,501837456	-0,525096351	-0,594461905	-0,754229725	-0,559779128	1,5	DOWN
475	C600025.1	gb|AAX30374.1||SJCHGC03136 protein [Schistosoma japonicum];	-0,532014102	-0,630108441	-0,586840209	-0,136408955	-0,559427156	1,5	DOWN
476	C601665.1	pdb|1VYF|A|Chain A, Schistosoma Mansoni Fatty Acid Binding Protein In Complex With Oleic Acid pdb|1VYG|A Chain A, Schistosoma Mansoni Fatty Acid Binding Protein In Complex With Arachidonic Acid; sp|P29498|FABP_SCHMA|14 kDa fatty acid-binding protein (Sm14) gb|AAA63516.1| fatty acid binding protein gb|AAL15461.1| fatty acid-binding protein Sm14 [Schistosoma mansoni]; gb|AAM18480.1|AF492389_1|Sm14 fatty acid-binding protein isoform T20 [Schistosoma mansoni]; gb|AAT39384.1||fatty acid binding protein 15 [Schistosoma bovis];	-0,246871758	-0,243071339	-0,950628782	-0,869929981	-0,55840087	1,5	DOWN

477	C610449.1	ref|XP_974489.2||PREDICTED: similar to Ankyrin repeat domain-containing protein 17 (Gene trap ankyrin repeat protein) (Serologically defined breast cancer antigen NY-BR-16) [Tribolium castaneum]; ref|XP_393472.3||PREDICTED: similar to ankyrin repeat domain protein 17 isoform a [Apis mellifera];	-0,334925688	-0,506935508	-0,609820773	-0,981156552	-0,558378141	1,5	DOWN
478	C609610.1	No match	-0,564289129	-0,445764395	-0,667775416	-0,547865951	-0,55607754	1,5	DOWN
479	C600581.1	gb|AAX26169.2||SJCHGC04821 protein [Schistosoma japonicum]; ref|XP_001949000.1||PREDICTED: similar to Protein ADRM1 homolog (p42E) [Acyrthosiphon pisum]; ref|XP_968223.1||PREDICTED: similar to AT08455p [Tribolium castaneum]; ref|XP_001632164.1||predicted protein [Nematostella vectensis] gb|EDO40101.1| predicted protein [Nematostella vectensis];	-0,56098336	-0,649637281	-0,549222147	-0,369855247	-0,555102754	1,5	DOWN
480	C609632.1	No match	-0,52018272	-0,601473353	-0,513348633	-0,589268214	-0,554725467	1,5	DOWN
481	C612205.1	gb|AAW27894.1||SJCHGC05945 protein [Schistosoma japonicum];	-0,691848589	-0,299056061	-0,624473714	-0,479066743	-0,551770229	1,5	DOWN
482	C601522.1	gb|ACE06902.1||unknown [Schistosoma japonicum]; gb|AAP05958.1||similar to GenBank Accession Number AE003576 CG3652 gene product in Drosophila melanogaster [Schistosoma japonicum];	-0,573944618	-0,405977205	-0,993545803	-0,529315262	-0,55162994	1,5	DOWN
483	C601838.1	gb|AAX24832.2||SJCHGC09189 protein [Schistosoma japonicum];	-0,612720802	-0,573684245	-0,5290195	-0,520322003	-0,551351873	1,5	DOWN
484	C600255.1	gb|AAW26014.1||SJCHGC04863 protein [Schistosoma japonicum]; ref|NP_001026661.1||TBC1 domain family, member 22A [Gallus gallus] emb|CAG31060.1| hypothetical protein [Gallus gallus]; ref|XP_001500326.1||PREDICTED: similar to MGC137912 protein [Equus caballus]; gb|EAX03942.1||TBC1 domain family, member 22B, isoform CRA_d [Homo sapiens];	-0,389157728	-0,564850421	-0,659077953	-0,532975004	-0,548912713	1,5	DOWN
485	C600527.1	gb|AAX28087.2||SJCHGC00998 protein [Schistosoma japonicum]; gb|ABA40363.1||SJCHGC09384 protein [Schistosoma japonicum]; ref|NP_001069240.1||WD repeat domain 53 [Bos taurus] sp|Q32KQ2.1|WDR53_BOVIN WD repeat-containing protein 53 gb|AAI09980.1| WD repeat domain 53 [Bos taurus]; ref|XP_001364201.1||PREDICTED: similar to WD repeat domain 53 [Monodelphis domestica];	-0,843098247	-0,472959361	-0,592478587	-0,50452195	-0,548500269	1,5	DOWN
486	C610977.1	No match	-0,475320333	-0,621627147	-0,624316627	-0,354967604	-0,54847374	1,5	DOWN
487	C607402.1	sp|Q01137|SODC_SCHMA|Superoxide dismutase [Cu-Zn] gb|AAA29936.1| superoxide dismutase; gb|AAC14467.1||Cu/Zn-superoxide dismutase [Schistosoma mansoni]; pdb|1TO4|A|Chain A, Structure Of The Cytosolic Cu,Zn Sod From S. Mansoni pdb|1TO4|B Chain B, Structure Of The Cytosolic Cu,Zn Sod From S. Mansoni pdb|1TO4|C Chain C, Structure Of The Cytosolic Cu,Zn Sod From S. Mansoni pdb|1TO4|D Chain D, Structure Of The Cytosolic Cu,Zn Sod From S. Mansoni pdb|1TO5|A Chain A, Structure Of The Cytosolic Cu,Zn Sod From S. Mansoni pdb|1TO5|B Chain B, Structure Of The Cytosolic Cu,Zn Sod From S. Mansoni pdb|1TO5|C Chain C, Structure Of The Cytosolic Cu,Zn Sod From S. Mansoni pdb|1TO5|D Chain D, Structure Of The Cytosolic Cu,Zn Sod From S. Mansoni; gb|AAA29935.1||superoxide dismutase;	-0,430240677	-0,321798817	-0,957808292	-0,66650072	-0,548370699	1,5	DOWN

488	C610255.1	gb|AAW26926.1||SJCHGC05742 protein [Schistosoma japonicum]; emb|CAG05279.1||unnamed protein product [Tetraodon nigroviridis];	-0,367123133	-0,464635552	-0,666017955	-0,631921248	-0,5482784	1,5	DOWN
489	C608373.1	gb|AAC46898.1||similar to human carbonyl reductase (NADPH), PIR Accession Number A61271; Method: conceptual translation supplied by author; gb|AAW25778.1||SJCHGC00683 protein [Schistosoma japonicum]; ref|XP_001641607.1||predicted protein [Nematostella vectensis] gb|EDO49544.1| predicted protein [Nematostella vectensis]; gb|AAL16062.1|AF420278_1|carbonyl reductase [Anguilla japonica];	-0,652188438	-0,763883813	-0,443276959	-0,432464651	-0,547732699	1,5	DOWN
490	C612352.1	No match	-0,840541366	-0,873349511	-0,251186278	-0,21364632	-0,545863822	1,5	DOWN
491	C606731.1	No match	-0,893309444	-0,570925014	-0,51954135	-0,456934908	-0,545233182	1,5	DOWN
492	C609954.1	ref|XP_973078.1||PREDICTED: similar to AGAP007538-PA [Tribolium castaneum]; ref|NP_035917.2||autocrine motility factor receptor [Mus musculus] gb|AAH34538.1| Autocrine motility factor receptor [Mus musculus] gb|AAH40338.1| Autocrine motility factor receptor [Mus musculus] dbj|BAE41974.1| unnamed protein product [Mus musculus]; ref|NP_998328.1||autocrine motility factor receptor [Danio rerio] gb|AAH57411.1| Autocrine motility factor receptor [Danio rerio]; gb|AAD56721.1|AF124144_1|autocrine motility factor receptor [Mus musculus];	-0,537828717	-0,746412338	-0,549427284	-0,50839747	-0,543628001	1,5	DOWN
493	C605625.1	gb|AAA19732.1||glucose transport protein; gb|AAX26581.2||SJCHGC02813 protein [Schistosoma japonicum]; gb|AAB05920.1||glucose transporter TGTP2 [Taenia solium]; ref|NP_001036186.1||solute carrier family 2 (facilitated glucose transporter), member 2 [Danio rerio] gb|AAZ43092.1| glucose transporter 2 [Danio rerio];	-0,717931426	-0,577564398	-0,502487601	-0,435606237	-0,540026	1,5	DOWN
494	C601197.1	gb|AAX30542.1||SJCHGC04779 protein [Schistosoma japonicum]; gb|ABK22541.1||unknown [Picea sitchensis]; ref|XP_001187173.1||PREDICTED: similar to SNRPF protein [Strongylocentrotus purpuratus]; ref|XP_796806.2||PREDICTED: similar to SNRPF protein, partial [Strongylocentrotus purpuratus];	-0,570896261	-0,505769599	-0,575927609	-0,394504931	-0,53833293	1,5	DOWN
495	C601689.1	gb|AAX25664.2||SJCHGC03360 protein [Schistosoma japonicum]; ref|XP_001952785.1||PREDICTED: similar to beta1,4 mannosyltransferase [Acyrthosiphon pisum]; gb|EDV19433.1||hypothetical protein TRIADDRAFT_33634 [Trichoplax adhaerens]; ref|XP_623777.2||PREDICTED: similar to beta-1,4-mannosyltransferase [Apis mellifera];	-0,794542789	-0,348316407	-0,555235711	-0,519550451	-0,537393081	1,5	DOWN
496	C610592.1	gb|AAX25907.2||SJCHGC09404 protein [Schistosoma japonicum];	0,582847299	0,431967193	0,568732694	0,887551061	0,575789997	1,5	UP
497	C604083.1	No match	0,70485699	0,713294937	0,469678973	0,443485847	0,587267982	1,5	UP
498	C610809.1	gb|AAX25746.2||SJCHGC05130 protein [Schistosoma japonicum]; gb|EDW72378.1||GK20895 [Drosophila willistoni]; gb|EDW91955.1||GE14079 [Drosophila yakuba]; gb|EDW31890.1||GL11356 [Drosophila persimilis];	0,79856799	0,734062214	0,492819019	0,479336162	0,613440617	1,5	UP

499	C606657.1	gb|AAX27370.2||SJCHGC05548 protein [Schistosoma japonicum]; ref|NP_001090456.1||hypothetical protein LOC779369 [Xenopus laevis] sp|Q6GPC6|TB1RB_XENLA F-box-like/WD repeat-containing protein TBL1XR1-B (Transducin beta-like 1X-related homolog 1-B) (Nuclear receptor corepressor/HDAC3 complex subunit TBLR1-B) (TBL1-related protein 1-B) gb|AAH73215.1| MGC80502 protein [Xenopus laevis]; ref|XP_424555.2||PREDICTED: hypothetical protein [Gallus gallus]; ref|NP_001120367.1||hypothetical protein LOC100145441 [Xenopus tropicalis] gb|AAI61057.1| Unknown (protein for MGC:184829) [Xenopus tropicalis];	0,510740533	0,433362485	0,820691738	0,725695431	0,618217982	1,5	UP
500	C607838.1	gb|AAX28366.2||SJCHGC04923 protein [Schistosoma japonicum]; ref|NP_499890.2||T21D12.3 [Caenorhabditis elegans] gb|AAC48091.2| Hypothetical protein T21D12.3 [Caenorhabditis elegans]; ref|XP_001671977.1||Hypothetical protein CBG10545 [Caenorhabditis briggsae AF16] emb|CAP29955.1| Hypothetical protein CBG10545 [Caenorhabditis briggsae]; ref|XP_001897165.1||WW domain containing protein [Brugia malayi] gb|EDP34005.1| WW domain containing protein [Brugia malayi];	0,92923856	0,379304522	0,683728639	0,576503088	0,630115864	1,5	UP
501	C611430.1	No match	0,725784894	0,712694652	0,511791322	0,548447257	0,630570955	1,5	UP
502	C602742.1	No match	0,713579092	0,651261449	0,519988334	0,615319759	0,633290604	1,6	UP
503	C609745.1	No match	0,978049607	0,583970157	0,703205829	0,358333903	0,643587993	1,6	UP
504	C601437.1	No match	0,821603348	0,706224159	0,58995484	0,470754327	0,6480895	1,6	UP
505	C707555.1	ref|XP_001372990.1||PREDICTED: similar to multidrug resistance protein 2; MRP2 [Monodelphis domestica]; gb|EDL41913.1||ATP-binding cassette, sub-family C (CFTR/MRP), member 2 [Mus musculus]; ref|NP_038834.2||ATP-binding cassette, sub-family C, member 2 [Mus musculus] gb|AAL36986.1|AF282773_1 sub-family C member 2 ATP-binding cassette protein [Mus musculus]; sp|Q8VI47|MRP2_MOUSE|Canalicular multispecific organic anion transporter 1 (ATP-binding cassette sub-family C member 2) gb|AAL36985.1|AF282772_1 sub-family C member 2 ATP-binding cassette protein [Mus musculus];	0,920328291	0,691603702	0,606901739	0,467461095	0,649252721	1,6	UP
506	C612590.1	No match	0,7036255	0,667244993	0,632998305	0,605454397	0,650121649	1,6	UP
507	C610554.1	ref|XP_001624897.1||predicted protein [Nematostella vectensis] gb|EDO32797.1| predicted protein [Nematostella vectensis]; ref|XP_001620718.1||hypothetical protein NEMVEDRAFT_v1g222786 [Nematostella vectensis] gb|EDO28618.1| predicted protein [Nematostella vectensis]; gb|EDV28510.1||hypothetical protein TRIADDRAFT_19202 [Trichoplax adhaerens]; gb|AAI42592.1||Checkpoint protein [Xenopus laevis];	0,599851215	0,77456969	0,442319012	0,702816421	0,651333818	1,6	UP
508	C607914.1	gb|AAW24719.1||SJCHGC06079 protein [Schistosoma japonicum];	0,779666181	0,733899351	0,586940969	0,509901896	0,66042016	1,6	UP
509	C604775.1	No match	0,895432933	0,861612153	0,463441062	0,45539218	0,662526608	1,6	UP

510	C608834.1	ref|NP_001107473.1||hypothetical protein LOC100135324 [Xenopus tropicalis] emb|CAK11162.1| novel protein (zgc:114074) [Danio rerio] gb|AAI52590.1| Tmem115 protein [Danio rerio] gb|AAI57623.1| Unknown (protein for MGC:181014) [Xenopus tropicalis]; ref|NP_001028917.1||transmembrane protein 115 [Danio rerio] gb|AAH97136.1| Transmembrane protein 115 [Danio rerio]; gb|AAI09436.1||Tmem115 protein [Danio rerio]; ref|XP_001518365.1||PREDICTED: hypothetical protein [Ornithorhynchus anatinus];	0,826650308	0,788796872	0,549654111	0,53488998	0,669225492	1,6	UP
511	C603612.1	No match	0,838393469	0,449050605	0,568413716	0,784252059	0,676332888	1,6	UP
512	C607124.1	gb|AAX28476.2||SJCHGC03500 protein [Schistosoma japonicum]; ref|XP_974514.1||PREDICTED: similar to putative cholesterol transporter BmStart1 [Tribolium castaneum]; ref|NP_001006799.1||START domain containing 3 [Xenopus tropicalis] gb|AAH76666.1| START domain containing 3 [Xenopus tropicalis]; ref|NP_571737.1||START domain containing 3 [Danio rerio] sp|Q9DFS4.2|STAR3_DANRE StAR-related lipid transfer protein 3 (START domain-containing protein 3) (StARD3) (MLN64-like protein) gb|AAH56766.1| START domain containing 3 [Danio rerio] emb|CAK04971.2| START domain containing 3 [Danio rerio];	0,817945713	0,797367436	0,577276275	0,602302778	0,699835107	1,6	UP
513	C604589.1	ref|XP_001663199.1||brefeldin a-inhibited guanine nucleotide-exchange protein [Aedes aegypti] gb|EAT34779.1| brefeldin a-inhibited guanine nucleotide-exchange protein [Aedes aegypti]; ref|XP_001846441.1||brefeldin A-inhibited guanine nucleotide-exchange protein 1 [Culex pipiens quinquefasciatus] gb|EDS43578.1| brefeldin A-inhibited guanine nucleotide-exchange protein 1 [Culex quinquefasciatus]; ref|XP_319652.3||AGAP008906-PA [Anopheles gambiae str. PEST] gb|EAA14874.4| AGAP008906-PA [Anopheles gambiae str. PEST]; emb|CAG00665.1||unnamed protein product [Tetraodon nigroviridis];	0,936987878	0,807889232	0,601560939	0,440349309	0,704725086	1,6	UP
514	C718538.1	gb|EDL26904.1||mCG9866 [Mus musculus]; ref|NP_035019.1||nebulin [Mus musculus]; emb|CAM21137.1||nebulin [Mus musculus] emb|CAM17920.1| nebulin [Mus musculus]; ref|NP_001101201.1||nebulin [Rattus norvegicus] gb|EDM00439.1| nebulin (predicted) [Rattus norvegicus];	0,467692317	0,705251841	0,83789667	0,72165147	0,713451656	1,6	UP
515	C600335.1	ref|XP_540384.2||PREDICTED: similar to component of oligomeric golgi complex 5 isoform 1 [Canis familiaris]; ref|XP_001509241.1||PREDICTED: similar to component of oligomeric golgi complex 5 [Ornithorhynchus anatinus]; ref|XP_001076141.1||PREDICTED: similar to component of oligomeric golgi complex 5 isoform 1 [Rattus norvegicus]; ref|XP_001163243.1||PREDICTED: component of oligomeric golgi complex 5 isoform 3 [Pan troglodytes];	0,985779499	0,369668891	0,93572651	0,502815567	0,719271039	1,6	UP
516	C606481.1	No match	0,894351677	0,962555422	0,669389237	0,711528535	0,802940106	1,7	UP
517	C610534.1	No match	0,801126245	0,792410649	1,068879471	1,258068235	0,935002858	1,9	UP

= 2 ^ \|median log2 (test/control)\|

** Up- or down-regulation refers to a significant (q< 0.01) increase or decrease (respectively) in the expression level of that gene in *S. mansoni* worms recovered from mice vaccinated with rSm29 plus adjuvant (test), in relation to control mice injected only with adjuvant (control).
