# Supplementary material for: Schistosoma mansoni Tegument Protein Sm29 Is Able to Induce a Th1-Type of Immune Response and Protection against Parasite Infection
Source: PLoS Negl Trop Dis. 2008 Oct 1;2(10):e308. doi: 10.1371/journal.pntd.0000308 (PMC2553283; doi:10.1371/journal.pntd.0000308)
Supplement: Table S2 — List of primers used for validation by real-time RT-PCR (0.04 MB PDF) [file pntd.0000308.s003.pdf]

Supporting Information Table S2 – Primers used in validation by Real Time RT-PCR

Contig	Primer 1	Primer 2
C600716 (CD36)	CACAGATCGTGCTGGCTTAATG	GCACTTTCCCGACGTTTGAA
C600861 (B-cell-RAP)	CCTCATGAAATGCGTCCAGAA	AACCACACGAATAATGAAAATCCA
C601295 (Sm23)	TGATAGTGAGCTTTCTGGGTTGTT	CCAACTCAGCAATCAGAAGTACGA
C601665 (Sm14)	ACACAACTTCGATGCTGTCATGT	CCCCATCCATTGTGAAGGTT
C607402 (Superox)	TGAAAGTGGTGTGGATTTTCGT	TTACGCCAGCTGTACCAGTCA
C714516 (TGF-β-RIP)	GGTGGCTTGACGTAGATTGGA	GGTGTTTGTGGATTTTCCTGTCT
Actin	CGTTGGACGACCTCGACAT	TGTCTTTCTGACCCATACCAACC
